# Modulation of tumor inflammatory signaling and drug sensitivity by CMTM4

**DOI:** 10.1038/s44318-024-00330-y

**Published:** 2025-02-13

**Authors:** Yitian Xu, Kyeongah Kang, Brian A Coakley, Samuel Eisenstein, Arshiya Parveen, Sunny Mai, Yuan Shuo Wang, Junjun Zheng, Debasish Boral, Junhua Mai, William Pan, Licheng Zhang, Stuart A Aaronson, Bingliang Fang, Celia Divino, Bin Zhang, Won-Min Song, Mien-Chie Hung, Ping-Ying Pan, Shu-Hsia Chen

**Affiliations:** 1 Immunotherapy Research Center, Houston Methodist Research Institute, Houston, TX 77030 USA; 2 Neal Cancer Center of Excellence, Houston Methodist Research Institute, Houston, TX 77030 USA; 3 Department of Oncological Sciences, Icahn School of Medicine at Mount Sinai, New York, NY USA; 4 Department of Surgery, Icahn School of Medicine at Mount Sinai, New York, NY 10029 USA; 5 Department of Nanomedicine, Houston Methodist Research Institute, Houston, TX 77030 USA; 6 Department of Thoracic and Cardiovascular Surgery, MD Anderson Cancer Center, Houston, TX 77030 USA; 7 Department of Genetics and Genomic Sciences, Icahn School of Medicine at Mount Sinai, New York, NY 10029 USA; 8 Graduate Institute of Biomedical Sciences, Research Center for Cancer Biology and Center for Molecular Medicine, China Medical University, Taichung, Taiwan; 9 Department of Physiology, Biophysics, and Systems Biology, Weill Cornell Medical Science and Graduate School of Medical Sciences, New York, NY 10065 USA; 10 Graduate and professional school at Texas A&M University, 400 Bizzell St., College Station, TX 77840 USA

**Keywords:** CMTM4, Tumor Microenvironment, Tumor-associated Inflammation, EGFR, Drug Resistance, Cancer, Immunology, Signal Transduction

## Abstract

Although inflammation has been widely associated with cancer development, how it affects the outcomes of immunotherapy and chemotherapy remains incompletely understood. Here, we show that CKLF-like MARVEL transmembrane domain-containing member 4 (CMTM4) is highly expressed in multiple human and murine cancer types including Lewis lung carcinoma, triple-negative mammary cancer and melanoma. In lung carcinoma, loss of CMTM4 significantly reduces tumor growth and impairs NF-κB, mTOR, and PI3K/Akt pathway activation. Furthermore, we demonstrate that CMTM4 can regulate epidermal growth factor (EGF) signaling post-translationally by promoting EGFR recycling and preventing its Rab-dependent degradation. Consequently, CMTM4 knockout sensitizes human lung tumor cells to EGFR inhibitors. In addition, CMTM4 knockout tumors stimulated with EGF show a decreased ability to produce inflammatory cytokines including granulocyte colony-stimulating factor (G-CSF), leading to decreased recruitment of polymorphonuclear myeloid-derived suppressor cells (PMN-MDSCs) and therefore establishing a less suppressive tumor immune environment in both lung and mammary cancers. We also present evidence indicating that CMTM4-targeting siRNA-loaded liposomes reduce lung tumor growth in vivo and prolong animal survival. Knockout of CMTM4 enhances immune checkpoint blockade or chemotherapy to further reduce lung tumor growth. These data suggest that CMTM4 represents a novel target for the inhibition of tumor inflammation, and improvement of the immune response and tumor drug sensitivity.

## Synopsis

Despite the established links between inflammation and cancer development, the effects of inflammatory signaling on cancer therapy are not completely understood. This study shows that inhibiting the function of the tetra-transmembrane protein CMTM4 can sensitize tumor cells to EGFR inhibition and restrict tumor growth by repressing tumor inflammation.

- CMTM4 is highly expressed in multiple cancer types including Lewis lung carcinoma, triple-negative breast cancer, and melanoma.
- Loss of CMTM4 reduces tumor growth and NF-κB, mTOR, and PI3K/Akt pathway activation.
- CMTM4 maintains EGFR signaling by promoting EGFR recycling and preventing its degradation.
- Under EGF stimulation, CMTM4 knockout tumors exhibit decreased granulocyte colony-stimulating factor (G-CSF) production and attenuated recruitment of polymorphonuclear myeloid-derived suppressor cells (PMN-MDSCs) to the tumor environment.
- Inhibiting CMTM4 function reduces tumor growth in vivo and prolongs animal survival, and further synergizes with inhibitors of EGFR and the immune checkpoint.

## Abstract (web summary)

Depletion of the tetra-transmembrane protein CMTM4 sensitizes tumor cells to EGFR inhibition and limits tumor growth by reducing the production of inflammatory cytokines and the recruitment of immune-suppressive cells.

## Graphical abstract

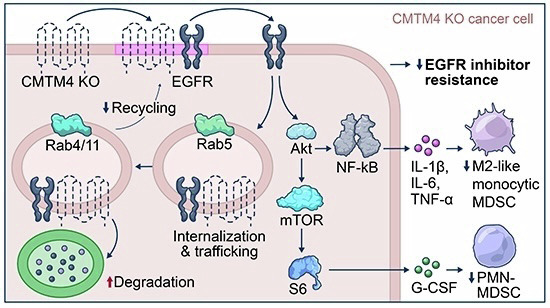

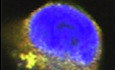

## Introduction

Inflammation has been recognized as a hallmark of cancer and is linked to tumor initiation and progression (Greten and Grivennikov, 2019; Maiorino and Egeblad, 2019). Tumor-associated inflammation has also been shown to promote angiogenesis, metastasis, and resistance to chemotherapy, and to subvert immune surveillance (Crusz and Balkwill, 2015; Greten and Grivennikov, 2019). Activation of receptor tyrosine kinases (RTKs) can induce tumor inflammation, proliferation, and survival and subsequently modulate the tumor microenvironment (Schlessinger, 2000). The epidermal growth factor receptor (EGFR) is one of the critical RTKs that is involved in cancer cell proliferation, migration, and survival (Schlessinger, 2000; Wee and Wang, 2017). After engagement by its ligand, EGFR is endocytosed and recycled back to the surface for proper function (Lo and Hung, 2006). The EGFR exerts its biological effects via a cytoplasmic/traditional pathway or nuclear mode of action (Lo and Hung, 2006). The cytoplasmic/traditional signal pathway consists of PLCγ-CaMK/PKC, Ras-Raf-MAPK, PI3K-Akt, and STATs leading to tumorigenesis, tumor proliferation, metastasis, chemo-resistance, and radio-resistance (Wee and Wang, 2017). The nuclear pathway can be initiated by ligand binding followed by nuclear translocation (Lin et al, 2001). Nuclear EGFR increases cyclin D1, inducible nitric oxide synthase (iNOS), and MYBL2 (B-Myb), all of which are involved in cancer proliferation (Lo and Hung, 2006). Several FDA-approved tyrosine kinase inhibitors (TKIs) exist that interfere with EGFR activation. Despite these achievements, recent studies have shown that treatment of EGFR TKIs leads to the development of acquired resistance through target gene mutations, activation of alternative RTK signaling pathways, histological transformation, or intra-tumor heterogeneity (Chong and Janne, 2013). Thus, the efficient interruption of RTK activation through novel mechanisms may have important implications in cancer therapeutics which is urgently needed.

Chemokine-like factor (CKLF)-like MARVEL transmembrane domain-containing family 4 (CMTM4) belongs to the CMTM family consisting of nine members, CKLF and CMTM1-8. Among CMTM family members, CMTM4 is the most conserved member and has been implicated to play a role in tumor progression and modulation of the tumor microenvironment (Li et al, 2015b; Plate et al, 2010b; Wang et al, 2009). Despite being discovered many years ago, the precise role of CMTM4 in cancer progression and its underlying mechanisms remain largely uncharacterized. Interestingly, while CMTM4 is expressed only in low and variable amounts in multiple normal human tissues, it is universally expressed in a multitude of human cancers (Plate et al, 2010b; Wang et al, 2009). Recently, CMTM4 has been shown to regulate PD-L1 expression on tumor cells and IL-17 receptor C on stromal cells (Knizkova et al, 2022; Mezzadra et al, 2017). However, the detailed function of CMTM4 on cancer cells has not been explored. In this study, we identified a novel role of CMTM4 in promoting EGFR recycling. Knockout or knockdown of CMTM4 in tumor cells resulted in inhibition of EGFR signaling, mTOR and PI3K/Akt pathways. Moreover, inhibition of EGFR signaling decreased productions of inflammatory cytokines, including G-CSF. As a result, tumor infiltration of PMN-MDSCs was significantly decreased. Further, treatment of liposomes encapsulating CMTM4-specific siRNA significantly reduced tumor growth in vivo, which can further synergize with EGFR inhibitor to enhance the therapeutic outcomes. Our studies identified CMTM4 as a potential novel target for cancer therapy and controlling tumor inflammation.

## Results

### CMTM4 is highly expressed in cancers and can be a prognostic marker

**Figure 1:**
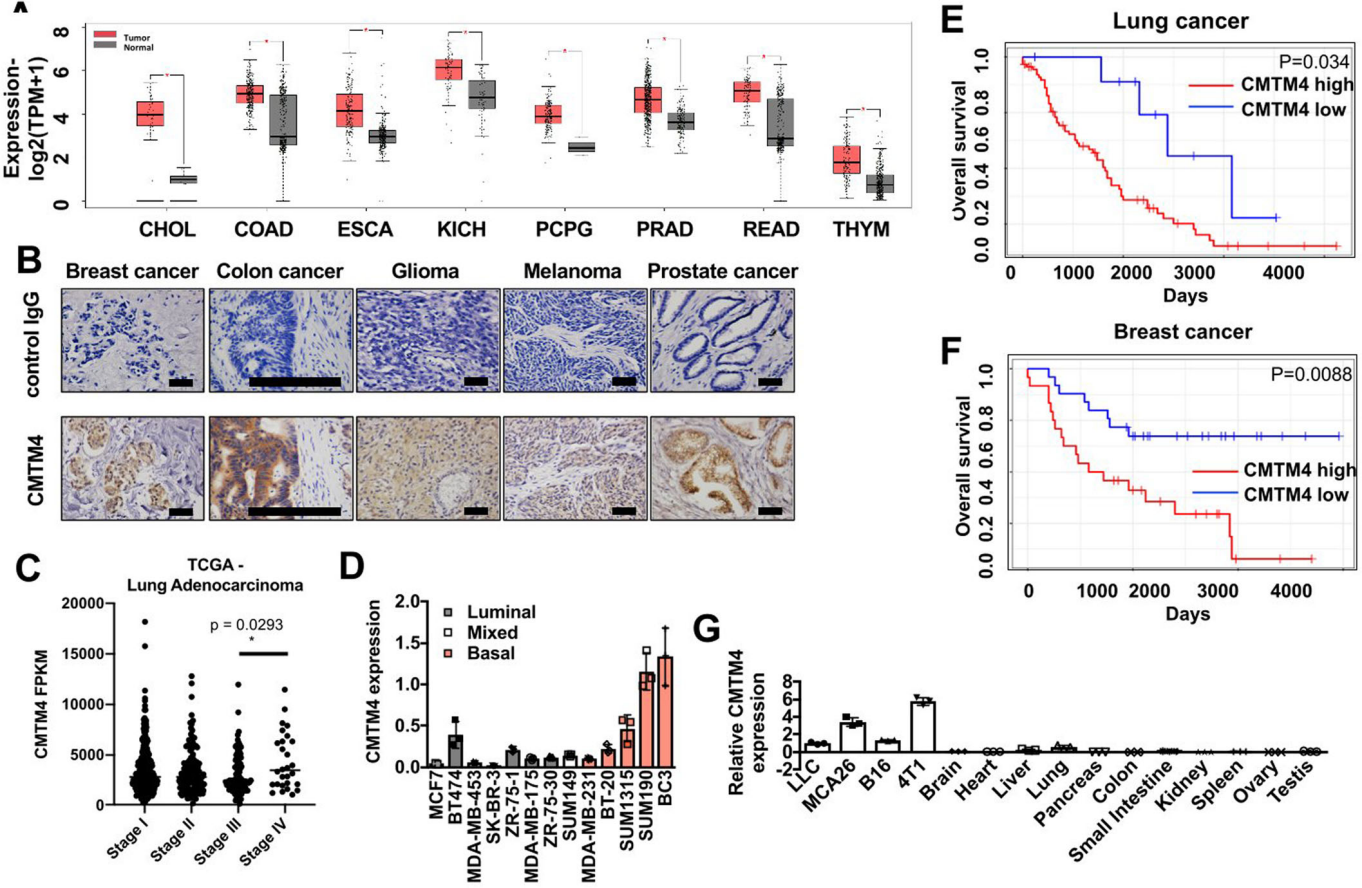
CMTM4 is highly expressed in human carcinoma samples and is a poor prognostic factor for survival in these patients. (**A**) CMTM4 expression in human cancer tissues vs. normal tissues was obtained from TCGA and GTEx databases. CHOL: cholangiocarcinoma (*n* = 36 for Tumor, *n* = 9 for normal), COAD: colon adenocarcinoma (*n* = 275 for Tumor, *n* = 349 for normal), ESCA: esophageal carcinoma (*n* = 182 for Tumor, *n* = 286 for normal), KICH: kidney chromophobe (*n* = 66 for Tumor, *n* = 53 for normal), PCPG: pheochromocytoma and paraganglioma (*n* = 182 for Tumor, *n* = 3 for normal), PRAD: prostate adenocarcinoma (*n* = 492 for Tumor, *n* = 152 for normal), READ: rectum adenocarcinoma (*n* = 92 for Tumor, *n* = 318 for normal), THYM: thymoma (*n* = 118 for Tumor, *n* = 339 for normal). Box plots show CMTM4 expression at transcripts per million (TPM) in log2-scale. The expression level of CMTM4 ranging from 0 (minimum) to 8 (maximum) across sleeted tumor and normal tissues. Individual boxplots were shown at 95/5 percentile whiskers and 75/25 percentile box bounds with median value at the box center. (**B**) Paraffin-embedded human carcinoma sections were stained with anti-CMTM4 antibody (40x). scale bars = 50 μm. (**C**) CMTM4 expression in the different stages of lung adenocarcinoma obtained from TCGA was analyzed. *P* value calculated by Mann–Whitney test. *n* = 517. (**D**) CMTM4 expression in various human breast cancer cell lines was determined by real-time PCR. (**E**, **F**) Lung cancer (**E**, *P* = 0.034) and breast cancer (**F**, *P* = 0.0088) patient data were obtained from dataset GSE26939 and GSE37751, respectively, and divided into CMTM4 high (red) vs. low (blue) expression groups based on median expression values. The Kaplan–Meier plot was drawn based on expression level and correlated with overall survival rates. *P* value calculated by Log-rank test. (**G**) CMTM4 expression in murine cancer cell lines and normal tissues was assessed by qRT-PCR analysis. All samples were quantitated and standardized by LLC tumor expression in triplicate with data representing the mean ± SD. Source data are available online for this figure.

To identify potential regulators of cancer-associated inflammation, we performed real-time quantitative PCR (RT-qPCR) superarrays to profile gene expression in different tumor types. CMTM4 was identified as one of the highly expressed genes in multiple tumors (Appendix Fig. S1) and has been implicated in tumor progression (Chrifi et al, 2019; Li et al, 2015b; Mezzadra et al, 2017; Plate et al, 2010b). We further determined whether CMTM4 expression was increased in human carcinomas by comparing CMTM4 expression levels between tumor and normal tissue with same origin in multiple human cancer types from the TCGA and Genotype Tissue Expression (GTEx) databases. We found that CMTM4 expression was significantly higher in cholangiocarcinoma (CHOL), colon adenocarcinoma (COAD), esophageal carcinoma (ESCA), kidney chromophobe (KICH), pheochromocytoma and paraganglioma (PCPG), prostate adenocarcinoma (PRAD), rectum adenocarcinoma (READ), and thymoma (THYM) when compared to expression in normal tissues (Fig. 1A). We also confirmed CMTM4 expression in a variety of human carcinoma tissue biopsies (Fig. 1B; Appendix Fig. S2), including breast cancer, colon cancer, glioma, melanoma, and prostate cancer. Furthermore, in advanced lung adenocarcinoma, CMTM4 expression was significantly higher in stage IV tumor compared to early-stage tumors, suggesting a positive correlation between CMTM4 expression and high-grade tumors (Fig. 1C). Interestingly, invasive basal and inflammatory breast cancer cell lines exhibited higher CMTM4 expression levels (e.g., SUM190 and BC3) when compared to luminal and mixed breast cancer cell lines, indicating a positive association between CMTM4 expression and tumor aggressiveness/poor outcome (Fig. 1D). Lung and breast cancer patients with higher CMTM4 expression showed a significantly reduced survival rate in comparison to patients with lower CMTM4 expression (Fig. 1E,F). Interestingly, ER, PR or Her2 negative breast cancer patients showed significantly reduced survival in CMTM4 high patients when compared to CMTM4 low patients (Appendix Fig. S3A, upper panel). ER, PR or Her2 positive breast cancer patients showed no significant differences in survival between CMTM4 high and low patients (Appendix Fig. S3A, lower panel). A significant survival difference between CMTM4 high and low patients was found in lung cancer patients with Grade 2 and Grade 3, but not with Grade 1 (Appendix Fig. S3B). Adrenal, brain, head, and neck cancer, as well as leukemia patients also showed lower survival rates in the patient group that had higher CMTM4 expression (Appendix Table S1). A series of quantitative RT-PCR assays confirmed that the expression of CMTM4 was significantly higher in tumor cell lines than in the normal tissues analyzed (Fig. 1G). Overall, these results suggest that CMTM4 was highly expressed in multiple cancer types, correlates with tumor progression, and its expression can be a poor prognostic factor in multiple cancer types.

### CMTM4 promotes tumor progression in vivo and activation of Akt/mTOR signaling and NF-κB pathway

**Figure 2:**
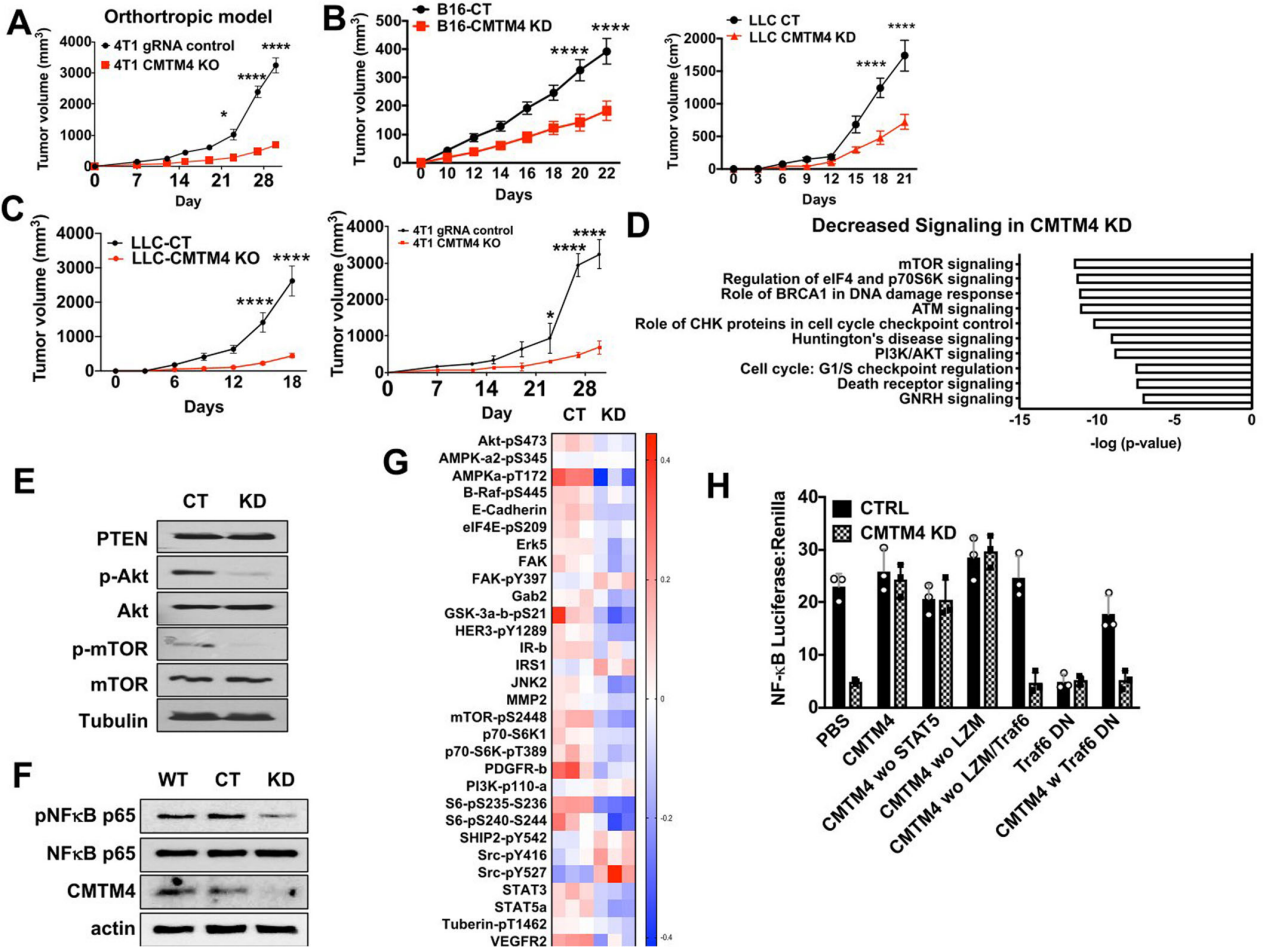
CMTM4 knockdown results in a reduction in tumor growth in vivo. (**A**) 4T1 control and CMTM4 KO tumors were implanted to the mammary fat pad of Balb/c mice (*n* = 3). Tumor volume was measured every 7 days. Data representing the mean ± SD. *P* = 0.024 at Day 23, *P* < 0.0001 at both Day 27 and Day 30. (**B**) Control or CMTM4 KD tumor cells were inoculated into the flanks C57BL/6 (B16 or LLC cells) mice (*n* = 4). Tumor size was measured every 2–3 days. Data representing the mean ± SD. For B16 tumors, *P* < 0.0001 at both Day 20 and Day 30. For LLC tumors, *P* < 0.0001 at both Day 18 and Day 21. (**C**) Control or CMTM4 KO LLC and 4T1 cells were inoculated into C57BL/6 (LLC cells) or BALB/c (4T1) mice (*n* = 5). Tumor growth was measured every 3–4 days. Data representing the mean ± SD. For LLC tumors, *P* < 0.0001 at both Day 15 and Day 18. For 4T1 tumors, *P* = 0.0017 at Day 23, *P* < 0.0001 at both Day 27 and Day 30. (**D**) Decreased signaling pathways analyzed by IPA in CMTM4 KD LLC cells compared to control LLC cells. (**E**) Akt/mTOR signaling was evaluated by western blot in LLC control and CMTM4 KD cells. (**F**) Phosphorylation of NF-κB in control and CMTM4 KD LLC cells was detected by western blot. (**G**) Heat-map visualization of normalized DEGs associated with receptor tyrosine kinase (RTK)-related gene expression by CMTM4 KD in LLC cells from RPPA. (**H**) NF-κB activity was determined in control and CMTM4 KD LLC cells transfected with plasmids carrying pcDNA-CMTM4, STAT5 deleted CMTM4, LZM (leucine-zipper motif) deleted CMTM4, LZM/Traf6 deleted CMTM4, Traf6 dominant-negative mutation (TRAF6 DN) or pcDNA-CMTM4 with TRAF6 DN. Samples were quantitated in triplicate with data representing the mean ± SD. All experiments were repeated twice with similar results. *P* value calculated by two-way ANOVA test. *P < 0.05. **P < 0.01. ****P < 0.0001. Source data are available online for this figure.

To investigate CMTM4’s function in tumor progression, CRISPR-cas9 knockout (KO) or shRNA knockdown (KD) of CMTM4 in different tumor cell lines was performed (Appendix Fig. S4A,B). We orthotopically implanted control and CMTM4 KO breast cancer 4T1 cells to the mammary fat pad of mice and observed significant growth defect of CMTM4 KO tumor in vivo (Fig. 2A). Loss of CMTM4 in lung cancer (LLC), melanoma (B16) or 4T1 was associated with significantly reduced subcutaneous tumor growth in vivo (Fig. 2B,C) despite a similar growth rate in vitro (Appendix Fig. S4C).

To identify genes and pathways that are affected by CMTM4 knockdown, we performed RNA sequencing (RNAseq) and Ingenuity Pathway Analysis (IPA) and found that the mTOR signaling and PI3K/Akt signaling pathways were significantly inhibited in CMTM4 KD cells when compared to control cells (Fig. 2D). Western blot confirmed the loss of CMTM4 resulted in reduced phosphorylation of Akt and mTOR (Fig. 2E; Appendix Fig. S5A,B). The expression of PTEN, which is a natural inhibitor of PI3K/Akt, was not affected by CMTM4 KD (Fig. 2E; Appendix Fig. S5B), suggesting that reduced activation of the Akt/mTOR pathway by CMTM4 KD is not through PTEN regulation. Furthermore, downstream of Akt activation, i.e., NF-κB phosphorylation, was also inhibited by CMTM4 KD (Fig. 2F). Moreover, knockout of CMTM4 in breast cancer cell line 4T1 also confirmed the reduced Akt and NF-κB phosphorylation (Appendix Fig. S5C). Reverse phase protein array (RPPA) data analysis also confirmed downregulation of RTK downstream pathway in CMTM4 KD cells including mTOR, STAT3 and Erk (Fig. 2G). Inhibition of the NF-κB pathway by CMTM4 KD was confirmed using NF-κB luciferase reporter cells (Fig. 2H). Importantly, the inhibition of NF-κB activation by CMTM4 KD was restored by transfection with full-length CMTM4 (Fig. 2H).

To determine which domain of CMTM4 was critical for NF-κB activation, we used Eukaryotic Linear Motif (ELM) resource to predict potential binding domains of CMTM4 and found potential binding domains of STAT5, TRAF6 and a leucine-zipper motif (LZM). To assess the potential functions of these domain, we generated truncated CMTM4 with deletions in one or more predicted binding domains and transfected into CMTM4 KD cells. CMTM4 with deletions in the STAT5 domain or LZM restored CMTM4 function in the CMTM4 KD cells to the level of control cells (Fig. 2H) whereas deletions in the TRAF6 domain could not restore NF-κB activity in CMTM4 KD cells, indicating that the TRAF6 domain is crucial for the regulation of NF-κB activity by CMTM4 (Fig. 2H). Transfection of dominant-negative TRAF6 (Traf6 DN) completely inhibited NF-κB transcriptional activation in control (CMTM4 wild-type) tumor cells (Fig. 2H). Similarly, NF-κB activity in CMTM4 KD cells was restored by transfecting CMTM4, which was completely abolished by TRAF6 DN (Fig. 2H). These results indicate that the TRAF6 domain, but not the STAT5 or LZM domains, is required for regulation of NF-κB activity by CMTM4.

In summary, our findings showed a loss of CMTM4 inhibited tumor growth in vivo, and reduced Akt/mTOR signaling and NF-κB activation, which are dependent on the CMTM4 TRAF6 domain.

### CMTM4 regulates multiple receptor tyrosine kinases (RTKs), including EGFR

**Figure 3:**
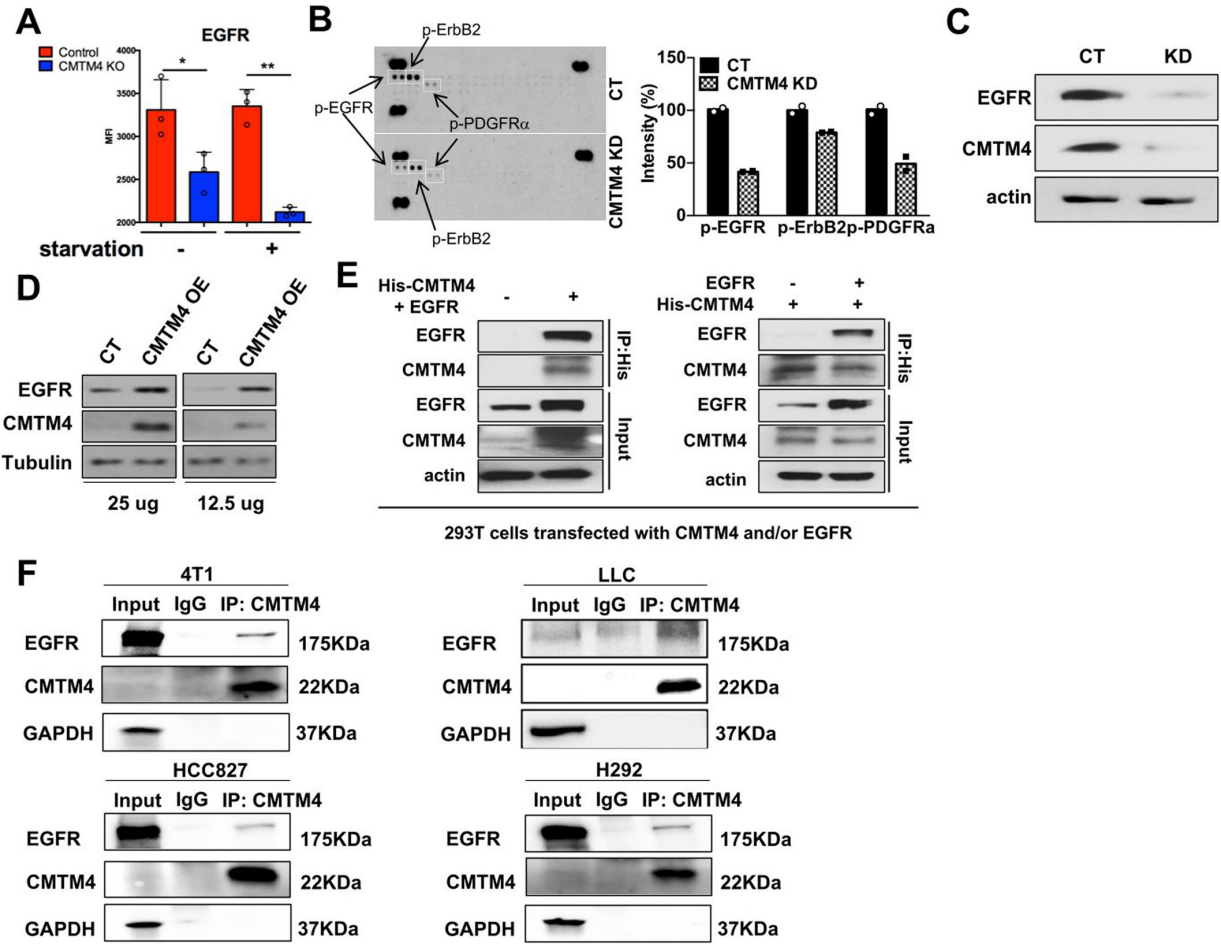
CMTM4 KD in tumor cells leads to decreased expression of receptor tyrosine kinases (RTKs). (**A**) EGFR surface expression on H292 human lung cancer cells quantified by flow cytometry. All samples were quantitated in triplicate with data representing the mean ± SD. *P* values in group without and with starvation are 0.049 and 0.0052, respectively. (**B**) The phospho-RTK array was performed using control and CMTM4 KD LLC cells and then quantified. *N* = 2. (**C**) EGFR expression in LLC cells was determined by western blot. (**D**) EGFR expression in 293T cells transfected with CMTM4 was determined by western blot. (**E**) 293T cells were transfected with both His-CMTM4 and EGFR (left panel) or with His-CMTM4 + /− EGFR (right panel). Protein extract was immuno-precipitated with anti-His antibodies, followed by western blot with antibodies against EGFR and CMTM4. (**F**) 4T1, LLC, H292, or HCC827 cell lines were starved overnight without serum. Protein extract from these cells was immuno-precipitated with anti-CMTM4 antibody, followed by western blot with antibodies against EGFR and CMTM4. *P* value calculated by Unpaired student *t* test. *P < 0.05. ***P < 0.001. Source data are available online for this figure.

Since PI3K/Akt and mTOR are activated by various RTKs (Abbosh et al, 2017; Manning and Toker, 2017), we compared RTK expression in control and CMTM4 KO cells. Human H292 lung cancer cells with CMTM4 KO showed significant decreased EGFR surface expression detected by flow cytometry (Fig. 3A). Phosphorylated RTK array indicated that multiple RTK phosphorylation was reduced by CMTM4 KD, including EGFR, ErbB2, and platelet-derived growth factor receptor alpha (PDGFRα) (Fig. 3B). EGFR is one of the RTKs that is over-activated and promotes cancer progression (Chong and Janne, 2013; Lo and Hung, 2006; Schlessinger, 2000; Wee and Wang, 2017). We confirmed that CMTM4 KD reduced EGFR in LLC lung cancer cell line (Fig. 3C; Appendix Fig. S5D). Moreover, overexpression of CMTM4 increased EGFR expression (Fig. 3D; Appendix Fig. S5E), suggesting CMTM4 upregulates EGFR expression. Since CMTM4 and EGFR are both membrane proteins, we hypothesized that CMTM4 associates with EGFR on cell membrane and regulates its expression. Indeed, immunoprecipitation from 293T co-transfected with CMTM4 and EGFR (Fig. 3E), or multiple cancer cell lines without transfection confirmed an association between CMTM4 and EGFR (Fig. 3F). Thus, our data indicate that CMTM4 is associated with EGFR and increased its surface expression, which may increase downstream Akt/mTOR signaling and NF-κB activation.

### CMTM4 controls EGFR recycling and prevents its degradation

**Figure 4:**
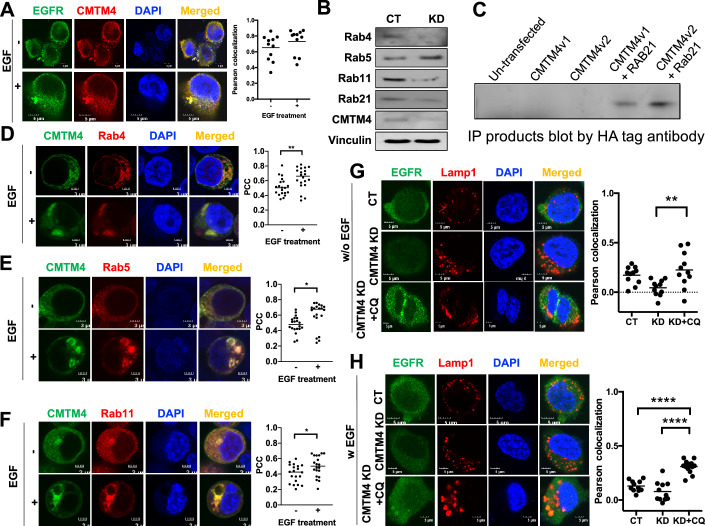
CMTM4 controls Rab expression to modulate EGFR recycling. (**A**) LLC cells were treated with 10 ng/ml EGF overnight and fixed before staining with EGFR and CMTM4. (**B**) Rab expression in control and CMTM4 KD LLC cells were determined by western blot. (**C**) Lysate from 293T cells transfected with human CMTM4-Myc with or without Rab21-HA were immuno-precipitated by Myc antibody and blotted by HA antibody. (**D**–**F**) 293T cells were transfected with CMTM4-EGFP and Rab4-mCherry (**D**), Rab5-mCherry (**E**), or Rab11-mCherry (**F**), and then treated with 10 ng/ml EGF for 1 h. *P* values are 0.0069, 0.0116 and 0.0253 for (**D**–**F**), respectively. (**G**, **H**) LLC control and CMTM4 KD cells were treated without (**G**) or with (**H**) EGF overnight and fixed before staining with EGFR and Lamp-1. *P* = 0.0061 in (**G**), and *P* < 0.0001 for both comparisons in (**H**). Representative confocal images are shown. Pearson correlation coefficient (PCC) between the GFP channel and mCherry channel was measured from 20 individual cells. *P* value calculated by Mann–Whitney test. *P < 0.05, **P < 0.01, ****P < 0.0001. Scale bars are 3 μm for (**A**, **C**, **D**) and 5 μm for (**B**, **E**, **F**). Source data are available online for this figure.

Since CMTM4 was recently found to regulate multiple surface targets post-translationally by controlling their recycling (Knizkova et al, 2022; Mezzadra et al, 2017), we reasoned that CMTM4 also affects EGFR’s endocytic and recycling pathways. By staining EGFR and CMTM4 directly in LLC cells, we found that CMTM4 co-localized with EGFR in the absence of EGF stimulation (Fig. 4A, upper panel). Upon EGF stimulation, CMTM4 and EGFR were quickly internalized and co-localized in the cytosol (Fig. 4A, lower panel), suggesting that CMTM4 may involve in EGFR recycling. Similarly, transfecting EGFR-GFP and CMTM4-mCherry to 293T cells showed co-localization between CMTM4 and EGFR (Appendix Fig. S6A).

EGFR endocytic trafficking is regulated by various Rab proteins (Barbieri et al, 2004; Ceresa, 2006; Cullis et al, 2002). We found that CMTM4 KD significantly reduced expression of Rab4/11/21 protein expression (Fig. 4B), suggesting that CMTM4 is important for EGFR endocytosis and recycling. Moreover, we found that CMTM4 associated with Rab21 by immunoprecipitation (Fig. 4C). Rab4, Rab5 and Rab21 are found on early endosomes, whereas Rab11 locates on recycling endosomes (Stenmark, 2009). Tracking CMTM4 at a sub-cellular level showed co-localization of CMTM4 with Rab4, Rab5 and Rab11 in the presence of EGF stimulation (Fig. 4D–F). This indicates CMTM4 was associated with EGFR and Rab proteins and the association might be involved in EGFR recycling.

As we showed that CMTM4 was associated and co-localized with EGFR for recycling, we reasoned that in the absence of CMTM4, EGFR is targeted for degradation in lysosome. We stained endogenous EGFR and Lamp-1 (a lysosome marker) in LLC cells and found that minimal co-localization between EGFR and Lamp-1 in control cells with or without EGF stimulation (Fig. 4G,H), suggesting that EGFR in control (i.e., CMTM4^+^) cells was rarely targeted for degradation in lysosome. Since CMTM4 KD cells has minimal EGFR expression (Figs. 3C and 4G,H) without transfecting exogenous EGFR, we found minimal co-localization between EGFR and Lamp-1 in CMTM4 KD cells as well with or without EGF stimulation (Fig. 4G,H). However, when lysosome activity was inhibited by chloroquine (CQ), we observed significant increase of co-localization between EGFR and Lamp-1 (Fig. 4G,H), suggesting that EGFR in CMTM4 KD cells was targeted for degradation in lysosome. Moreover, we found minimal co-localization of CMTM4 with other cellular compartments, including the Endoplasmic reticulum (ER) and Golgi (Appendix Fig. S6B,C), suggesting that trans-Golgi network was not involved in CMTM4’s regulation of EGFR recycling.

Similarly, transfecting EGFR-GFP and Lamp-1-mCherry to control and CMTM4 KD LLC cells showed that in the absence of EGF stimulation, EGFR has limited co-localization with lamp-1 in WT or CMTM4 KD cells (Appendix Fig. S6D), suggesting that EGFR was minimally internalized for degradation without EGF stimulation. When stimulated with EGF, EGFR was internalized but did not translocate to the lysosome in control cells (Appendix Fig. S6E). Whereas in CMTM4 KD cells, the association between EGFR with lysosome was significantly increased (Appendix Fig. S6E), indicating that EGFR was transported to lysosome for degradation in the absence of CMTM4. The result suggests that CMTM4 is involved in EGFR recycling and protects EGFR from lysosomal degradation.

In summary, CMTM4 prevents EGFR from lysosomal degradation, which further amplifies EGFR signaling and activation of the Akt/mTOR/NF-κB pathways to promote tumor growth.

### CMTM4 enhances efficacy of EGFR inhibitor to inhibit human cancer growth

**Figure 5:**
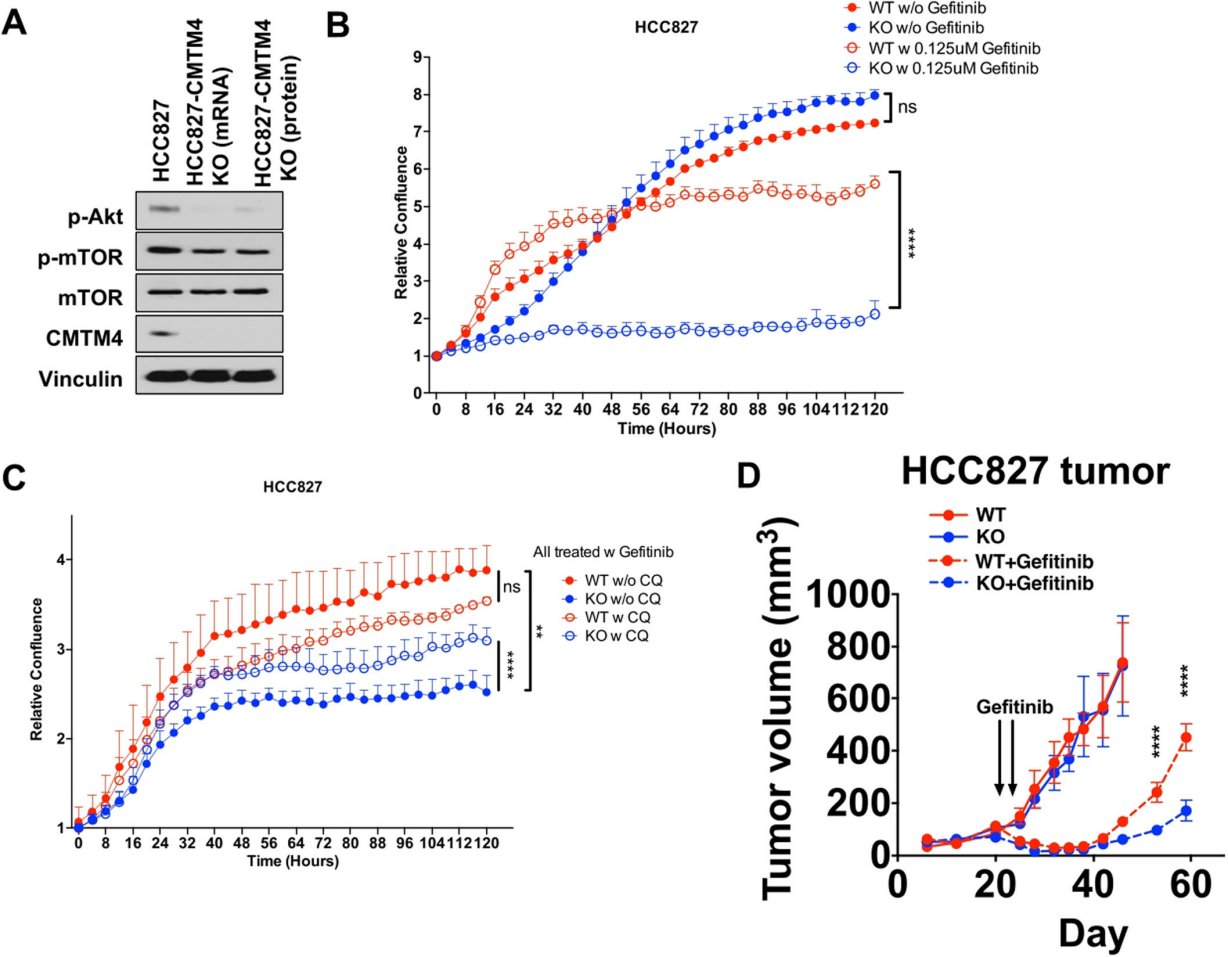
CMTM4 regulates Akt/mTOR signaling in human cancer, which is more sensitive to EGFR inhibition when CMTM4 is knockout. (**A**) EGFR mutated (over activating) cell line HCC827 was knockout of CMTM4 by CRISPR and Cas9 mRNA or protein. Akt/mTOR signaling was checked by western blot. (**B**) HCC827 control and CMTM4 KO cells were treated with or without 0.125 μM Gefitinib and tumor cell growth was measured by Incucyte. *n* = 5. Data representing the mean + SD. Two-way ANOVA test. *P* value between WT and KO with genfitinib treatment is < 0.0001. (**C**) HCC827 control and CMTM4 KO cells were all treated with 0.125 μM Gefitinib, with or without 10 μM CQ. Tumor cell growth was measured by Incucyte. *n* = 5. Data representing the mean + SD. Two-way ANOVA test. *P* value between KO without CQ and KO with CQ is <0.0001. *P* value between WT without CQ and KO without CQ is 0.0009. (**D**) HCC827 control and CMTM4 KO tumors were implanted subcutaneously to NSG mice (*n* = 7) and treated with or without 150 mg/kg Gefitinib at Day 21 and Day 24. Data representing the mean ± SD. Two-way ANOVA test. *P* value between WT and KO with genfitinib treatment is <0.0001 at Day 53 and Day 59. *P* values at the last two time-points are <0.0001. **P < 0.01. ****P < 0.0001. Experiments were repeated twice with similar result. Source data are available online for this figure.

To confirm our findings in the human system, we used the human lung cancer cell line HCC827 with exon 19 deletion in EGFR, which is an activating mutation that results in excess EGFR expression (Lee et al, 2018; Robichaux et al, 2021). We confirmed CMTM4 expression in this cell and generated CRISPR knockout lines using Cas9 mRNA or protein (Fig. 5A; Appendix Fig. S7A). Loss of CMTM4 significantly reduced pAKT/mTOR signaling (Fig. 5A; Appendix Fig. S7A), which is consistent with our findings in murine tumor cell lines (Fig. 2D–G).

Since we found that CMTM4 increases EGFR recycling, we evaluated the effect of the EGFR tyrosine kinase inhibitor, gefitinib, on control and CMTM4 KO cells. HCC827 cells with CMTM4 KO grew at a rate similar to its parental control without gefitinib treatment in vitro (Fig. 5B; Appendix Fig. S7B,C). However, CMTM4 KO cells show elevated sensitivity to gefitinib treatment (Fig. 5B; Appendix Fig. S7B,C), indicating CMTM4 plays a role in tumor intrinsic resistance to EGFR tyrosine kinase inhibitors. Our data suggests CMTM4 is a novel regulator of the EGFR/AKT/mTOR pathway in human tumor and affects sensitivity to EGFR tyrosine kinase inhibition. Moreover, as we demonstrated EGFR is translocated to lysosome for degradation after EGF stimulation (Fig. 4G,H), we reasoned that inhibiting lysosomal activity in CMTM4 KO cells can increase resistance against gefitinib treatment. Indeed, CMTM4 KO cells treated with chloroquine (CQ) were more resistant against gefitinib (Fig. 5C; Appendix Fig. S7D). However, control cells treated with and without chloroquine (CQ) had similar sensitivity to gefitinib (Fig. 5C; Appendix Fig. S7D), since EGFR translocation to lysosome in CMTM4 KD cells was increased when compared to control cells after EGF stimulation (Fig. 4G,H; Appendix Fig. S6D,E). Interestingly, although CQ treatment increased cell viability in CMTM4 KO cells, CQ treatment alone decreased cell viability in both control and CMTM4 KO cells (Appendix Fig. S7D), indicating lysosome inhibition alone can disrupt protein degradation and decrease cell viability.

To mimic the natural development of EGFR inhibitor resistance in patients, we implanted the HCC827 control and CMTM4 KO cancer cells in animals and treated with gefitinib. Since HCC827 tumor is sensitive to EGFR inhibitors, both control and CMTM4 KO tumor growth were significantly inhibited after gefitinib treatment (Fig. 5D). However, the tumors recurred after removal of gefitinib treatment and interestingly, the control tumor grew significantly larger than the CMTM4 KO tumor (Fig. 5D). This result indicated that loss of CMTM4 can increase the drug sensitivity and reduce tumor resistance to EGFR inhibition.

### EGF stimulation promotes an immune suppressive tumor microenvironment, which is inhibited by the loss of CMTM4

Since we found significant reduced tumor growth with CMTM4 KD/KO cancer cells in vivo (Fig. 2A–C) but not in vitro (Appendix Fig. S4C), we determined whether the immune response, especially the adaptive immunity, is required for the retarded tumor growth. We implanted control and CMTM4 KD LLC tumor cells to immuno-deficient mice and we found no significant growth difference between control and CMTM4 KD tumors (Appendix Fig. S8A). Similar observation was found with control and CMTM4 KO H292 tumor cells implanted to immuno-deficient animals (Appendix Fig. S8B). These results indicate that adaptive immunity is involved in suppressing the growth of CMTM4 KD/KO tumors.

**Figure 6:**
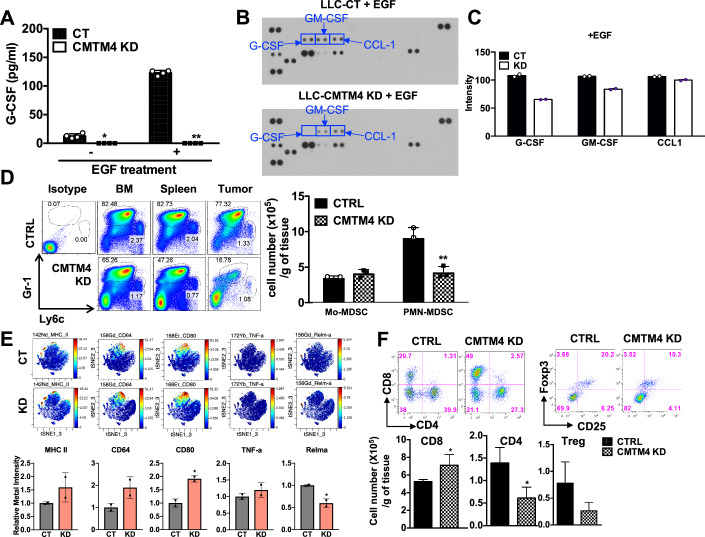
CMTM4 regulates inflammatory mediators by activating EGFR signaling, which recruits immunosuppressive MDSCs. (**A**) LLC control and CMTM4 KD cells were treated with 10 ng/ml EGF for 2 days. G-CSF production was measured by ELISA. All samples were quantitated in duplicate with data representing the mean ± SD. *n* = 4. *P* values are 0.001 and <0.0001 without and with EGF stimulation, respectively. (**B**, **C**) LLC control and CMTM4 KD cells were cultured in the presence of 10 ng/ml EGF for 48 h. Cytokine arrays (**B**) were performed with supernatants from control and CMTM4 KD LLC cell cultures and results were quantified (**C**). *n* = 2. (**D**–**F**) Control or CMTM4 KD LLC cells were subcutaneously inoculated into C57BL/6 mice (*n* = 3). Once tumors reached greater than 1 × 1 cm^2^, mice with similarly sized tumors were sacrificed and bone marrow, spleen, and tumor tissues were harvested. (**D**) Single-cell suspensions were prepared from bone marrow, spleen, and tumor tissues and subjected to FACS analysis. Cell suspensions were gated on CD45^+^CD11b^+^ cells. The number of MDSCs from each tumor was determined in the right panel. Samples were quantitated in triplicate with data representing the mean ± SD. *P* value is 0.0075 for PMN-MDSC. (**E**) Expression of M1-like markers (MHC II, CD64, CD80, TNF-α) and M2-like marker (Relm-α) on TILs from LLC control and CMTM4 KD tumors displayed on t-SNE map by CyTOF analysis (upper panel). Mean metal intensity of these markers were quantified in lower panel. Samples were quantitated in triplicate with data representing the mean ± SD. *P* values are 0.022 and 0.030 for CD80 and Relm-α. (**F**) Tumor-infiltrating T cell populations were assessed by FACS analysis (upper) and the number of T cells in the tumor tissues was calculated (lower). Samples were quantitated in triplicate with data representing the mean ± SD. *P* = 0.0465 for CD8 T cells, *P* = 0.0495 for CD4 T cells, *P* = 0.0819 for Treg cells. *P* value calculated by unpaired Student *t* test. *P < 0.05, **P < 0.01. The data shown are representative of three reproducible experiments. Source data are available online for this figure.

Since the adaptive immune response is required for the reduced tumor growth with CMTM4 KD/KO cancer cells, we hypothesized that cytokine profile differences between control and CMTM4 KD/KO cells may contribute to different immune responses. CMTM4 regulates RTK function and further controls downstream signal activation, including NF-κB and Akt/mTOR pathways (Fig. 2 and 3), which may induce tumor productions of various cytokines and chemokines in cancer cells, including CCL-1, IL-1β, G-CSF (Tang et al, 2018; Taniguchi and Karin, 2018; Welte et al, 2016). Since the loss of CMTM4 reduced EGFR expression, we reasoned that the cytokine profile may be affected by CMTM4 KD. We stimulated control and CMTM4 KD cells with EGF and found that the control cancer cells secreted a high level of G-CSF, while CMTM4 KD cells significantly reduced G-CSF production (Fig. 6A). We further performed cytokine array analyses with tumor cells stimulated with EGF and found increased protein levels of G-CSF, GM-CSF, and CCL-1 in control cells compared to CMTM4 KD cells (Fig. 6B,C). The results suggest that EGFR levels may contribute to different cytokine profiles between control and CMTM4 KD cells.

Since the production of cytokines/chemokines by tumor is essential to induce a suppressive tumor immune microenvironment, we hypothesized that different cytokines/chemokines profiles by CMTM4 KD will result in different immune environments. For example, G-CSF has been shown to promote granulopoiesis and PMN-MDSC accumulation (Eyles et al, 2006; Semerad et al, 2002), and CCL1/2 is known to recruit monocytes. Indeed, we found significant reductions of PMN (Gr-1^High^Ly6C^Low^) MDSCs in the tumors, bone marrow and spleen, from CMTM4 KD tumor-bearing mice when compared to mice bearing control tumor with a similar tumor size (Fig. 6D; Appendix Fig. S9A). However, no substantial differences in monocytic (Gr-1^Low^Ly6C^High^) MDSCs were observed between CMTM4 KD and control tumor. Similar results were observed in the mice bearing hepatic metastatic colon cancer (MCA26) (Appendix Fig. S9B,C).

We further evaluated whether the reduction of MDSCs in the tumor is attributed to impaired MDSC recruitment. We established a hepatic metastasis of lung tumor model by intra-hepatically implanting control and CMTM4 KD LLC cells, considering liver is one of the most common metastatic sites for lung cancer (Milovanovic et al, 2017; Riihimaki et al, 2014). Sorted MDSCs from CD45.1 tumor-bearing mice were adoptively transferred into control or CMTM4 KD LLC tumor-bearing MaFIA (macrophage Fas-induced apoptosis) mice (CD45.2) that had been depleted of CD115^+^ cells. Three days after adoptive transfer, significantly lower numbers of PMN-MDSCs were present in CMTM4 KD tumors whereas the number of tumor-infiltrating monocytic MDSCs was not significantly altered (Appendix Fig. S10A). Interestingly, iNOS^+^ MDSC infiltration was increased and Arg1^+^ MDSC infiltration was reduced in CMTM4 KD tumors (Appendix Fig. S10B). These results suggest that the CMTM4 expressed within tumor cells increases G-SCF secretion and infiltration of PMN-MDSCs in the tumor microenvironment.

Since MDSCs have been shown to accumulate in hosts with advanced malignancies, suppress the antitumor immune response, and promote tumor progression (Almand et al, 2001; Ochoa et al, 2007; Ozao-Choy et al, 2009; Pan et al, 2008; Pan et al, 2008; Yang et al, 2013), we determined whether the function of MDSCs was altered due to CMTM4 KD, in addition to the immune composition being changed within the tumor environment. CyTOF analysis showed loss of CMTM4 reduced expression of M2-like marker RELMα in myeloid cells, whereas M1-like markers, including MHC II, CD64, CD80, and TNF-α were increased in myeloid cells, indicating a phenotypic shift from M2-like MDSCs to M1-like MDSCs (Fig. 6E). Furthermore, MDSCs from CMTM4 KD tumors showed significantly higher levels of iNOS expression (M1 phenotypic marker) with simultaneous decreased expression of arginase 1 (M2 phenotypic marker) (Appendix Fig. S10C). Functionally, monocytic MDSCs from CMTM4 KD tumor-bearing mice showed significantly impaired suppressive activity toward OT-II T-cell proliferation (Appendix Fig. S10D). Our findings showed that targeting CMTM4 not only reduced recruitment of MDSCs but also altered the phenotype of MDSCs.

MDSCs play a prominent role in spurring angiogenesis within tumors (Yang et al, 2004). We confirmed that knockdown of CMTM4 resulted in reduced angiogenesis in tumor tissues (Appendix Fig. S10E). Consistent with our previous publication demonstrating the role of MDSCs in T_reg_ cell expansion in tumor-bearing mice (Huang et al, 2006a; Pan et al, 2010), we observed that T_reg_ cells and the CD4 population were significantly reduced in CMTM4 KD tumors when compared to mice bearing control wild-type tumors (Fig. 6F). In contrast, CD8 cells were significantly increased in CMTM4 KD tumors (Fig. 6F), suggesting an important role of T cells contributing to the reduced growth of CMTM4 KD/KO tumor (Fig. 2A–C). Indeed, we previously showed LLC and H292 control and CMTM4 KD tumor implanted in immuno-deficient mice grew in a similar rate in vivo (Appendix Fig. S8A,B), which demonstrated the importance of adaptive immunity, including T cells, in controlling CMTM4 KD tumor growth.

In summary, our data suggest that CMTM4 contributes to EGFR degradation and regulates its downstream mTOR/Akt/NF-κB signaling, which may regulate the production of inflammatory cytokines/chemokines (e.g., G-CSF) to recruit MDSCs and promote their immunosuppressive function in the tumor microenvironment.

### Targeting CMTM4 reduced tumor growth in vivo, promoted animal survival, and synergized with immune checkpoint blockade

**Figure 7:**
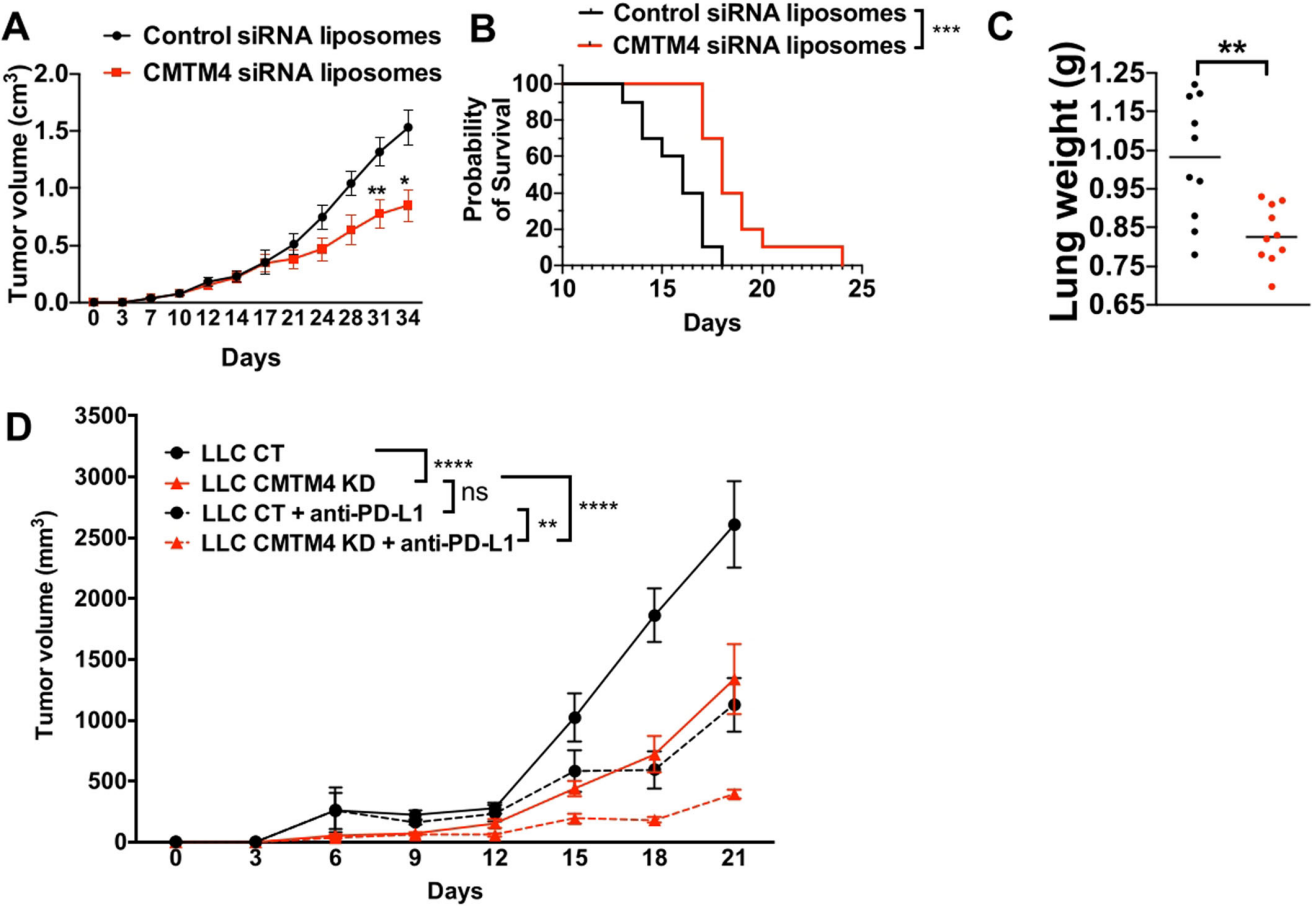
Targeting CMTM4 inhibited tumor growth and metastasis, promoted animal survival and synergized with immune checkpoint blockade. (**A**) Tumor cells were subcutaneously inoculated into flank of mice (*n* = 10) and control or CMTM4 siRNA liposomes were injected intratumorally every 3 days starting at day 7. Tumor growth was measured every 3–4 days. Samples were quantitated with data representing the mean ± SD. Two-way ANOVA test. *P* = 0.0095 at Day 31 and *P* = 0.015 at Day 34. (**B**, **C**) Tumor cells were intravenously injected into mice (*n* = 10) and control or CMTM4 siRNA liposomes were injected intravenously every 3 days starting at day 7. Data representing the mean ± SD. (**B**) Animal survival was plotted as Kaplan–Meier curves. Log-rank test. *P* = 0.0008. (**C**) Lung weight was measured from animals that received control or CMTM4 siRNA liposomes. Unpaired *t* test. *P* = 0.0027. (**D**) LLC control and CMTM4 KD tumors were implanted subcutaneously in C57BL/6 mice (*n* = 7). 200 ug IgG control or PD-L1 antibody was injected into the animals every 3 days. P = 0.029 for LLC control and CMTM4 KD tumors treated with PD-L1. *P* < 0.0001 for both comparisons between LLC control and CMTM4 KD tumors without PD-L1 treatment, and between LLC CMTM4 KD tumors with and without PD-L1 treatment. Data representing the mean ± SD. Two-way ANOVA test. *P* < 0.0001. *P < 0.05. **P < 0.01. ***P < 0.01. ****P < 0.0001. Experiments were repeated twice with similar results. Source data are available online for this figure.

To explore more therapeutic potential of targeting CMTM4 in vivo for clinical application, we developed CMTM4 targeting siRNA encapsulated in liposomes. In mice bearing subcutaneous LLC control tumor, intra-tumoral injection of CMTM4 siRNA-liposomes significantly inhibited subcutaneous tumor growth (Fig. 7A). In mice with lung metastatic LLC control tumor, systematic delivery of CMTM4 siRNA-liposomes improved animal survival (Fig. 7B), and reduced lung metastasis (Fig. 7C). The results showed the efficacy of CMTM4 inhibition in suppressing tumor growth and metastasis and demonstrated the potential of inhibiting CMTM4 as a novel cancer treatment.

Since loss of CMTM4 reversed the suppressive tumor immune environment by reducing PMN-MDSC infiltration (Fig. 6D–F), we reasoned that immune checkpoint blockade, such as PD-L1 antibody treatment, can further promote T cell activation in a less suppressive environment when combined with CMTM4 KD, and eventually inhibit tumor growth. Animals bearing control or CMTM4 KD LLC tumor were treated with anti-PD-L1 antibody. We found reduced tumor growth with either CMTM4 KD or anti-PD-L1 antibody treatment alone (Fig. 7D). Interestingly, the combination of CMTM4 KD and anti-PD-L1 antibody treatment further inhibited tumor growth in vivo (Fig. 7D). This indicated that CMTM4 inhibition can serve as an adjunctive therapy in combination with other therapies such as EGFR inhibitor (Fig. 5B–D), immune checkpoint blockade (Fig. 7D) and potentially T cell therapy.

## Discussion

The complex interactions between tumor cells and the microenvironment pose a major obstacle in the development of effective anti-tumor therapies. Counteracting or neutralizing pro-tumor inflammation may result in a switch to an immune conducive tumor microenvironment (Coussens et al, 2013). Alternatively, targeting immune suppressive cells can quell a cascade of tumor-promoting events, thereby ultimately favoring cancer eradication. Although CMTM4 was shown to be highly expressed in various cancer types (Ozao-Choy et al, 2009), its functional roles in tumor progression and establishment of the tumor microenvironment have not been fully investigated. In this study, we showed that CMTM4 regulated tumor growth by modulating tumor-associated inflammation and leukocyte infiltration related to EGFR expression, trafficking, and activation. Human carcinoma tissues and murine tumor cell lines expressed high levels of CMTM4 (Fig. 1A–D,G). Interestingly, the cancer patient tissues at a more advanced stage had higher CMTM4 expression than those at an earlier stage in lung adenocarcinoma (Fig. 1C). Similarly, triple-negative basal breast cancer cell lines had higher CMTM4 expression when compared to luminal type cell lines (Fig. 1D), suggesting that CMTM4 is associated with an aggressive phenotype of cancer. We further demonstrated that CMTM4 could be used as an independent prognostic factor for poor survival in patients with certain cancer types since the corresponding patient group exhibiting higher expression of CMTM4 had a significantly reduced survival rate when compared to the same cancer type with lower CMTM4 expression (Fig. 1E,F; Appendix Table S1).

The role of CMTM4 in cancer development has not been fully understood. Ting Li et al, showed that CMTM4 inhibited tumor proliferation and functioned as a tumor suppressor gene, and its expression was downregulated in certain types of tumor (Li et al, 2015a). However, the study used an adenovirus overexpression system in immuno-deficient mice. Other cancer types, including breast cancer, lung adenocarcinoma, lung squamous cell carcinoma, melanoma, ovarian, pancreatic, and prostate cancers, had higher CMTM4 expression levels, which is consistent with our data (Fig. 1A,B). Taken together, these studies suggest that CMTM4 may have different roles depending on the cancer type and the role of CMTM4 needs to be delineated in a cancer type specific manner. In contrast to a previous finding that CMTM4 inhibited HeLa cell growth by inducing G2/M phase arrest (Plate et al, 2010a), we found that CMTM4 knockdown did not affect the proliferation of tumor cells in vitro (Appendix Fig. S4C). Moreover, expression of CMTM4 is not suppressed in carcinoma tissues, even though CMTM4 is closely associated with the tumor suppressor locus 16q22.1, which is frequently deleted in multiple tumors. By contrast, CMTM3, which is located in the same region, is silenced in carcinomas and recognized as a tumor suppressive gene (Ozao-Choy et al, 2009). Recently, CMTM6 and CMTM4 were identified as oncogenic factors to promote PD-L1 expression on tumor cells (Burr et al, 2017; Mezzadra et al, 2017). Knockout of CMTM6 showed significant decrease of PD-L1, whereas CMTM4 serves as a backup for CMTM6 (Burr et al, 2017; Mezzadra et al, 2017). Moreover, CMTM4, but not CMTM6, was found interacting with IL-17 receptor C in stromal cells, to mediate autoimmune pathology (Knizkova et al, 2022). In our study, we found that CMTM4 can modulate the tumor immune microenvironment by promoting EGFR recycling to increase G-CSF and CCL-1 production, which recruit neutrophils and MDSC to induce an immune suppressive tumor microenvironment. Therefore, unlike other CMTM family members, CMTM4 might not have a tumor suppressor role in certain cancers. Instead, CMTM4 can be a prognostic marker for multiple cancer types (Fig. 1) and a predictive marker for breast and lung cancer patient survival (Fig. 1E,F). Moreover, we found that CMTM4 is significantly associated with epithelial-mesenchymal transition (EMT) gene signatures, suggesting that CMTM4 can be a biomarker for tumor metastasis. Finally, given the reports about decreased tumor PD-L1 expression with CMTM4 deficiency (Burr et al, 2017; Mezzadra et al, 2017), and our result that CMTM4 KD tumor was more sensitive to PD-L1 blockade (Fig. 7D), CMTM4 can also be a predictive biomarker of response to immune checkpoint therapy.

During tumorigenesis, the tumor microenvironment is gradually altered in favor of tumor growth. Several mediators, such as cytokines and chemokines secreted by cancer cells, recruit immune cells to the tumor to promote tumor growth. Therefore, immune recruitment by cancer cells is a crucial step during oncogenesis and is a hallmark of cancer. We found that inhibition of CMTM4 in cancer cells reduced tumor growth in vivo (Fig. 2A–C) but not in vitro (Appendix Fig. S4C), which suggests that CMTM4 is an important regulator in the establishment of the suppressive and pro-tumor microenvironment. Our data indicate that CMTM4 KD decreased EGFR signaling, resulting in reduced inflammatory cytokines (including G-CSF) production that recruit major immunosuppressive cells, such as PMN-MDSCs (Fig. 6D; Appendix Fig. S9A) and T_reg_ cells (Fig. 6F), into tumors. Furthermore, MDSCs showed increased M1-like anti-tumor functional phenotype and reduced M2-like pro-tumor phenotype in CMTM4 KO tumor-bearing mice (Fig. 6E; Appendix Fig. S10B–D). Reducing the immunosuppressive effects of immune cells has emerged as a promising approach to improve the efficacy of anti-tumor therapeutics. However, direct targeting of MDSCs in cancer patients presents several limitations due to the diverse phenotypes and lack of specific surface markers of MDSCs. Therefore, CMTM4 represents a novel target for reversing the tumor-promoting phenotype of MDSCs without directly eliminating these cells. Moreover, since we found reduced PMN-MDSCs in the tumor environment when CMTM4 was deficient in cancer cells, we hypothesized that immune checkpoint blockade is more effective in restoring the T cell function since these immunosuppressive cells are the major contributors to T cell exhaustion. Indeed, we found that CMTM4 KD tumor was more responsive to PD-L1 antibodies treatment (Fig. 7D), which is resulted from reduced inflammatory cytokines productions from CMTM4 KD cells (Fig. 6) with retarded EGFR signaling (Fig. 3).

Our results further indicate that CMTM4 controls oncogenic pathways, RTKs, to alter the tumor immune microenvironment (Figs. 2 and 6). RTKs have been identified as the most frequent oncogenic driver involved in cancer cell proliferation, survival, and metastasis (Casaletto and McClatchey, 2012). Targeting RTK can inhibit tumor growth and be synergistic with other anti-cancer therapeutics. CMTM3 has been reported defective in gastric cancer, and overexpression of CMTM3 inhibited cell migration mediated by EGF signaling (Wang et al, 2009; Yuan et al, 2017). As mechanism, CMTM3 promoted early endosome fusion by activating Rab5 to downregulate EGFR on cell surface (Wang et al, 2009; Yuan et al, 2017). CMTM5’s expression is negatively correlated with PI3K/AKT pathway activation in liver cancer and prostate cancer (Yuan et al, 2020). CMTM5-v1 increased EGFR inhibitor’s sensitivity against prostate cancer cells (Yuan et al, 2020). Similar with CMTM3, CMTM7 enhanced Rab5 activation to attenuate EGF signaling in esophageal cancer (Huang et al, 2019). Rab5 activation induced endocytosis and decreased EGFR recycling, thus, reducing EGFR surface level and downstream PI3K/AKT activation (Huang et al, 2019). In our study, CMTM4 KD decreased the expression and activation of multiple RTKs (Fig. 3B–D). CMTM4 KD reduced activation of EGFR/Akt/mTOR and NF-κB pathways (Fig. 2D,E), suggesting that different CMTM family members may have distinctly different functions in cancer cells. Our findings suggest that targeting CMTM4 may inhibit RTKs, including EGFR. We also found that CMTM4 KD diminished the expression of multiple cytokines and chemokines responsible for MDSC recruitment and accumulation. Notably, G-CSF production was significantly diminished in response to EGF in CMTM4 KD cells (Fig. 6A), consistent with the finding that the oncogenic RTK-mTOR pathway drives MDSC accumulation through G-CSF (Welte et al, 2016). Our data demonstrate that CMTM4 controls tumor cell-intrinsic oncogenic pathways that determine the tumor’s ability to recruit MDSC.

Mitogen-inducible gene 6 (MIG6) has been studied intensively for its role in regulating EGFR endocytosis and recycling (Chen et al, 2024; Maity et al, 2015). MIG6 inhibits EGFR kinase activity by facilitating the degradation of wild-type EGFR (Maity et al, 2015). Interestingly, tumor cells with mutant EGFR exhibited enhanced tyrosine phosphorylation of MIG6 at Y394/Y395, resulting in increased binding between mutant EGFR and MIG6. Surprisingly, the increased binding does not direct mutant EGFR to lysosome for degradation, in contrast to wild-type EGFR (Maity et al, 2015). Another study reported that targeted therapies including specific inhibitors targeting KRAS G12C mutation induced MIG6 loss in different lung cancer cell lines (Chen et al, 2024). Similarly, EGFR tyrosine kinase inhibitor (erlotinib) treated lung cancer cells downregulated miR21, which regulates the TNF and NF-κB signaling in mutant EGFR cells (Gong et al, 2018). These studies suggest a potential contribution of CMTM4 in this process when cells were treated with gefitinib or specific KRAS G12C inhibitors. We treated LLC cells and HCC827 cells with gefitinib and a KRAS G12C mutation inhibitor, adagrasib and determined whether the CMTM4 level was altered. Interestingly, we found adagrasib treatment reduced CMTM4 expression in LLC cells but not in HCC827, considering that LLC cells harbor the KRAS G12C mutation. On the other hand, gefitinib treatment didn’t alter the expression of CMTM4 in both LLC and HCC827 cells. The role of CMTM4 in MIG6-mediated EGFR degradation in lysosomes will need to be further investigated in future studies.

We further showed that CMTM4 was associated with Rab GTPases that were involved in EGFR endocytosis and recycling (Fig. 4). CMTM4 KD suppressed expression of Rab4/11/21 (Fig. 4B), which may contribute to EGFR endocytosis and recycling (Fig. 4G,H; Appendix Fig. S6D,E). Rab4 and Rab21, which are involved in recycling, were significantly diminished in CMTM4 KD cells (Fig. 4B). Taken together, our data suggest that CMTM4 is associated with Rab4 and Rab21 to regulate EGFR recycling to the membrane. Interestingly, increased co-localization of EGFR with Lamp1 in response to EGF stimulation in CMTM4 KD cells when treated with lysosome inhibitor chloroquine, compared to control cells (Fig. 4G,H; Appendix Fig. S6D,E). This indicates reduced EGFR recycling back to cell surface and increased trafficking towards lysosome for degradation in CMTM4 KD cells (Fig. 4G,H; Appendix Fig. S6D,E). Several Rab proteins are considered to be predictive markers for successful cancer treatment and potential candidate targets for enhancing therapeutic efficacy (Zhang et al, 2020). Diverse studies indicate that CMTM family members co-localize with Rab proteins and regulate Rab protein functions. CMTM4 controls VE-cadherin internalization by co-localizing with Rab4 and Rab7, which are markers of endocytic trafficking (Chrifi et al, 2019). CMTM3 reduces EGFR expression and cell migration by enhancing Rab5 activation (Yuan et al, 2017). CMTM6 co-localizes with Rab11 and is involved in the endocytic recycling of PD-L1 (Burr et al, 2017). Although each member of the CMTM family has its own distinct function associated with various endocytic trafficking pathways, their specific roles in this process needs to be further investigated. Thus, targeting CMTM members to control endocytosis and recycling represents a novel therapeutic strategy for various diseases.

In summary, we have identified a novel regulatory role of CMTM4 in activating multiple RTKs including EGFR, and downstream pathways including mTOR and PI3K/Akt signaling. CMTM4 KO enhances EGFR degradation and inhibits its recycling post-translationally. As a result, CMTM4 KO tumor cells have increased sensitivity to EGFR inhibitor. Inhibition of CMTM4 promoted efficacy of EGFR inhibitor to reduce tumor growth and prolong animal survival. Importantly, the activation of EGF signaling controlled by CMTM4 is responsible for the production of multiple inflammatory cytokines and chemokines, including G-CSF. Therefore, loss of CMTM4 in tumor cells decreased EGFR signaling, and hence, decreased G-CSF production and PMN-MDSCs recruitment, which led to a less suppressive tumor immune microenvironment and reduced tumor growth. Therapeutically, targeting CMTM4 with siRNA-liposomes reduced tumor growth in vivo and prolonged animal survival. Moreover, the combination of CMTM4 with immune checkpoint blockade significantly inhibited tumor growth compared to single treatment alone. Our findings suggest that CMTM4 is a novel intrinsic molecule that can be targeted to reverse the immune-suppressive tumor microenvironment and improve efficacy of chemotherapy and immunotherapies.

## Methods

**Table:** Reagents and tools table

Reagent/resource	Reference or source	Identifier or catalog number
**Experimental models**		
C57BL/6 mice	Jackson Laboratories	
BALB/c mice	Jackson Laboratories	
MaFIA mice	Dr. Don A. Cohen	
Primary human carcinoma samples	Biorepository Core, Icahn School of Medicine at Mount Sinai	
4T1 cells	ATCC	1. CRL-2539
HEK-293 cells	ATCC	CRL-1573
B16-F10 cells	ATCC	CRL-6475
LLC cells	ATCC	CRL-1642
HCC827 cells	ATCC	CRL-2868
H292 cells	ATCC	CRL-1848
**Recombinant DNA**		
pSIREN-RetroQ	Clontech Laboratories Inc.	632455
pCDNA3.1	Addgene	128744
pCDNA3.1-CMTM4 wo STAT5 BD	This study	
pCDNA3.1-CMTM4 wo LZM BD	This study	
pCDNA3.1-CMTM4 wo LZM&TRAF6 BD	This study	
pCDNA3.1-CMTM4	This study	
mRuby2-Rab5a-7	Addgene	55911
mIFP12-Rab4a-7	Addgene	56261
mCherry-Rab11a-7	Addgene	55124
pSR si-CMTM4	This study	
**Antibodies**		
p-NF-κBp65	Cell Signaling Technology	3033S
NF-κBp65	Cell Signaling Technology	8242S
EGFR	Cell Signaling Technology	4267S
PTEN	Cell Signaling Technology	9559S
Akt	Cell Signaling Technology	4691S
p-Akt	Cell Signaling Technology	4060S
mTOR	Cell Signaling Technology	2983S
p-mTOR	Cell Signaling Technology	5536S
Rab4	Cell Signaling Technology	2167S
Rab5a	Cell Signaling Technology	3547S
Rab11	Cell Signaling Technology	5589S
Tubulin	Cell Signaling Technology	2144S
Actin	Cell Signaling Technology	4967S
Vinculin	Cell Signaling Technology	4650S
GAPDH	Cell Signaling Technology	2118S
CMTM4 (western blot)	Cell Signaling Technology	17433S
CMTM4 (immunohistochemistry)	Atlas Antibodies	HPA023890
Rab21	Santa Cruz Biotechnology	81917
Anti-rabbit HRP antibody	Cell Signaling Technology	7074P2
Anti-mouse HRP antibody	Cell Signaling Technology	7076P2
Anti-rabbit IgG antibody	Vector Laboratories	TI-1000-1.5
Anti-Ly6C flow antibody	Biolegend	128008
Anti-Ly6G flow antibody	eBioscience (ThermoFisher)	46-9668-82
Anti-Gr-1 flow antibody	Biolegend	108410
Anti-CD11b flow antibody	Biolegend	101206
Anti-CD45 flow antibody	Biolegend	103116
Anti-CD206 flow antibody	Biolegend	141708
Anti-iNOS flow antibody	eBioscience (ThermoFisher)	25-5920-82
Isotype-matched mAbs	Biolegend	N/A
Anti-Arginase flow antibody	R&D Systems	IC5868A
**Oligonucleotides and other sequence-based reagents**		
CMTM4-specific targeted shRNA	IDT	N/A
**Chemicals, enzymes and other reagents**		
CMTM4 knockout kit v2	Synthego	N/A
Mouse cytokine array kit	R&D Systems	ARY006
Mouse phospho-receptor tyrosine kinase array kit	R&D Systems	ARY014
Dynabeads His-Tag isolation and pulldown kit	Life Technologies	10103D
Protein A/G magnetic beads	Pierce^TM^	88802
Dual-Glo^R^ Luciferase Assay	Promega	E2920
Percoll density gradient	GE Healthcare	17089101
OVA peptides	AnaSpec	AS-27024
Trizol	Invitrogen	15596026
M-MLV reverse transcriptase	Promega	M1701
FastStart SYBR Green Master Mix	Roche	4673484001
G-CSF ELISA kit	Life Technologies	EMCSF3
**Software**		
Flowjo	Flowjo	
GraphPad Prism 9	GraphPad	
**Other**		
FACS Canto II	BD Biosciences	
FACS LSRFortessa	BD Biosciences	
confocal scanning microscope	Zeiss	
FluoView TM3000	Olympus	
MoFlo XPD High-Speed Cell Sorter	Beckman Coulter	
ViiA™ 7 RT-PCR system	Applied Biosystems	

### Methods and protocols

#### Experimental animals

MaFIA mice (Burnett et al, 2004) were a gift from Dr. Don A. Cohen (University of Kentucky, Lexington, KY). Animal experiments were performed following the IACUC guidelines of the Houston Methodist Research Institute with approved protocol IS00007478.

#### Immunohistochemistry and immunostaining

Primary human carcinoma samples were obtained from the Biorepository Core, Icahn School of Medicine at Mount Sinai. Paraffin-embedded sections were stained with polyclonal antibodies against CMTM4. The endothelial cell layer of murine tumors was stained using rabbit anti-mouse CD31 followed by incubation with Texas Red conjugated goat anti-rabbit IgG antibody. Slides were examined and imaged using a confocal scanning microscope (Zeiss) or FluoView TM3000 at Houston Methodist Research Institute’s Advanced Cellular and Tissue Microscopy Core Facility.

#### Generation of KO or KD cells

CRISPR-cas9 knockout (KO) or shRNA knockdown (KD) of CMTM4 were performed in multiple cell lines. For 4T1 and H292, CRISPR-cas9 knockout (KO) was performed using Cas9 virus to generate stable cell lines, which were transduced with synthesized gRNA and selected for single colony culture. CMTM4 expression was confirmed by western blot and the colonies with lost expression of CMTM4 were picked for further experiments. For LLC and HCC827 cells, Synthego Gene knockout kit v2 was used to generate KO cells, with gRNA and Cas9 protein or Cas9 mRNA. shRNA-mediated gene silencing was performed using the retroviral expression vector pSIREN-RetroQ to express small hairpin RNA (shRNA) in LLC and B16 cells. The CMTM4-specific insert consisted of a 19-nt sequence (CTTGATTAGAAGGACGGTT) at the target of 3’ UTR, separated by a non-complementary spacer from the reverse complement of the same 19-nt sequence to form the shRNA duplex, referred to as pSR si-CMTM4. A control vector was used as a control. Transfected cells were selected with antibiotics for 2 weeks until stable cell lines were generated before any downstream experiments.

#### Western blot, co-immunoprecipitation (co-IP), cytokine array, and proteome profiler RTK array

Protein samples from cells were separated using sodium dodecyl sulfate (SDS)-polyacrylamide gels and then transferred to PVDF membranes. The membranes were blocked using a 4% skim milk solution, then incubated with the appropriate antibody, followed by a secondary antibody conjugated with horseradish peroxidase. The immuno-reactive bands were visualized using the ECL system (Thermo Scientific). Control and CMTM4 KD cell supernatants were analyzed for cytokine array using the proteome profiler mouse cytokine array kit following the manufacturer’s instructions. Control and CMTM4 KD cell lysates were analyzed for proteomic profile using the proteome profiler mouse phospho-receptor tyrosine kinase array kit following the manufacturer’s instructions. For immunoprecipitation, the Dynabeads His-Tag isolation and pulldown kit, or antibodies of interest and the Protein A/G magnetic beads were used. The pull-down samples generated were subjected to immunoblot assays.

#### Prediction of CMTM4 binding domains, protein sequence of truncated CMTM4 and NFκB activity measurement

Eukaryotic Linear Motif (ELM) resource (http://elm.eu.org/) was used to predict potential binding domains on CMTM4. DNA sequences that translate to the protein sequence below were synthesized and inserted into plasmid pCDNA3.1. These plasmids were confirmed for no mutation and transfected to NF-κB luciferase reporter cells.

Truncated CMTM4 protein sequence without STAT5 binding domain: MRSGEELDGF EGEASSTSMI SGASSPYQPT TEPVSQRRGL AGLRCDPD RGALGRLKVA QVILALIAFI CIETIMACSPCEGLCEGLYFFEFVSCSAFVVTGVLLIMFSLNLHMRIPQINWNLTDLVNTGLSA FLFFIASIVLAALNHRAGAE IAAVIFGFLA TAAYAVNTFL AVQKWRVSVR QQSTND RTESRDVDSR PEIQRLDTFS YSTNVTVRKK SPTNLLSLNH WQLA.

Truncated CMTM4 protein sequence without leucine-zipper motif (LZM) binding domain: MRSGEELDGF EGEASSTSMI SGASSPYQPT TEPVSQRRGL AG ALIAFI CIETIMACSP CEGLYFFEFV SCSAFVVTGVLLIMFSLNLH MRIPQINWNL TDLVNTGLSA FLFFIASIVL AALNHRAGAEIAAVIFGFLA TAAYAVNTFL AVQKWRVSVR QQSTNDYIRA RTESRDVDSR PEIQRLDTFS YSTNVTVRKK SPTNLLSLNH WQLA.

Truncated CMTM4 protein sequence without leucine-zipper motif (LZM) and TRAF6 binding domain: MRSGEELDGF EGEASSTSMI SGASSPYQPT TEPVSQRRGL AG ALIAFI FEFV SCSAFVVTGVLLIMFSLNLH MRIPQINWNL TDLVNTGLSA FLFFIASIVL AALNHRAGAEIAAVIFGFLA TAAYAVNTFL AVQKWRVSVR QQSTNDYIRA RTESRDVDSR PEIQRLDTFS YSTNVTVRKK SPTNLLSLNH WQLA.

Full length CMTM4 protein sequence: MRSGEELDGF EGEASSTSMI SGASSPYQPT TEPVSQRRGL AGLRCDPDYL RGALGRLKVA QVILALIAFI CIETIMACSP CEGLYFFEFV SCSAFVVTGV LLIMFSLNLH MRIPQINWNL TDLVNTGLSA FLFFIASIVL AALNHRAGAE IAAVIFGFLA TAAYAVNTFL AVQKWRVSVR QQSTNDYIRA RTESRDVDSR PEIQRLDTFS YSTNVTVRKK SPTNLLSLNH WQLA.

Traf6 DN DNA sequence:

cgcgcggggc ggcagcatgc ggggcggcga ggagctggac ggcttcgagg gcgaggcctc gagcacctcc atgatctcgg gcgccagcag cccgtaccaa cccaccaccg agccggtgag ccagcgccgc gggctggccg gcctgcgctg cgacccggac tacctgcgcg gcgcgctcgg ccgcctcaag gtcgcccaag tgattctggc cttgattgca tttatctgca tcgagactat catggagtgc tccccgtgcg aaggcctcta cttctttgag ttcgtaagct gcagtgcatt tgtggtgact ggggtcctgc tgattctctt cagcctcaac cttcatatga ggatccccca gatcaactgg aatctaacag atttggtcaa cactggactc agcactttct ttttctttat cgcctcaatc gtgttggctg ctctaaacca taaaaccgga gcggaaattg ctgccgtgat atttggcttc ttggcaacag cagcctatgc agtgagcaca ttcctggcca tgcagaaatg gagagtcagc gtccgccagc agagcactaa tgactacatc cgagctcgca ccgagtcgag agatgtggac agccgccctg agatccagcg cctggacaca tga.

To measure NF-κB activity of these stably transfected cells, we used Dual-Glo^R^ Luciferase Assay System and followed instruction. Briefly, NF-κB reporter plasmid and Renilla plasmid (internal control) were transfected to the target cells. After 48 h, cells were plated in 96-well plate and added with luciferase subtract to measure luciferase activity. Right after measurement, Stop N Glo solution was added to assess Renilla activity. The NF-κB signals were normalized by the Renilla activity.

#### Isolation and sorting of myeloid-derived suppressor cells (MDSCs)

C57BL/6 mice were injected subcutaneously with 5 × 10^5^ Lewis lung carcinoma (LLC) cells. Mice were sacrificed when tumors reached 1.5 × 1.5 cm^2^. Splenocytes and bone marrow were processed to single-cell suspensions. MDSCs were enriched by Percoll density gradient. Cells from the middle layer were stained with anti-Ly6C and anti-Gr-1 antibodies in the presence of FcR block, followed by sorting into monocytic (Gr-1^Lo^Ly6c^Hi^) and polymorphonuclear (PMN) (Gr-1^Hi^Ly6c^Lo^) populations using the MoFlo XPD High-Speed Cell Sorter.

#### In vivo tumor growth rate comparisons

In all, 5 × 10^5^ control or CMTM4 KD tumor cells were inoculated into the flanks of BALB/c (4T1 cells) or C57BL/6 (B16 or LLC cells) mice. Tumor sizes were measured every 3–5 days.

#### Suppression assays

The suppressive activity of MDSCs was assessed in peptide-mediated proliferation assays of TCR transgenic T cells as previously described (Huang et al, 2006b). Briefly, 10^5^ splenocytes from OT-II mice were co-cultured with serial dilutions of MDSCs in the presence of OVA peptides (1 µg/mL) in 96-well plates (Corning). Proliferation was determined based on [^3^H]-thymidine uptake after 48 h of co-culture.

#### Antibodies and flow cytometry

Flow cytometry staining was performed by Fc block of suspended cells, followed by appropriate antibodies incubation at 4 degree for 20 min. Flow cytometry acquisition was performed using FACS Canto II or FACS LSR Fortessa (BD Biosciences) instruments and data was analyzed using Flowjo software (Flowjo, LLC).

#### Adoptive transfer experiments

In total, 7 × 10^4^ control or CMTM4 KD LLC cells were inoculated into the livers of CD45.1 C57BL/6 mice. MDSCs were harvested from these mice and sorted into monocytic and PMN populations. Sorted MDSCs were then adoptively transferred via tail vein injection (5 × 10^6^ cells per mouse) into MaFIA mice that had been hepatically implanted with control or CMTM4 KD LLC tumor cells 14 days earlier. Before adoptive transfer, tumor-bearing MaFIA mice were depleted of CD115^+^ cells as previously described (Ma et al, 2011). Mice were sacrificed 4 days after adoptive transfer. Spleen, bone marrow, and tumor were harvested and the percentage, number, and phenotype of MDSCs were assessed by flow cytometric analysis.

#### Real-time PCR

RNA was isolated using Trizol per the manufacturer’s specifications. cDNA was synthesized from 1 µg of total RNA using M-MLV reverse transcriptase and qPCR was performed in 384-well plates using FastStart SYBR Green Master Mix on a ViiA™ 7 RT-PCR system.

#### ELISA assay

Secretion of G-CSF was determined using ELISA kits according to the manufacturer’s instruction.

#### TCGA data analysis

For the different stages of lung adenocarcinoma, R package (TCGABiolinks (Mounir et al, 2019)) was used to pull TCGA clinical data and RNAseq data. Patients were separated by overall stage and their CMTM4 gene expression was compared (FPKM).

For the tumor vs. normal CMTM4 gene expression in various cancers, boxplot analysis was used looking only at CMTM4 from the GEPIA2 (Tang et al, 2019) web server (http://gepia2.cancer-pku.cn/). GEPIA2 web server hosts build-in gene expression data form The Cancer Genome Atlas Program (TCGA, archived in Jan 2016) and Genotype-Tissue Expression (GTEx, archived in 2017) databases processed by Vivian et al (2017) (Vivian et al, 2017).

#### Survival analysis

The data were downloaded from the PROGgeneV2 prognostic database. The lung cancer dataset GSE26939 includes human lung adenocarcinoma mRNA expression and gene mutations from 115 samples (Wilkerson et al, 2012). The breast cancer dataset GSE37751 includes molecular profiles of 60 human breast cancer samples and their association with tumor subtypes and disease prognosis (Affymetrix) (Terunuma et al, 2014). The adrenal cancer dataset GSE33371 includes beta-catenin status effects in human adrenocortical carcinomas (33 samples) and adenomas (22 samples) (Heaton et al, 2012). Normal adrenal cortex (10 samples) and GSE19776 include adrenocortical carcinoma gene expression profiling of 21 samples (Legendre et al, 2016). The brain cancer dataset GSE7696 includes glioblastoma from a homogenous cohort of treated patients enrolled in a clinical trial (76 samples) (Murat et al, 2008) and GSE4271 includes molecular subclasses of high-grade glioma sorted by prognosis, disease progression, and neurogenesis (76 samples) (Phillips et al, 2006). The neuro-endocrine cancer dataset GSE62564 includes 497 samples (Wang et al, 2014). Myeloid leukemia (156 samples) and head and neck squamous cell carcinoma (290 samples) datasets were obtained from TCGA. To investigate the prognostic value of CMTM4 expression, the samples were partitioned into two groups using median CMTM4 expression levels, and log-rank tests were performed to compare the Kaplan–Meier curves of the two patient groups.

#### Transcriptome analysis

LLC control and CMTM4 KD cells were subjected to RNAseq analysis. RNA sample quality and quantity were assessed using Nanodrop, agarose gel electrophoresis, and Agilent 2100. DNA library preparation was performed using NEBNext Ultra DNA Library Prep Kit for Illumina (New England Biolabs, Ipswich, MA, USA). Sequencing was performed on the Illumina Hiseq X Ten at 150 bp paired end reads with 20 M read depth. All samples had Q30 > 90%. Both library preparation and sequencing were performed by Novogene (Sacramento, California). Differential gene analysis was performed using the HISAT2-Cufflinks workflow. Gene ontology enrichment analysis and visualization were performed in http://cbl-gorilla.cs.technion.ac.il/. The online data analysis tool Ingenuity pathway analysis (IPA) (Qiagen) was used for genes that had a *P* value of <0.05 and a ≥ twofold change (FC) difference between control and CMTM4 KD cells. Core analysis was run on this dataset to determine the pathways most affected by the loss of CMTM4.

#### Reverse phase protein array (RPPA)

LLC control and CMTM4 KD cells were subjected to RPPA experiments. RPPA data were generated by the RPPA core facility at the MD Anderson Cancer Center.

#### Statistical analysis

Statistical analyses were performed using GraphPad Prism 9. The results are presented as mean ± SD. A *P* value of <0.05 was considered to be statistically significant.

## Supplementary information

Appendix

Peer Review File

Dataset EV1

Dataset EV2

Source data Fig. 1

Source data Fig. 2

Source data Fig. 3

Source data Fig. 4

Source data Fig. 5

Source data Fig. 6

Source data Fig. 7

## Data Availability

The raw RNA-seq data and RPPA data are deposited to GEO with accession number: GSE280512. It is available at https://www.ncbi.nlm.nih.gov/geo/query/acc.cgi?acc=GSE280512. The analyzed RNA-seq data and RPPA data are available as Dataset EV1 and Dataset EV2, respectively. The source data of this paper are collected in the following database record: biostudies:S-SCDT-10_1038-S44318-024-00330-y.
