# Supplementary material for: Modulation of tumor inflammatory signaling and drug sensitivity by CMTM4
Source: EMBO J. 2025 Feb 13;44(6):1866–83. doi: 10.1038/s44318-024-00330-y (PMC11914105; doi:10.1038/s44318-024-00330-y)
Supplement: Supplementary file 1 — Appendix [file 44318_2024_330_MOESM1_ESM.pdf]

Appendix for

Modulation of tumor inflammatory signaling and drug sensitivity by CMTM4

Table of Contents

Table of Content 1
Appendix Figure S1 2
Appendix Figure S2 3
Appendix Figure S3 4
Appendix Figure S4 5
Appendix Figure S5 6
Appendix Figure S6 7
Appendix Figure S7 8
Appendix Figure S8 9
Appendix Figure S9 10
Appendix Figure S10 11
Appendix Table S1 12

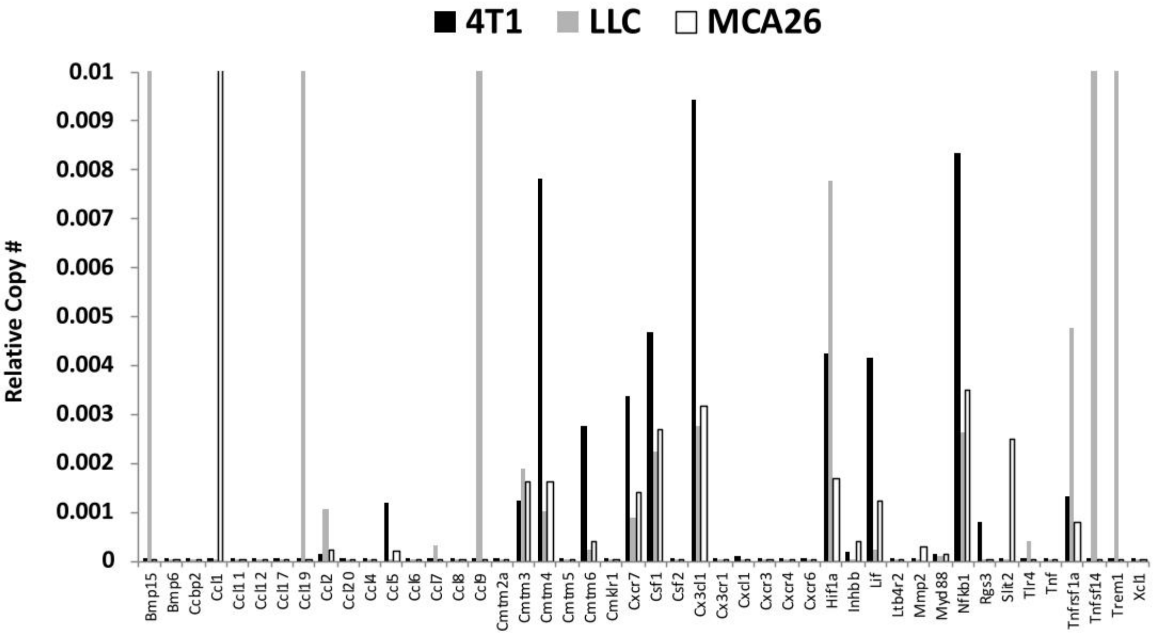

Appendix Figure S1. CMTM4 is highly expressed in multiple cancer types.

Gene profiling of multiple murine cancer cell lines including 4T1 breast cancer cells, LLC lung carcinoma cell line, and MCA26 colon cancer cells was performed using chemokine RT-PCR super arrays.

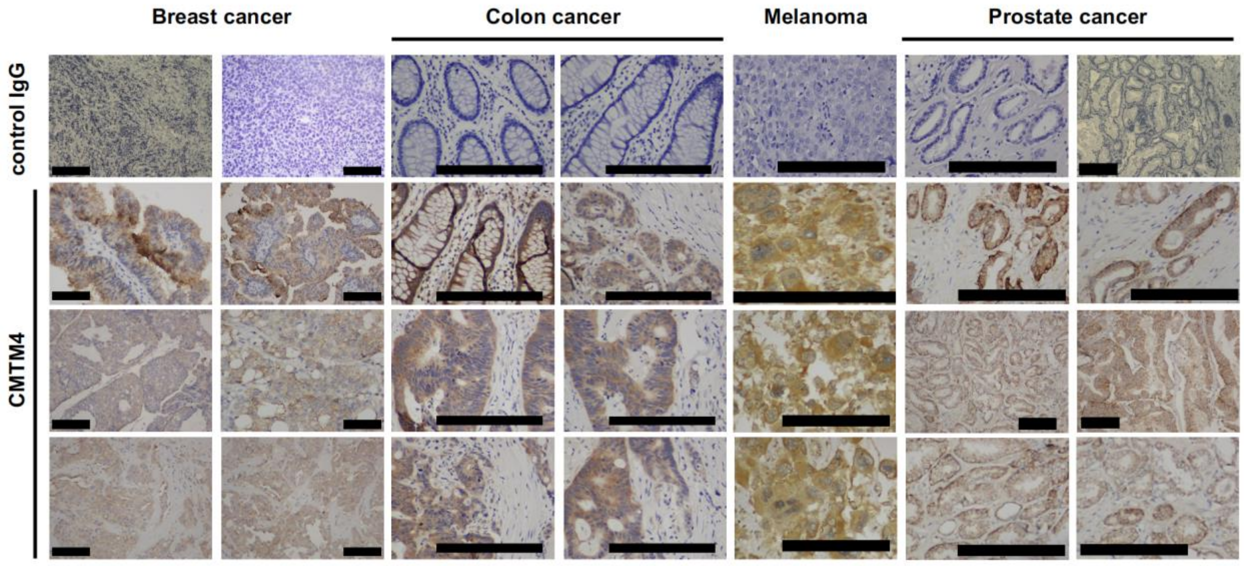

Appendix Figure S2. Paraffin-embedded human carcinoma sections were stained with anti-CMTM4 antibody. scale bars = 200 μm.

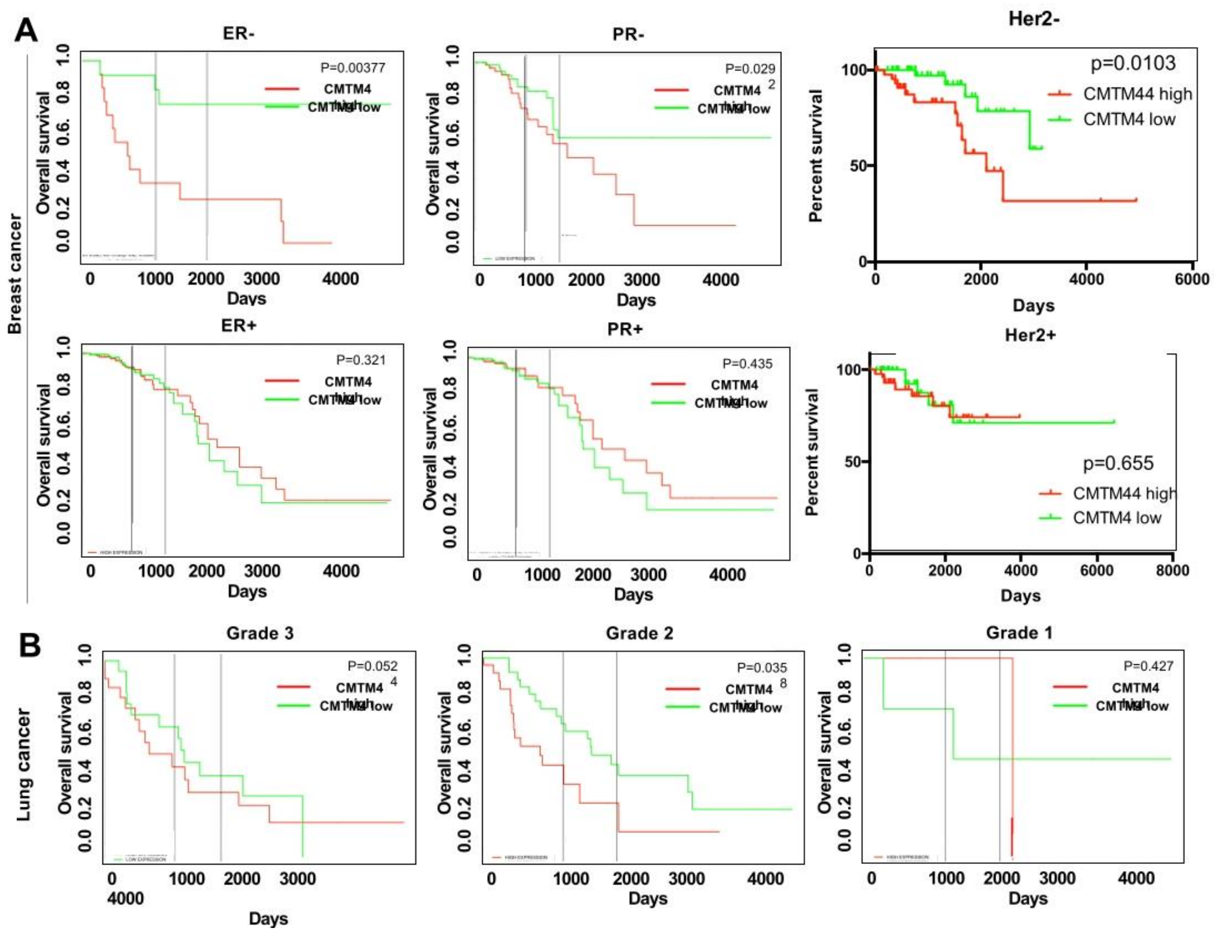

Appendix Figure S3. Survival curves of breast and lung cancer patients stratified with co-variates.

(**A)** Survival curves of breast cancer patients stratified by ER, PR and Her2 status, comparing between CMTM4 high (red) vs. low (green) expression groups based on median expression values. (**B**) Survival curves of lung cancer patients stratified by tumor grades, comparing between CMTM4 high (red) vs. low (green) expression groups based on median expression values. P value calculated by Log-rank test.

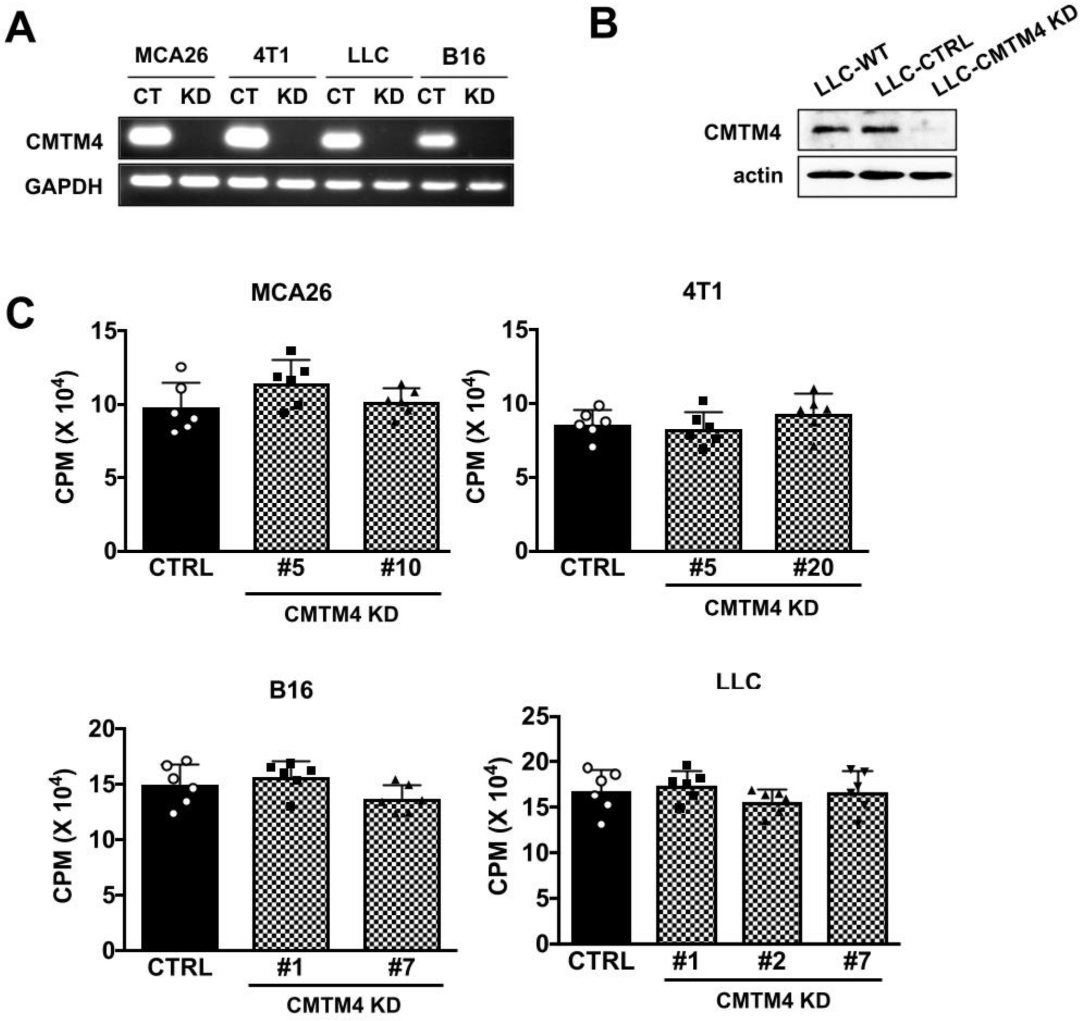

Appendix Figure S4. CMTM4 KD has no effect on cell proliferation *in vitro*.

(**A-B)** Plasmids containing sequences corresponding to shRNA targeting CMTM4 or control vector were transfected into various cancer cell lines. Inhibition of CMTM4 expression was confirmed by RT-PCR (**A**) and western blot (**B**). (**C)** Tumor cells (10^4/well) were cultured in 96-well flat plates. After 2 days, proliferation was determined based on [^{3}H]-thymidine uptake.

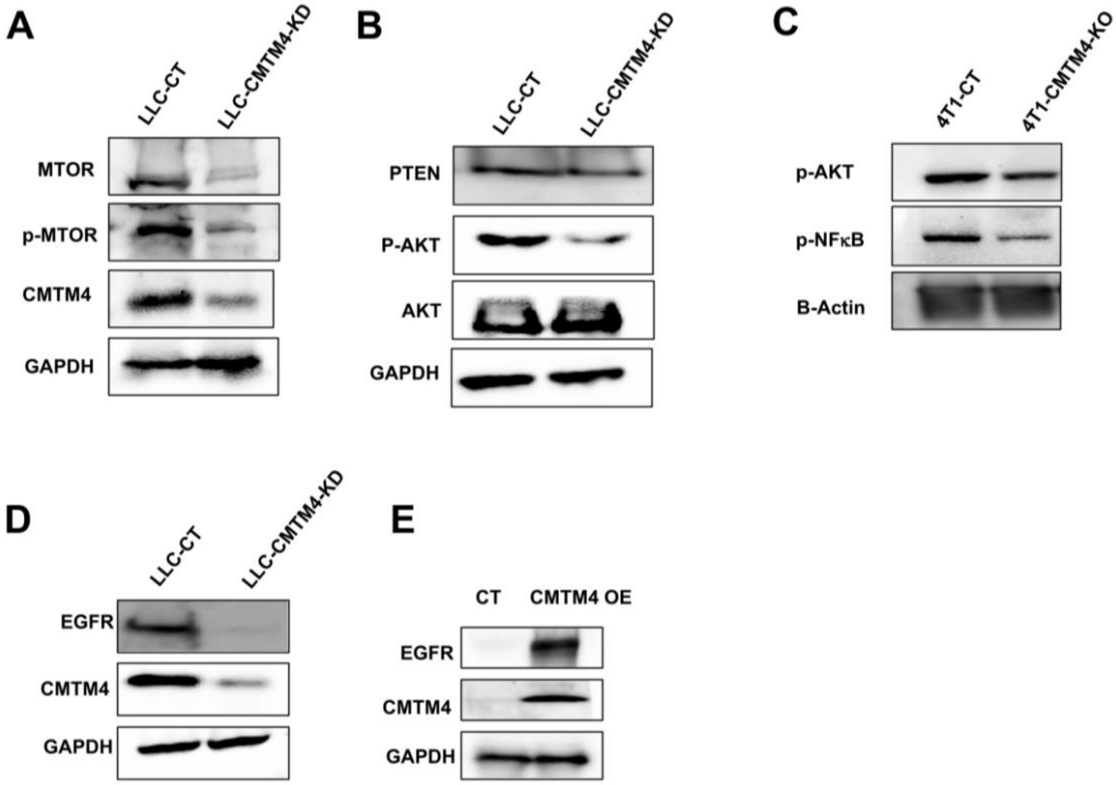

Appendix Figure S5. CMTM4 regulates pAKT/mTOR pathway and EGFR expression in mouse and human cancer cell lines.

(**A-B**) Repeated experiment of detecting Akt signaling and mTOR pathway activation in LLC control and LLC CMTM4 KD cells. (**C**) Western blot of phosphor-Akt and phosphor-NFκB in 4T1 breast cancer cell line with or without CMTM4 KO. (**D**) Repeated experiment of detecting EGFR in control or CMTM4 KD LLC cells by western blot. (**E**) Repeated experiment of detecting EGFR in 293T with or without CMTM4 transfection by western blot.

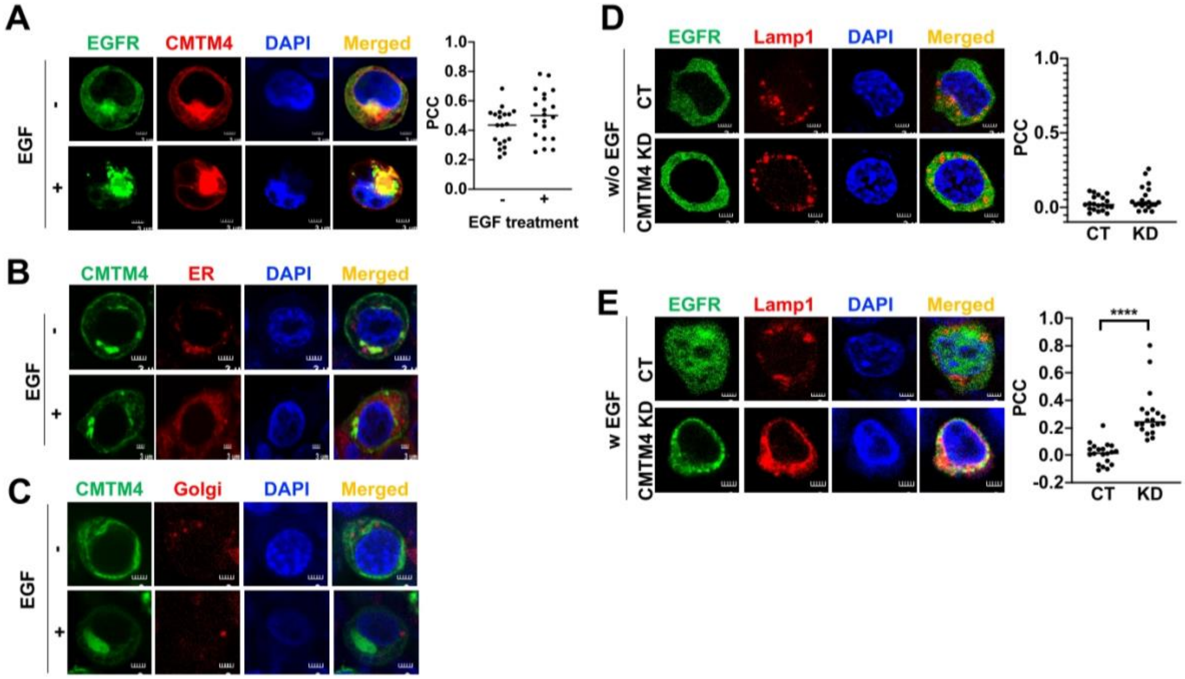

Appendix Figure S6. CMTM4 regulates EGFR and Rab proteins expression in mouse and human cancer cell lines.

(**A**) 293T cells were transfected with CMTM4-mCherry and EGFR-GFP. Cells were then treated with 10ng/ml EGF for 1 hour. (**B-C**) 293T cells were transfected with CMTM4-GFP and stained with ER tracker (**B**) or Golgi tracker (**C**) with and without EGF treatment. (**D-E**) EGFR-GFP and Lamp-1 RFP were transfected to LLC control and CMTM4 KD cells. Confocal imaging was taken with (**C**) and without (**D**) treatment with EGF. Representative confocal images are shown. Pearson correlation coefficient (PCC) between the GFP channel and mCherry channel was measured from 20 individual cells. Representative confocal images are shown. Pearson correlation coefficient (PCC) between the EGFR signal and CMTM4 signal was measured from 10 individual images. P value calculated by Mann-Whitney test. **p<0.01, ****P<0.0001. Scale bar: 3 μm.

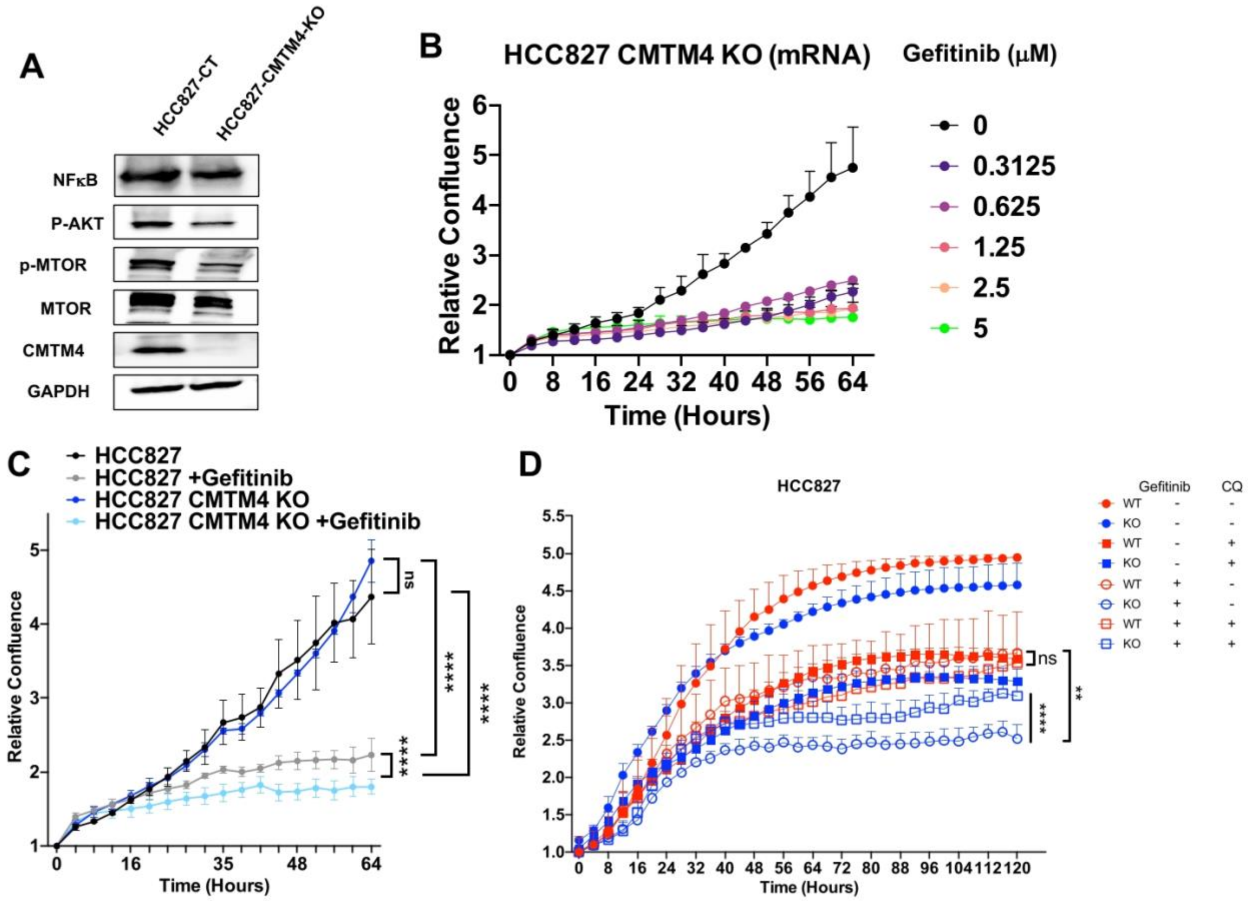

Appendix Figure S7. Loss of CMTM4 synergized with EGFR inhibitor to reduce human cancer cells growth.

(**A**) Repeated western blots of CMTM4 KO reduced phosphor-Akt and phosphor-mTOR signaling in HCC827 human cancer cell. (**B**): Growth curve of HCC827 cells with CMTM4 KO by Cas9 mRNA under various concentrations of Gefitinib measured by Incucyte. (**C**): HCC827 control and CMTM4 KO cells were treated with or without 2μM Gefitinib and tumor cell growth was measured by Incucyte. (**D**): HCC827 control and CMTM4 KO cells were treated with or without 2μM Gefitinib and 10μM Chloroquine (CQ). Tumor cell growth was measured by Incucyte. P value calculated by Two-way ANOVA test. ****: $p<0.0001$.

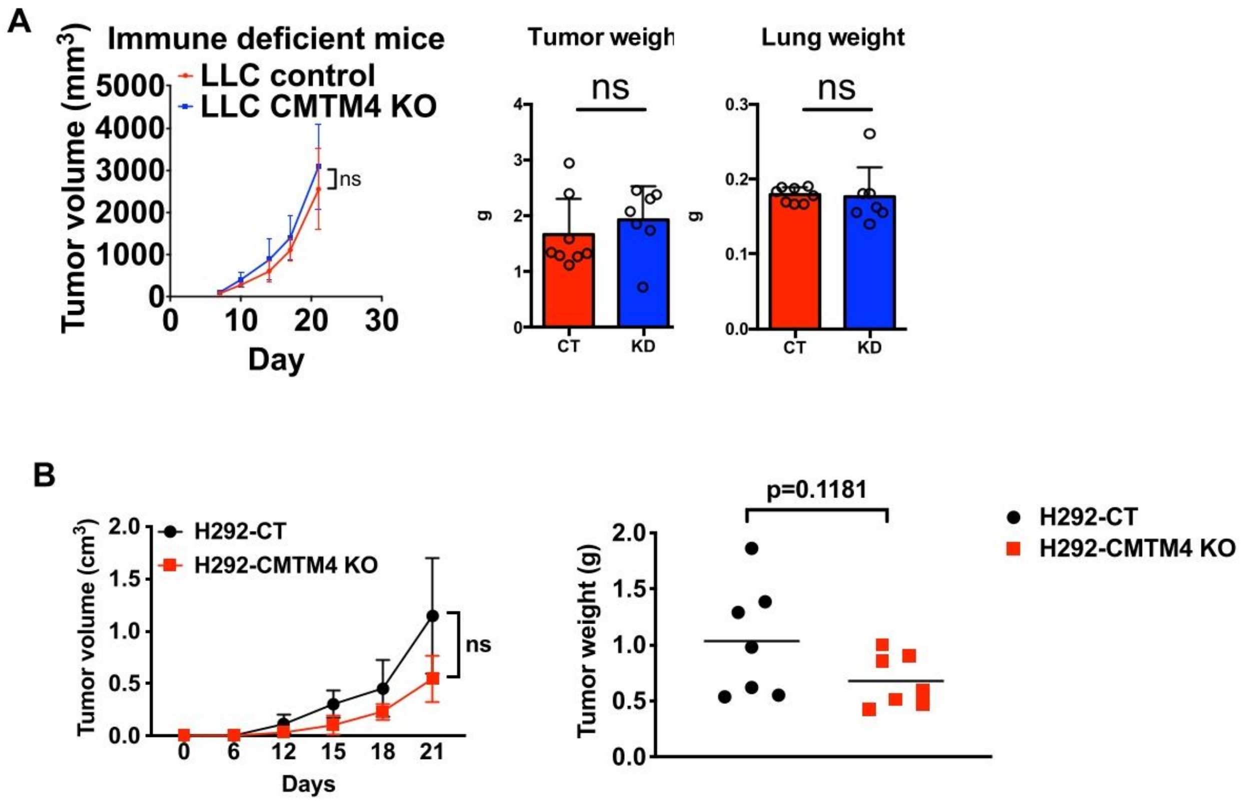

Appendix Figure S8. CMTM4 KO does not suppress tumor growth in immuno-deficient mice.

(**A-B**) Mouse lung cancer LLC (**A**) and human lung cancer cell H292 (**B**) control or CMTM4 KO cells were inoculated into NSG mice. Tumor growth was measured every 3 days. Tumors and/or lung were collected after 21 days of inoculation and weights were measured. P value calculated by Unpaired student t-test.

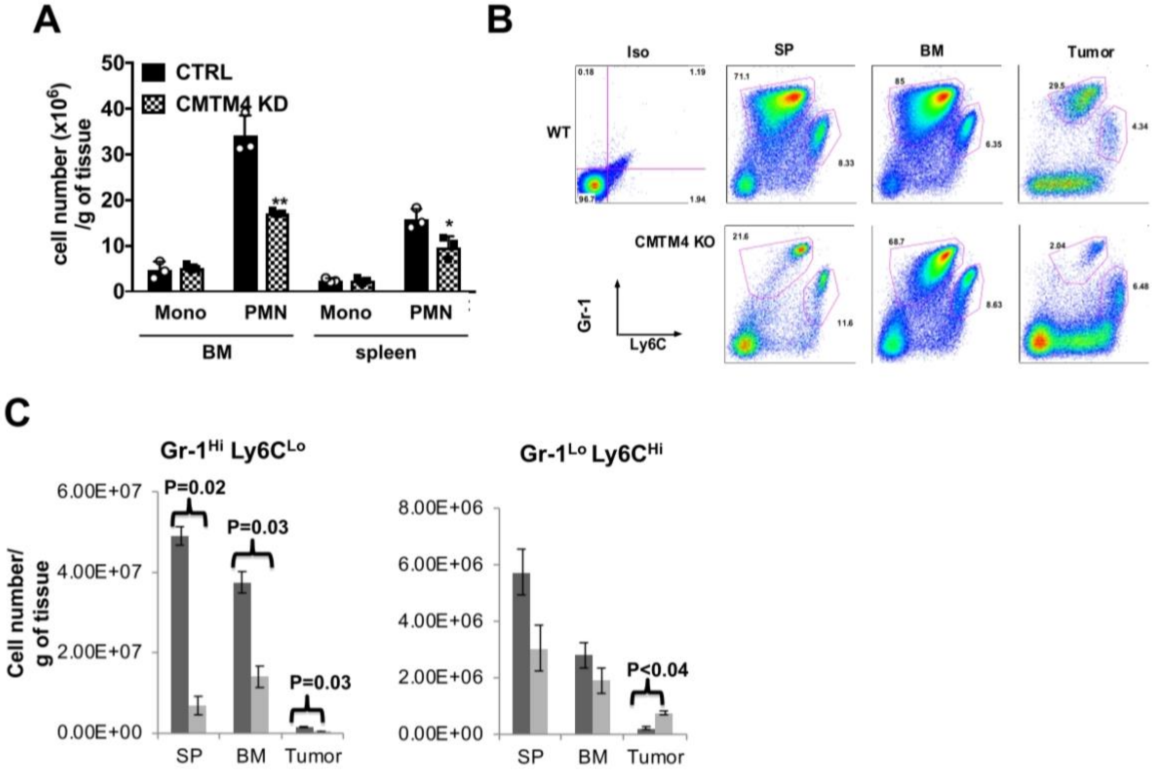

Appendix Figure S9. CMTM4 KD tumors reduce infiltration of PMN-MDSC

(A) Control or CMTM4 KD LLC cells were inoculated into C57BL/6 mice. Once tumors reached greater than 1x1 cm^2, mice with similarly sized tumors were sacrificed and bone marrow and spleen were harvested. The number of MDSCs in the bone marrow and spleen were determined. (**B-C)** 5 x 10^5 control or CMTM4 KD MCA26 cells were inoculated into BALB/c mice. Once tumors reached >1x1 cm^2, mice were sacrificed. Spleen, bone marrow, and tumor tissues were harvested. Single cell suspensions of leukocytes were prepared and the phenotype of MDSC was assessed by FACS analysis. (C) The numbers of PMN- and monocytic MDSC in spleen, bone marrow, and tumor were calculated. P value calculated by Unpaired student t test. *: p<0.05, **: p<0.01, ***: p<0.001.

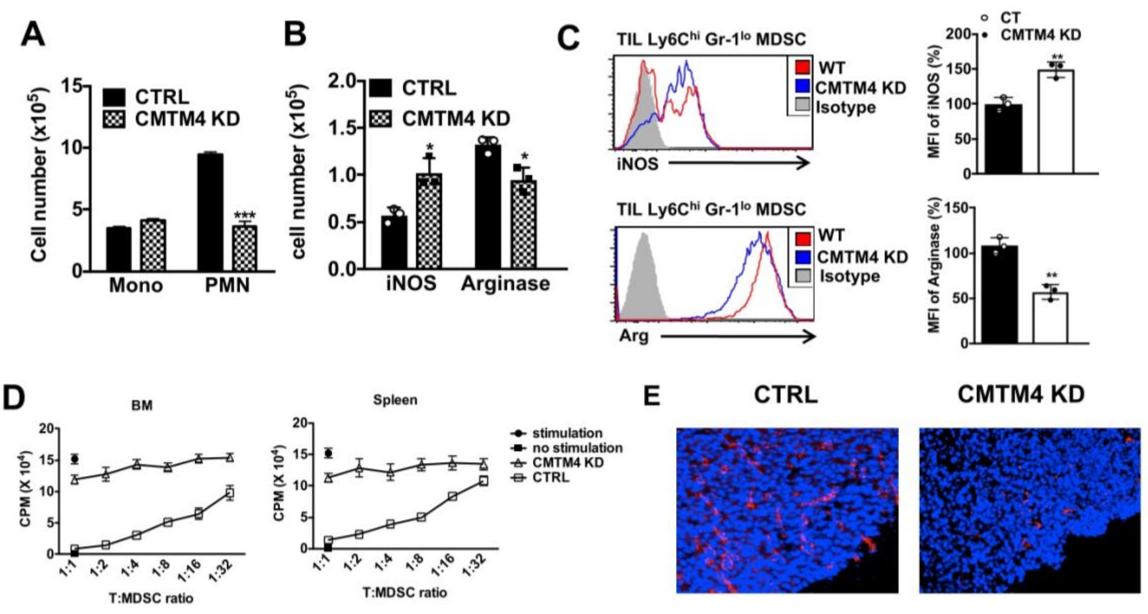

Appendix Figure S10. CMTM4 KD altered function of recruited MDSCs.

(**A&B**) MaFIA mice were intrahepatically inoculated with LLC control or CMTM4 KD tumors. MDSCs were harvested from CD45.1 LLC tumor-bearing mice and sorted into PMN and monocytic populations. When tumors reached 7 × 7 to 9 × 9 mm^2, CD115$^+$ cells were depleted. On the same day, sorted PMN-MDSC and M-MDSCs were intravenously injected into CD115-depleted MaFIA tumor-bearing mice. After recipient mice were terminated, the numbers of tumor-infiltrating donor (CD45.1) MDSCs (**A**) and levels of iNOS and arginase (**B**) were determined. The data shown are representative of three reproducible experiments. (**C)** Intracellular staining was performed to assess iNOS and arginase 1 (Arg) expression in tumor-infiltrating monocytic MDSCs. **(D)** MDSCs from bone marrow and spleen of LLC control or CMTM4 KD tumor-bearing mice were cultured with OT-II T cells in the presence of OVA peptides. Proliferation was determined based on [^{3}H]-thymidine uptake. (**E)** LLC control or CMTM4 KD cancer cells were intrahepatically inoculated. Immunofluorescent staining of tumor tissues was performed using anti-CD31 and DAPI on LLC control or CMTM4 KD (20X). P value calculated by Unpaired student t test. *: p<0.05, **: p<0.01, ***: p<0.001.

Significance of CMTM4 high vs low patient survival		
**Cancer type**	**p value**	**data source**
**Adrenal**	0.0778	GSE33371
	0.0229	GSE19776
**Brain**	0.05	GSE7696
	0.003	GSE4271
**Breast**	0.0027	GSE37751
(ER neg)	0.003	GSE37751
(PR neg)	0.02	TCGA
(Chemo pos)	0.001	GSE37751
**Myeloid Leukemia**	0.0277	TCGA
**HNC**	0.049	TCGA
**Lung**	0.02	GSE26939
(Grade 2)	0.035	GSE26939
**Neuro-endocrine cancer**	0.0002272	GSE62564

Appendix Table S1. Overall survival rates of cancer patients were correlated with CMTM4 expression levels.
