# Supplementary figures and images for: Modulation of tumor inflammatory signaling and drug sensitivity by CMTM4 (part 1 of 2)
Source: EMBO J. 2025 Feb 13;44(6):1866–83. doi: 10.1038/s44318-024-00330-y (PMC11914105; doi:10.1038/s44318-024-00330-y)

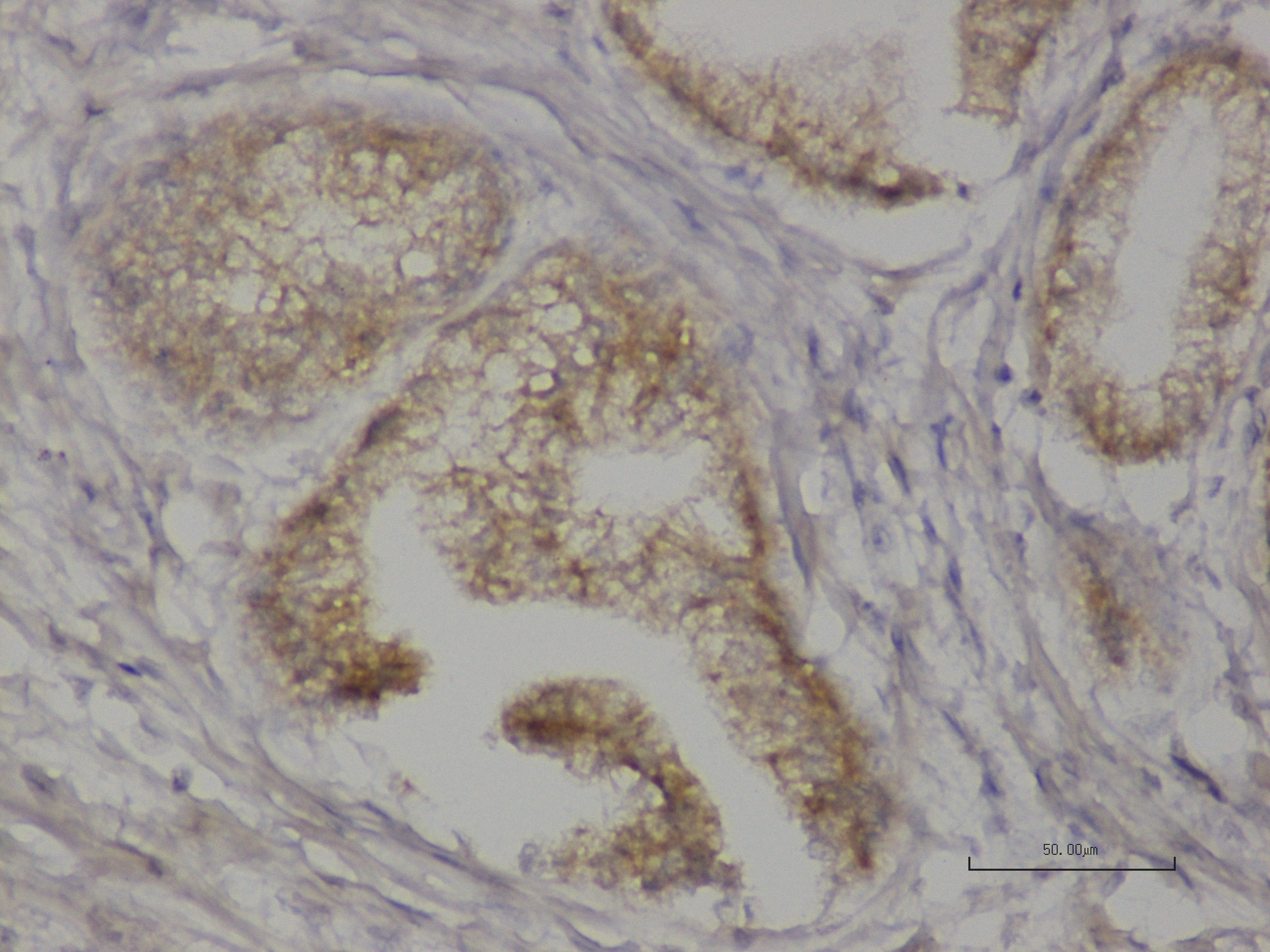

Supplement: Supplementary file 5 — Source data Fig. 1 [file 44318_2024_330_MOESM5_ESM.zip › Figure 1 source data/Figure 1B/Fig1B Prostate CMTM4.JPG]

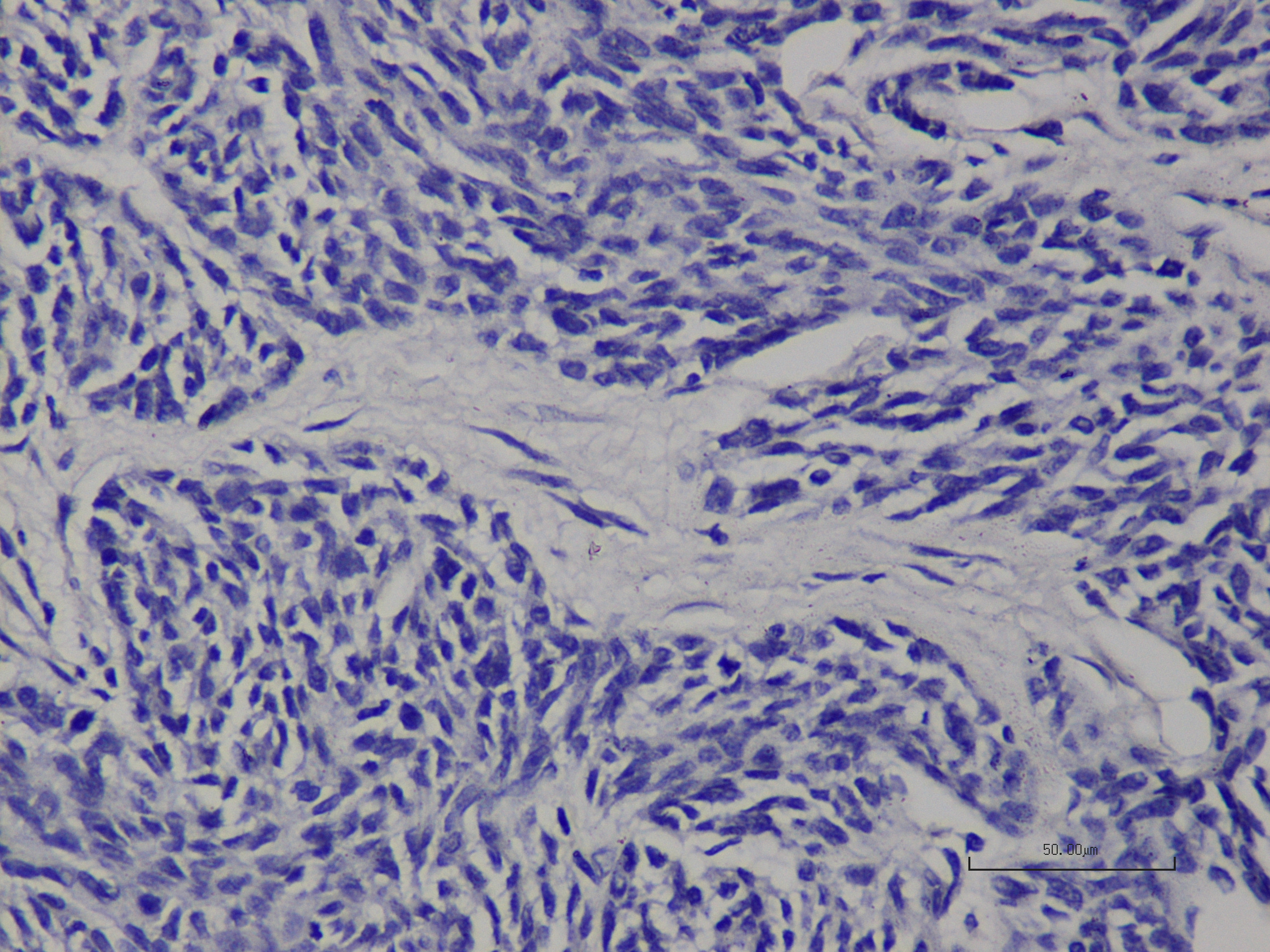

Supplement: Supplementary file 5 — Source data Fig. 1 [file 44318_2024_330_MOESM5_ESM.zip › Figure 1 source data/Figure 1B/Fig1B Melanoma control.JPG]

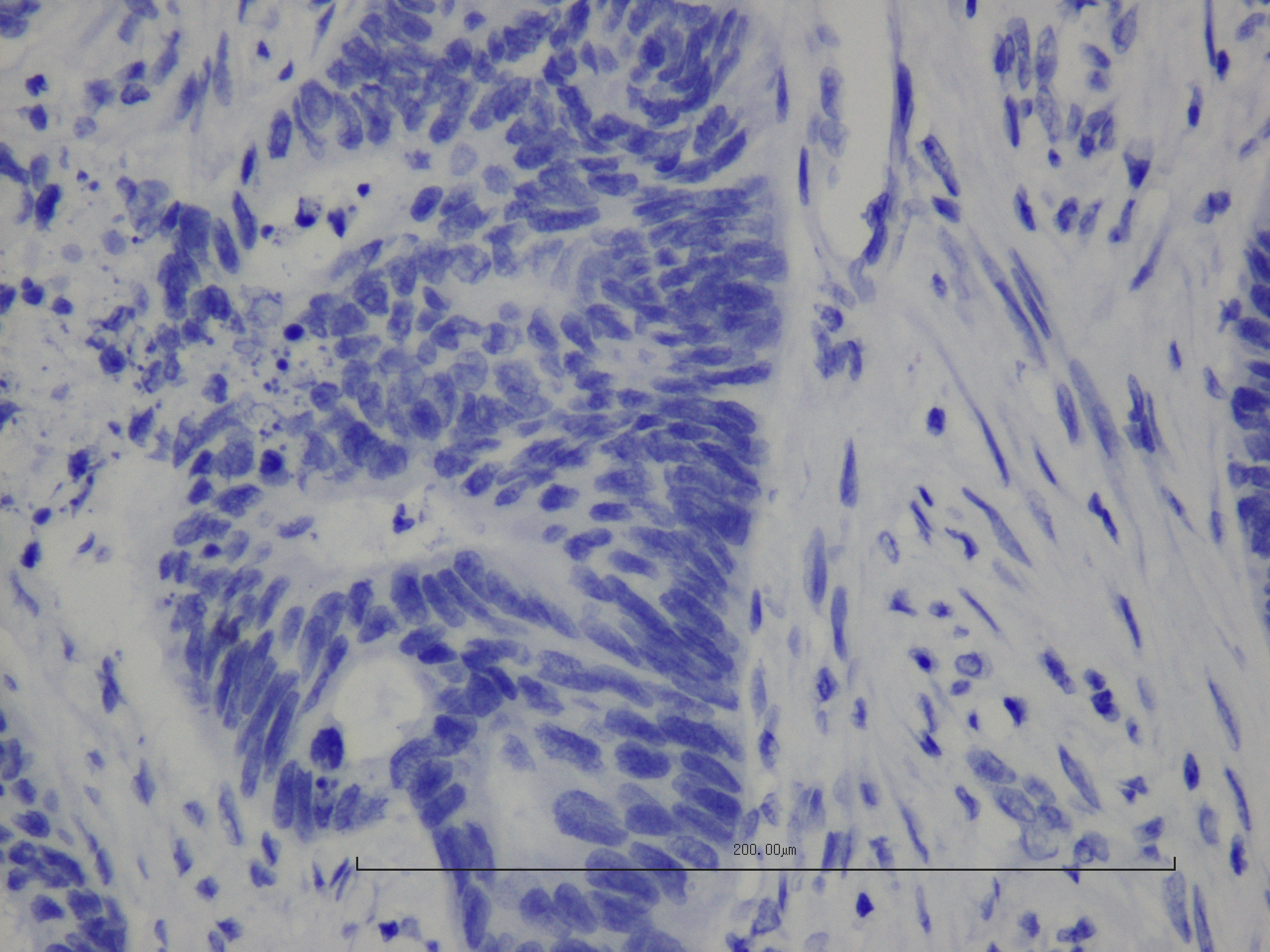

Supplement: Supplementary file 5 — Source data Fig. 1 [file 44318_2024_330_MOESM5_ESM.zip › Figure 1 source data/Figure 1B/Fig1B Colon control.JPG]

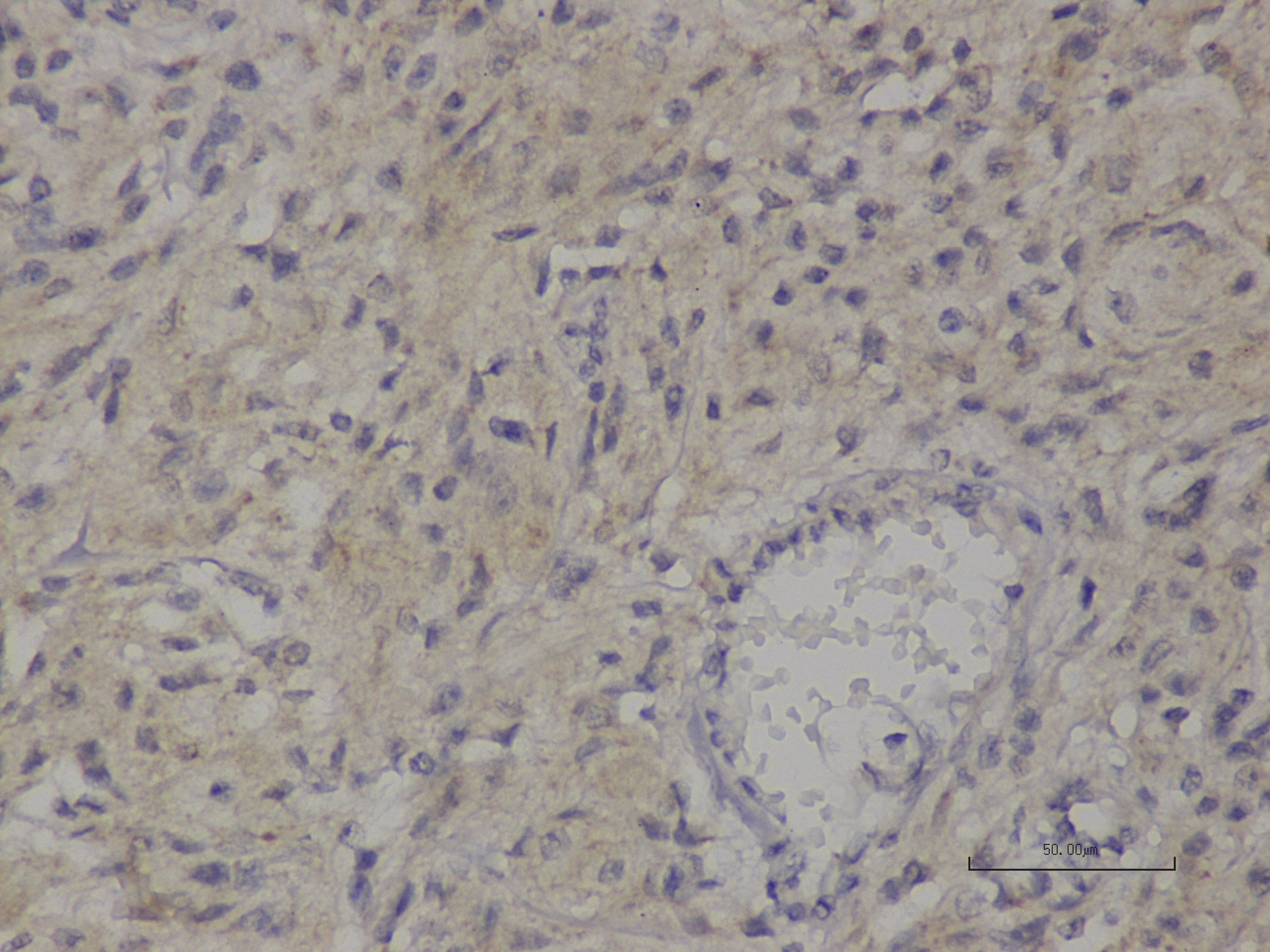

Supplement: Supplementary file 5 — Source data Fig. 1 [file 44318_2024_330_MOESM5_ESM.zip › Figure 1 source data/Figure 1B/Fig1B Glioma CMTM4.JPG]

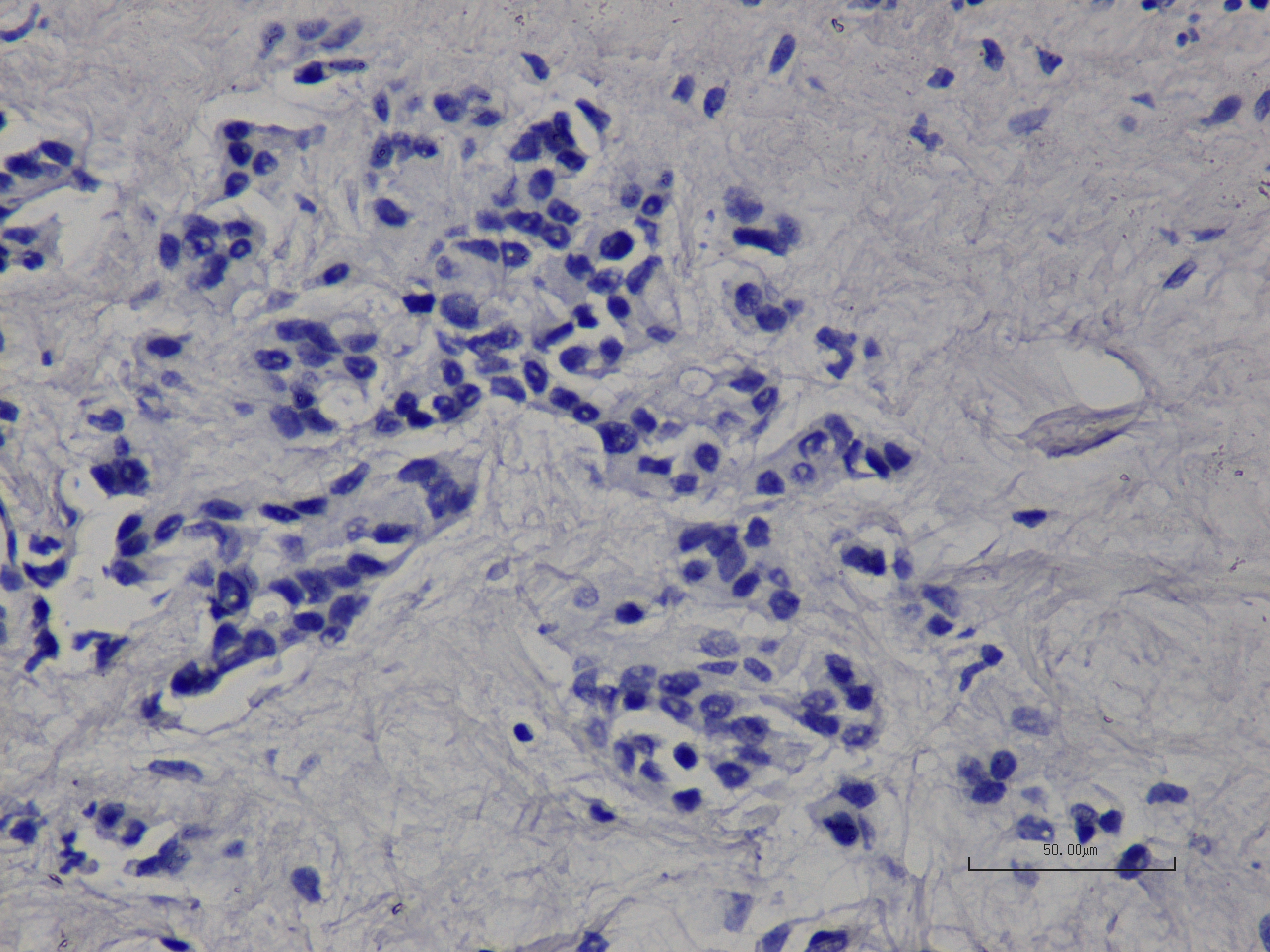

Supplement: Supplementary file 5 — Source data Fig. 1 [file 44318_2024_330_MOESM5_ESM.zip › Figure 1 source data/Figure 1B/Fig1B Breast control.JPG]

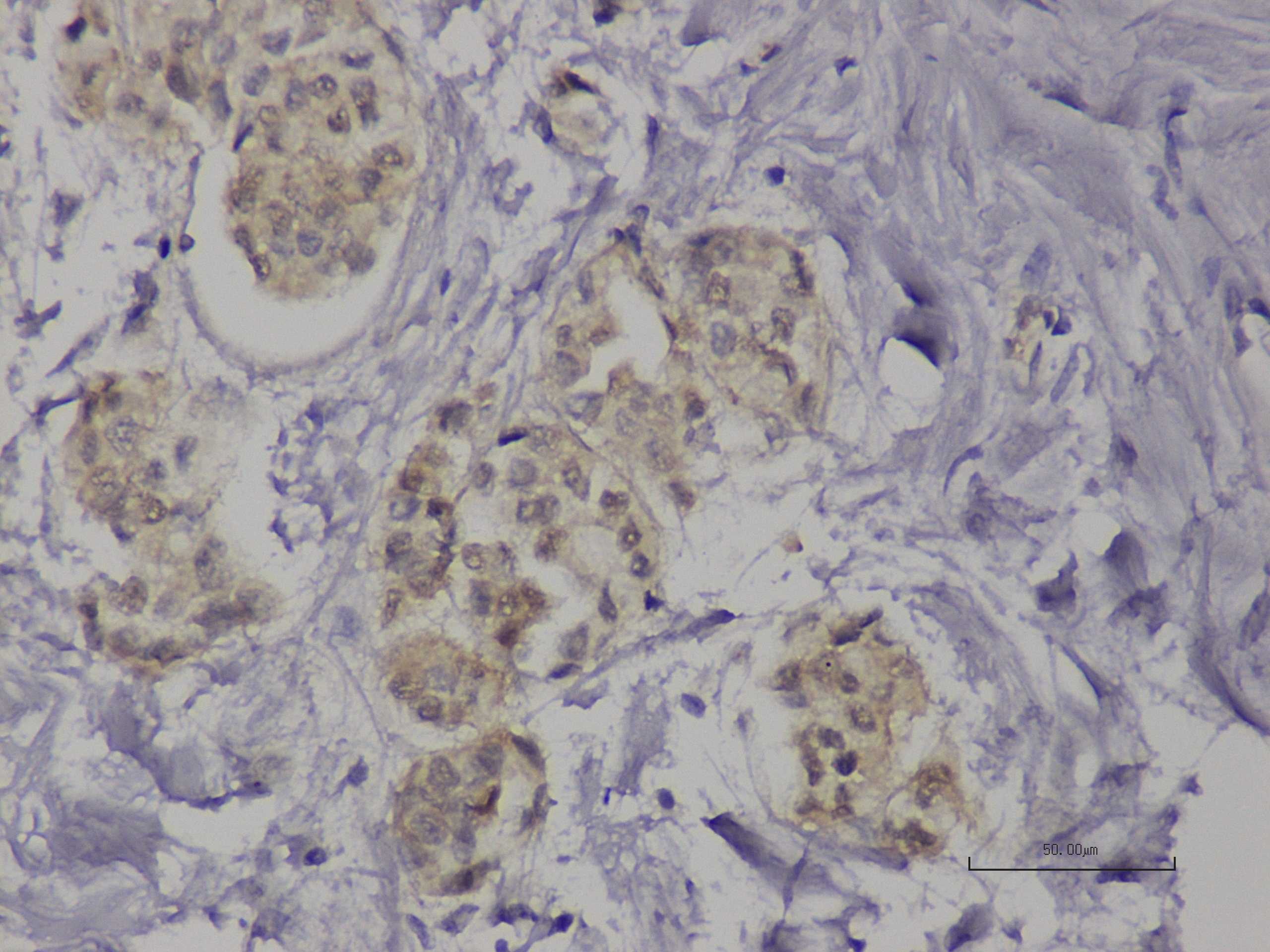

Supplement: Supplementary file 5 — Source data Fig. 1 [file 44318_2024_330_MOESM5_ESM.zip › Figure 1 source data/Figure 1B/Fig1B Breast CMTM4.JPG]

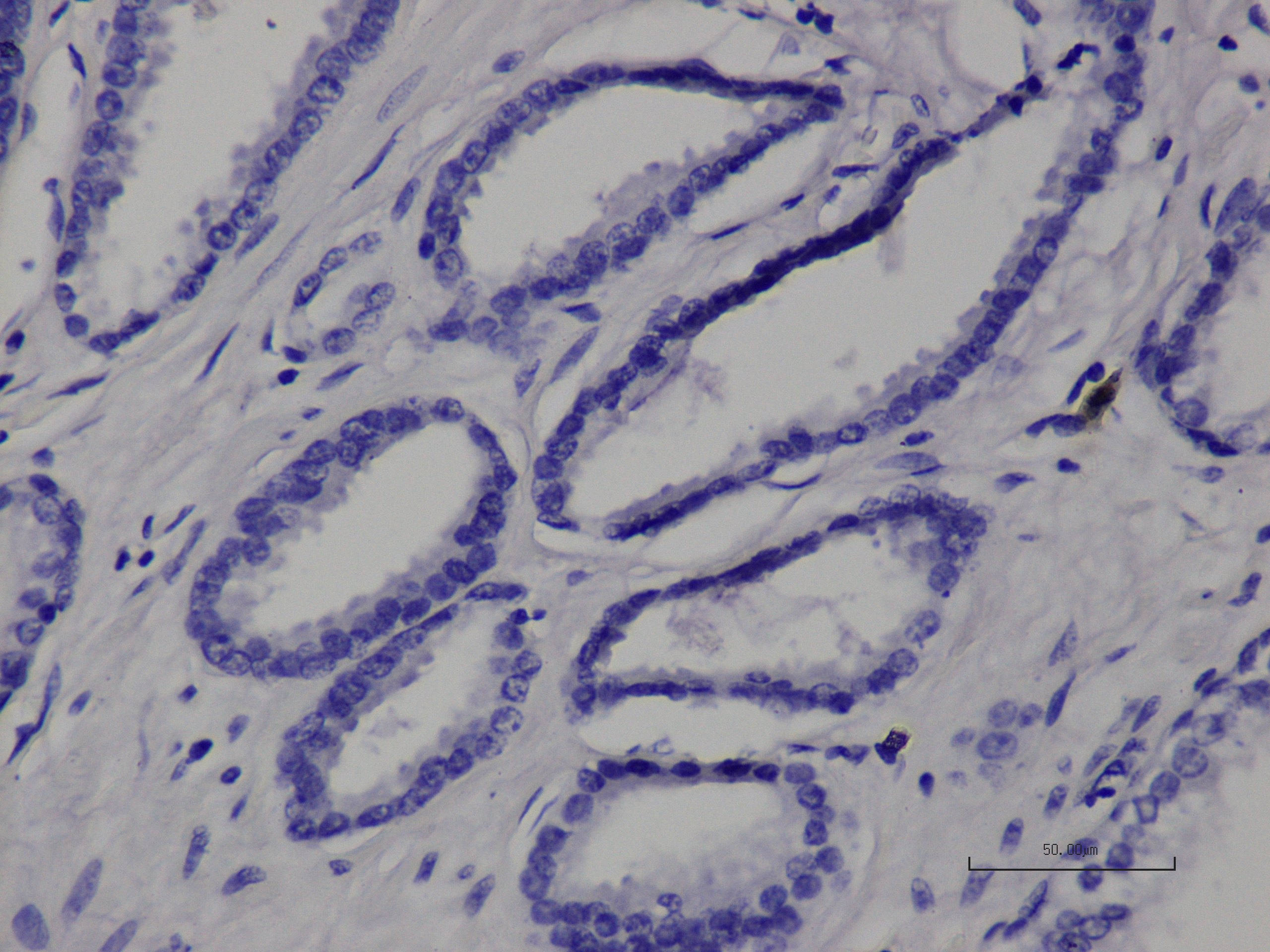

Supplement: Supplementary file 5 — Source data Fig. 1 [file 44318_2024_330_MOESM5_ESM.zip › Figure 1 source data/Figure 1B/Fig1B Prostate control.JPG]

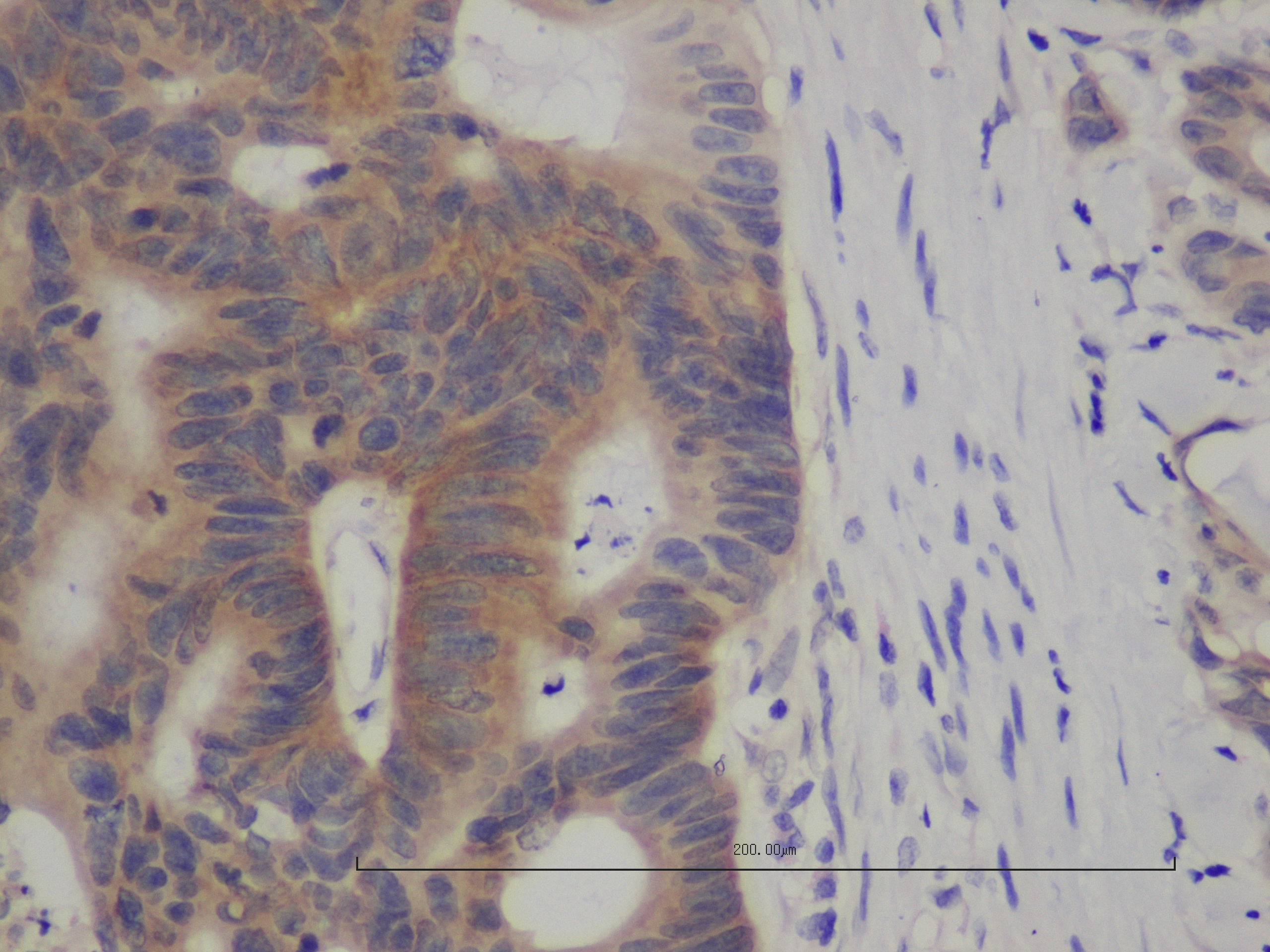

Supplement: Supplementary file 5 — Source data Fig. 1 [file 44318_2024_330_MOESM5_ESM.zip › Figure 1 source data/Figure 1B/Fig1B Colon CMTM4.JPG]

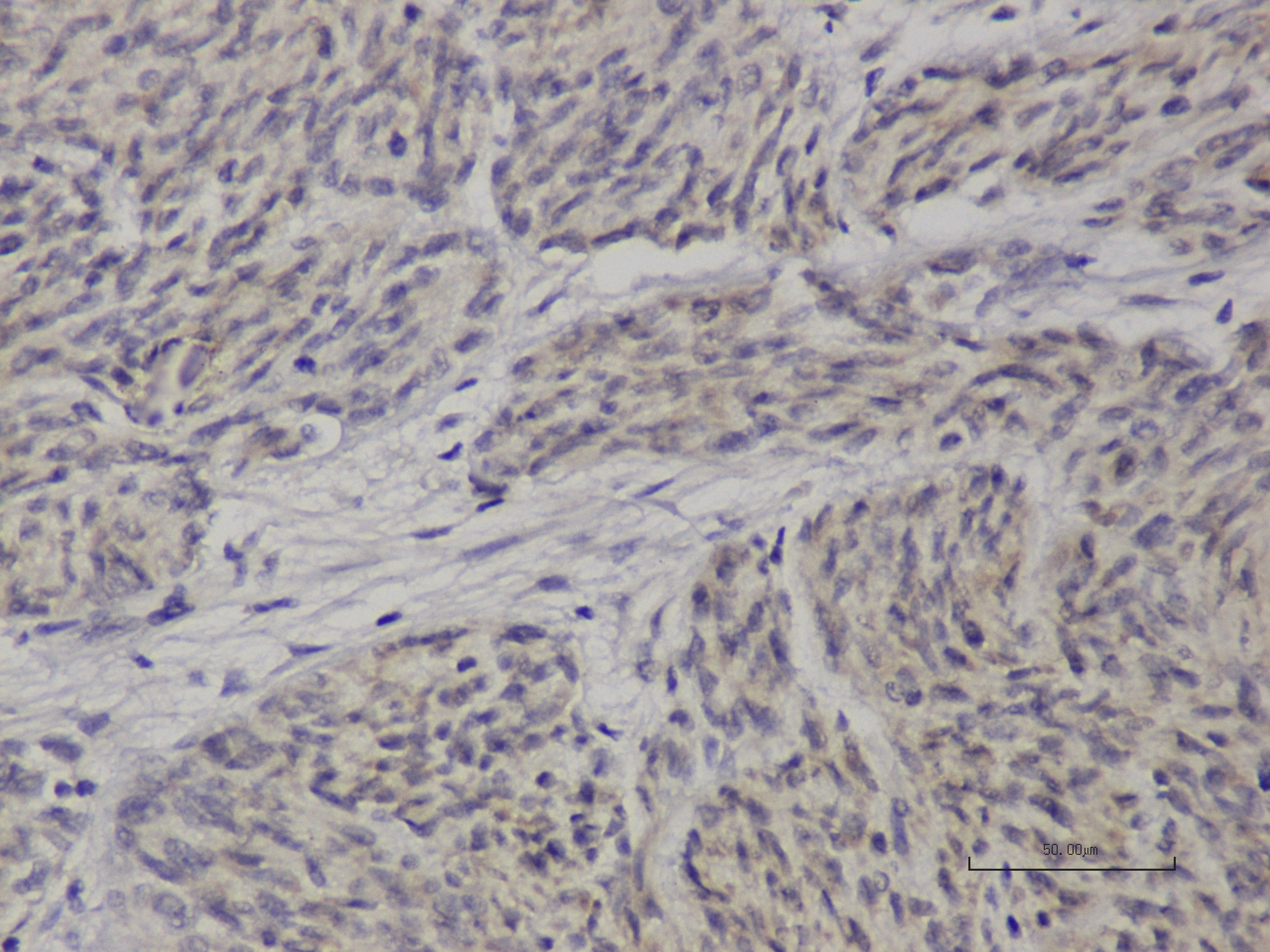

Supplement: Supplementary file 5 — Source data Fig. 1 [file 44318_2024_330_MOESM5_ESM.zip › Figure 1 source data/Figure 1B/Fig1B Melanoma CMTM4.JPG]

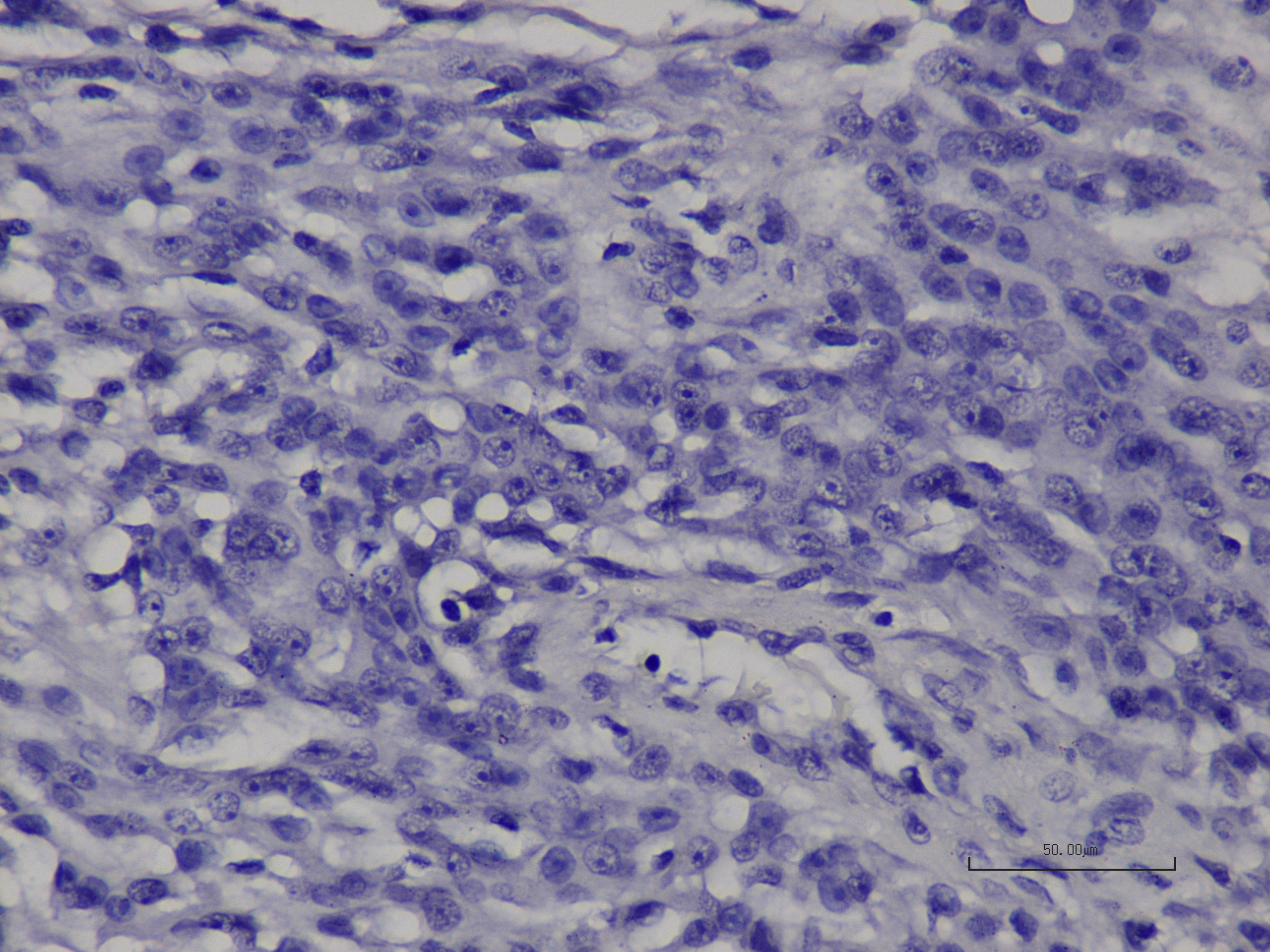

Supplement: Supplementary file 5 — Source data Fig. 1 [file 44318_2024_330_MOESM5_ESM.zip › Figure 1 source data/Figure 1B/Fig1B Glioma control.JPG]

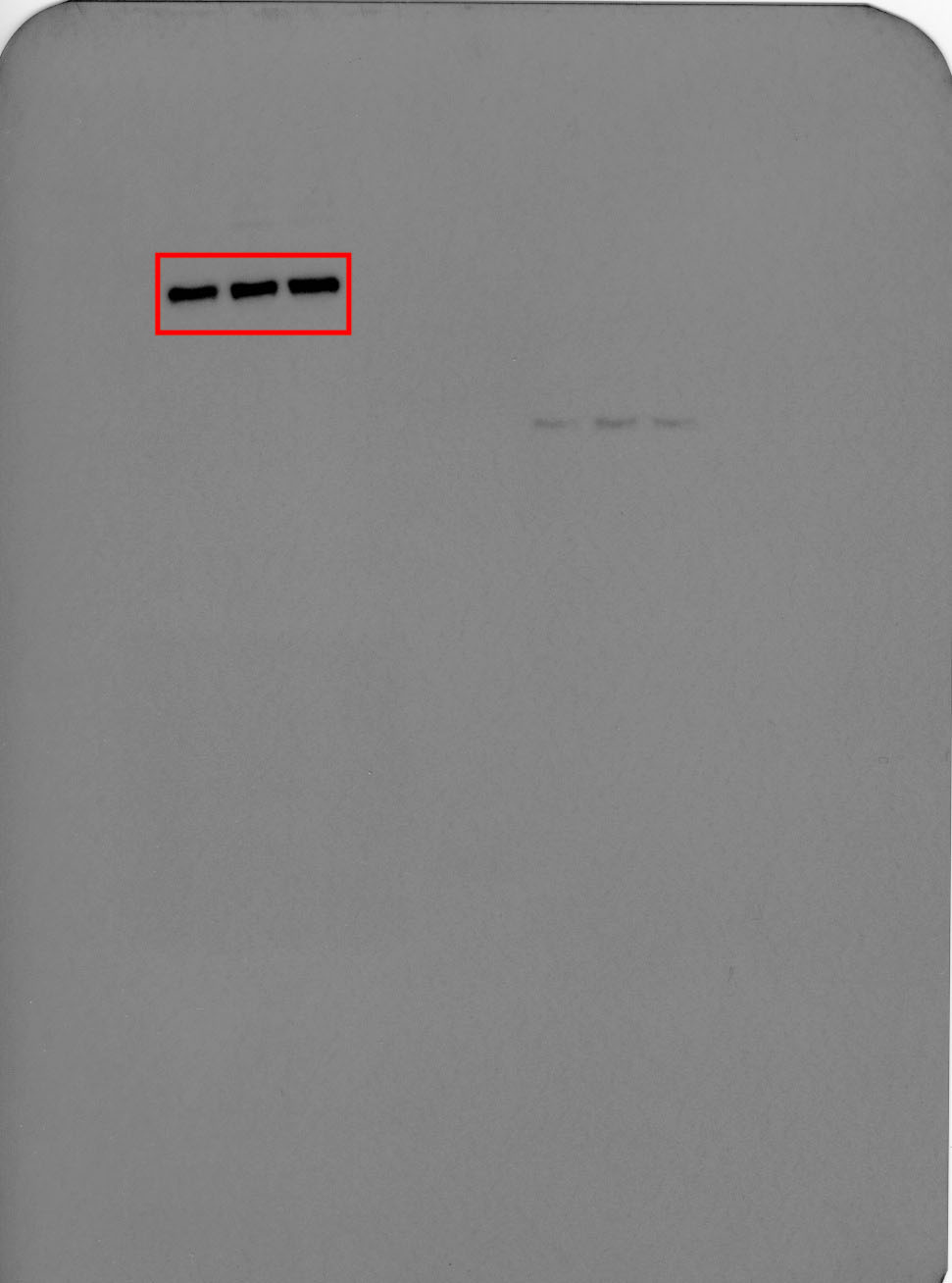

Supplement: Supplementary file 6 — Source data Fig. 2 [file 44318_2024_330_MOESM6_ESM.zip › Figure 2 source data/Figure 2F/Figure 2F NFkB.jpeg]

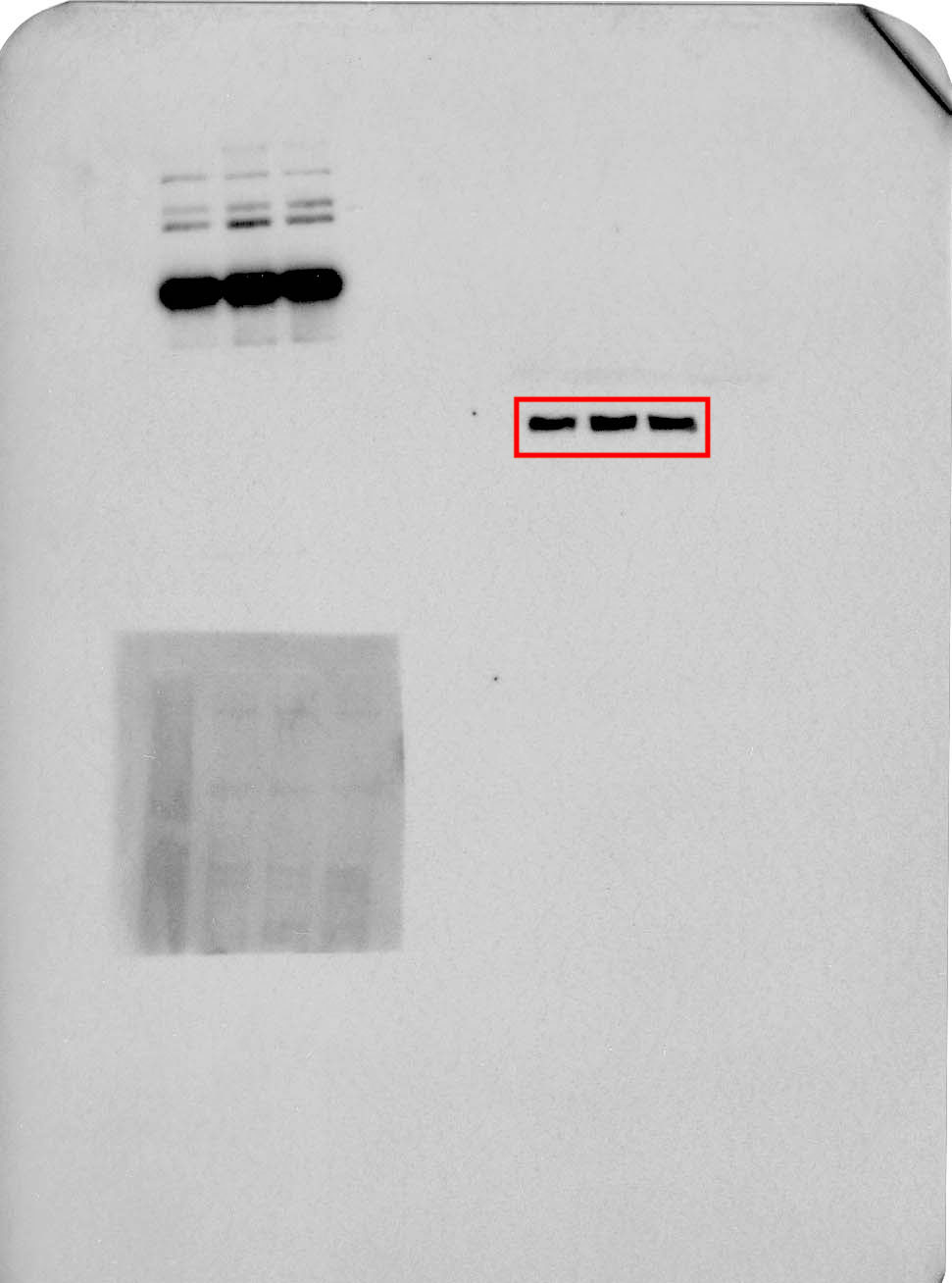

Supplement: Supplementary file 6 — Source data Fig. 2 [file 44318_2024_330_MOESM6_ESM.zip › Figure 2 source data/Figure 2F/Figure 2F actin.jpeg]

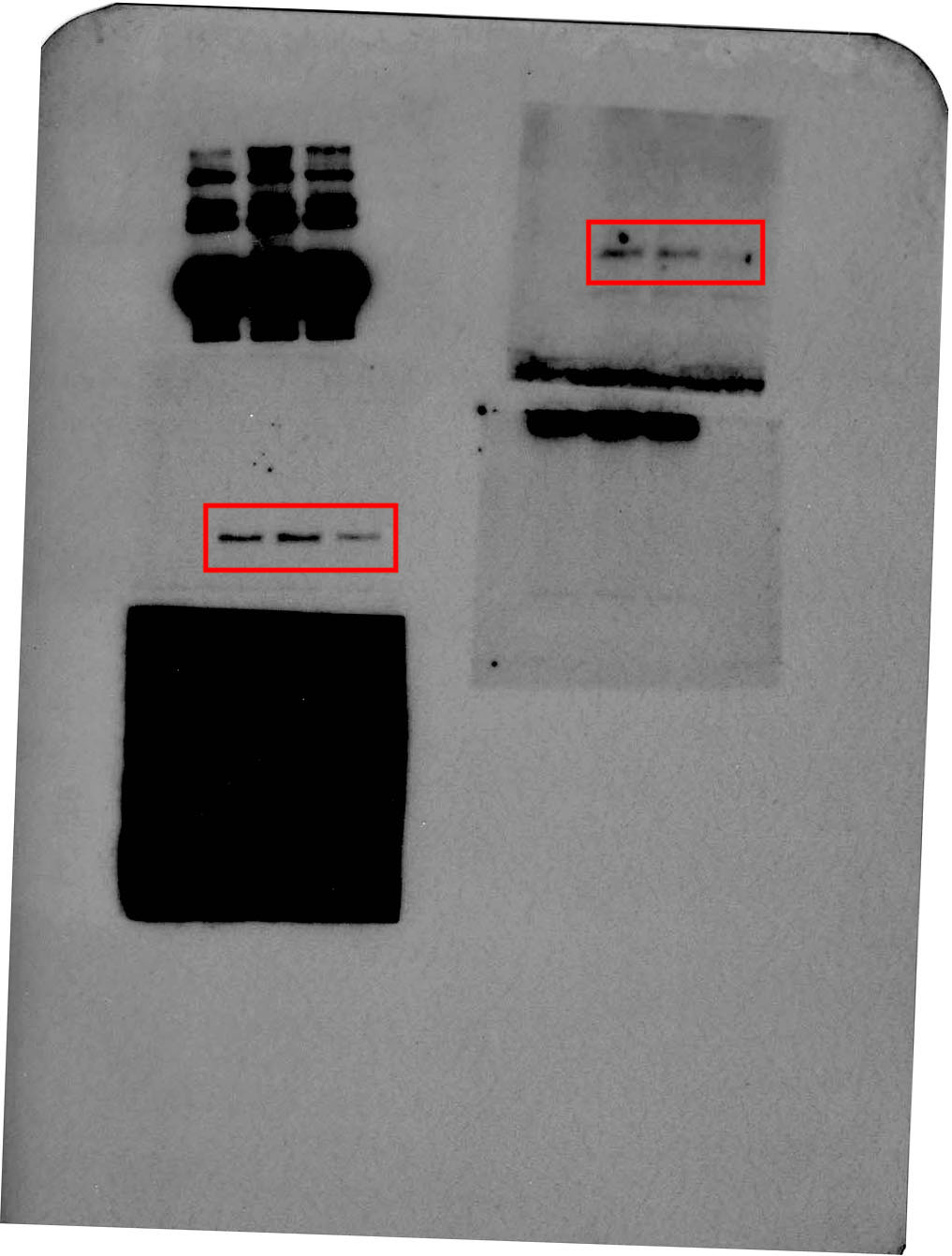

Supplement: Supplementary file 6 — Source data Fig. 2 [file 44318_2024_330_MOESM6_ESM.zip › Figure 2 source data/Figure 2F/Figure 2F pNFkB CMTM4.jpeg]

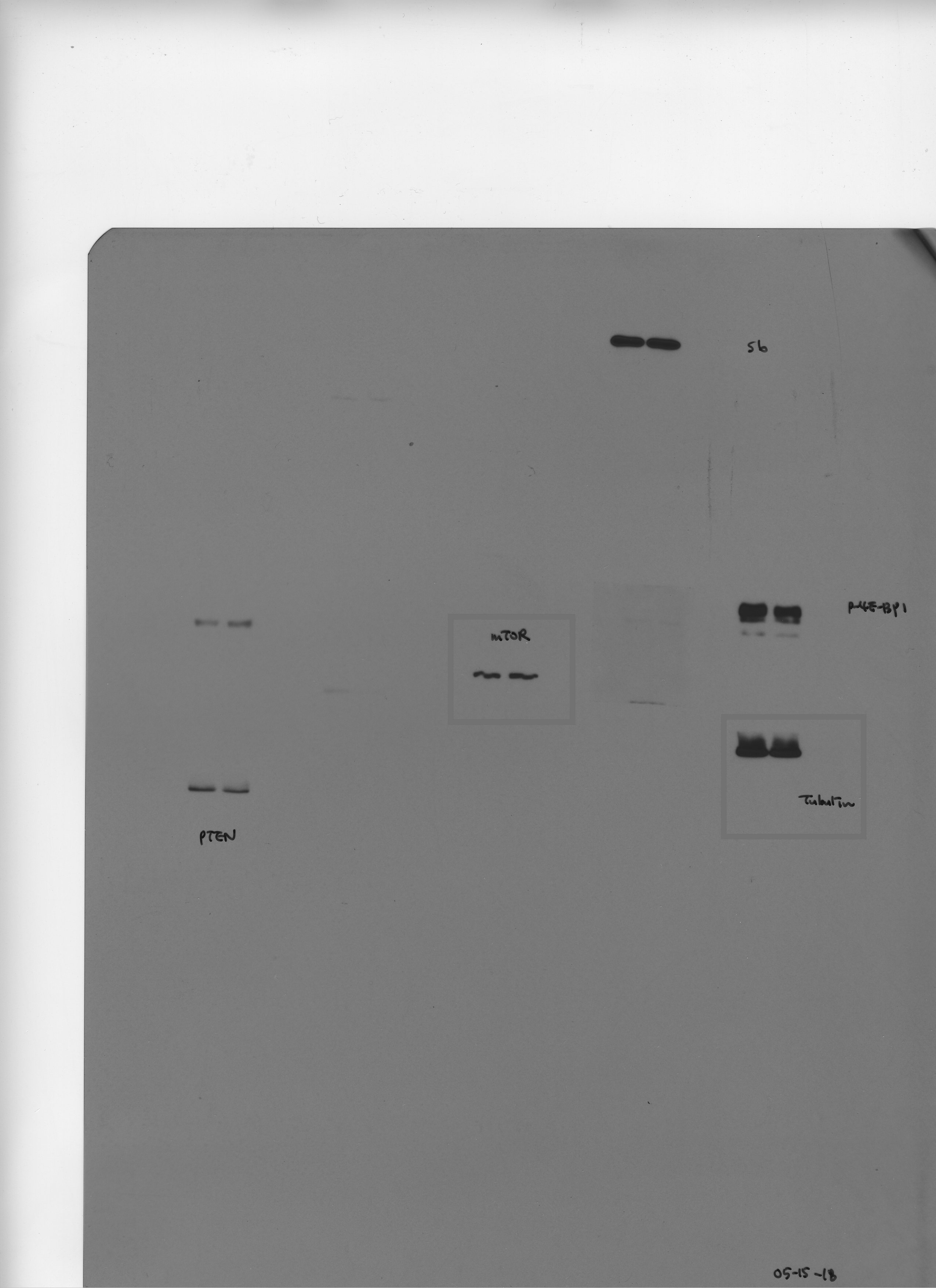

Supplement: Supplementary file 6 — Source data Fig. 2 [file 44318_2024_330_MOESM6_ESM.zip › Figure 2 source data/Figure 2E/Figure 2E mTOR tubulin.jpg]

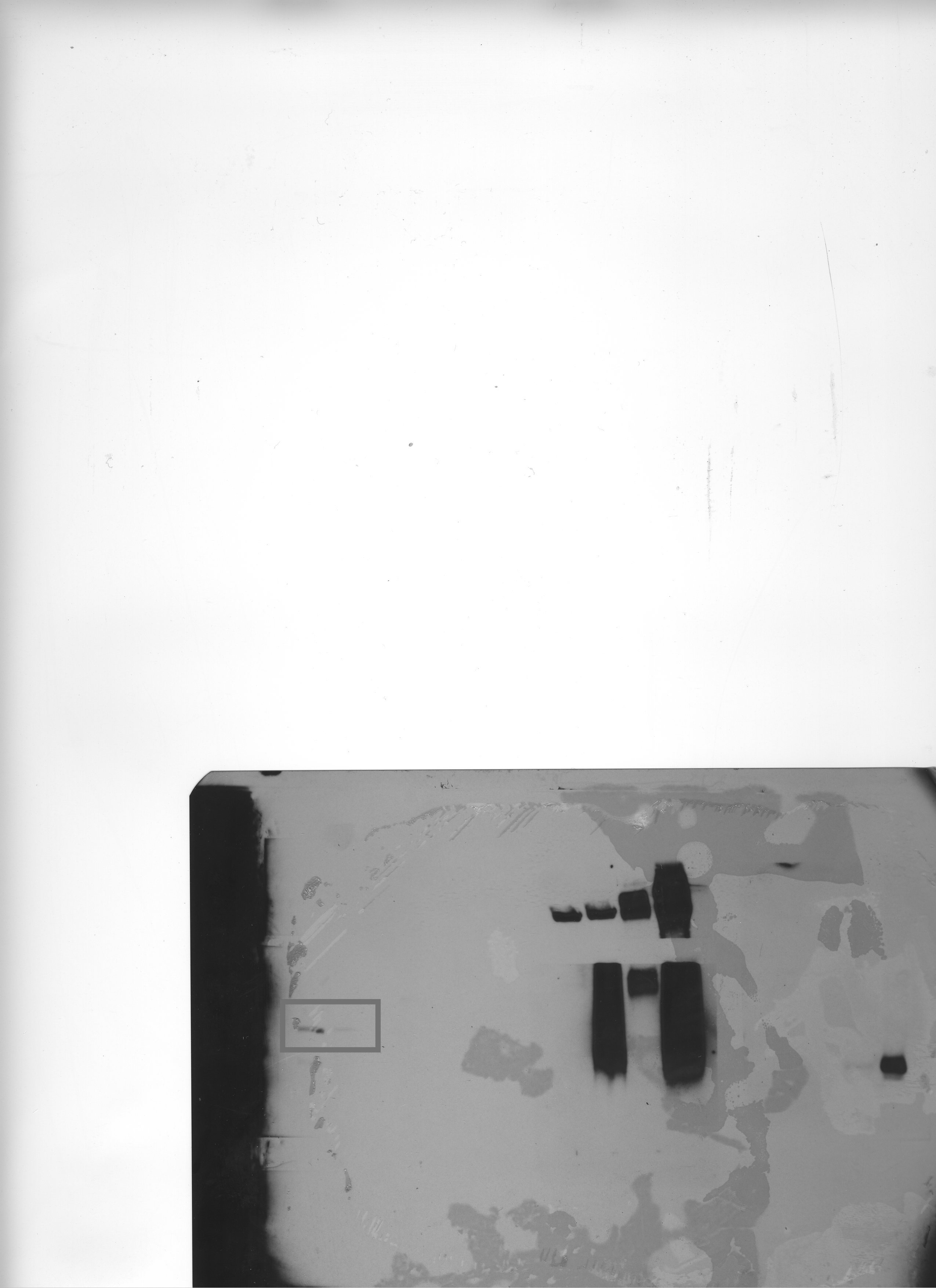

Supplement: Supplementary file 6 — Source data Fig. 2 [file 44318_2024_330_MOESM6_ESM.zip › Figure 2 source data/Figure 2E/Figure 2E pmTOR.jpg]

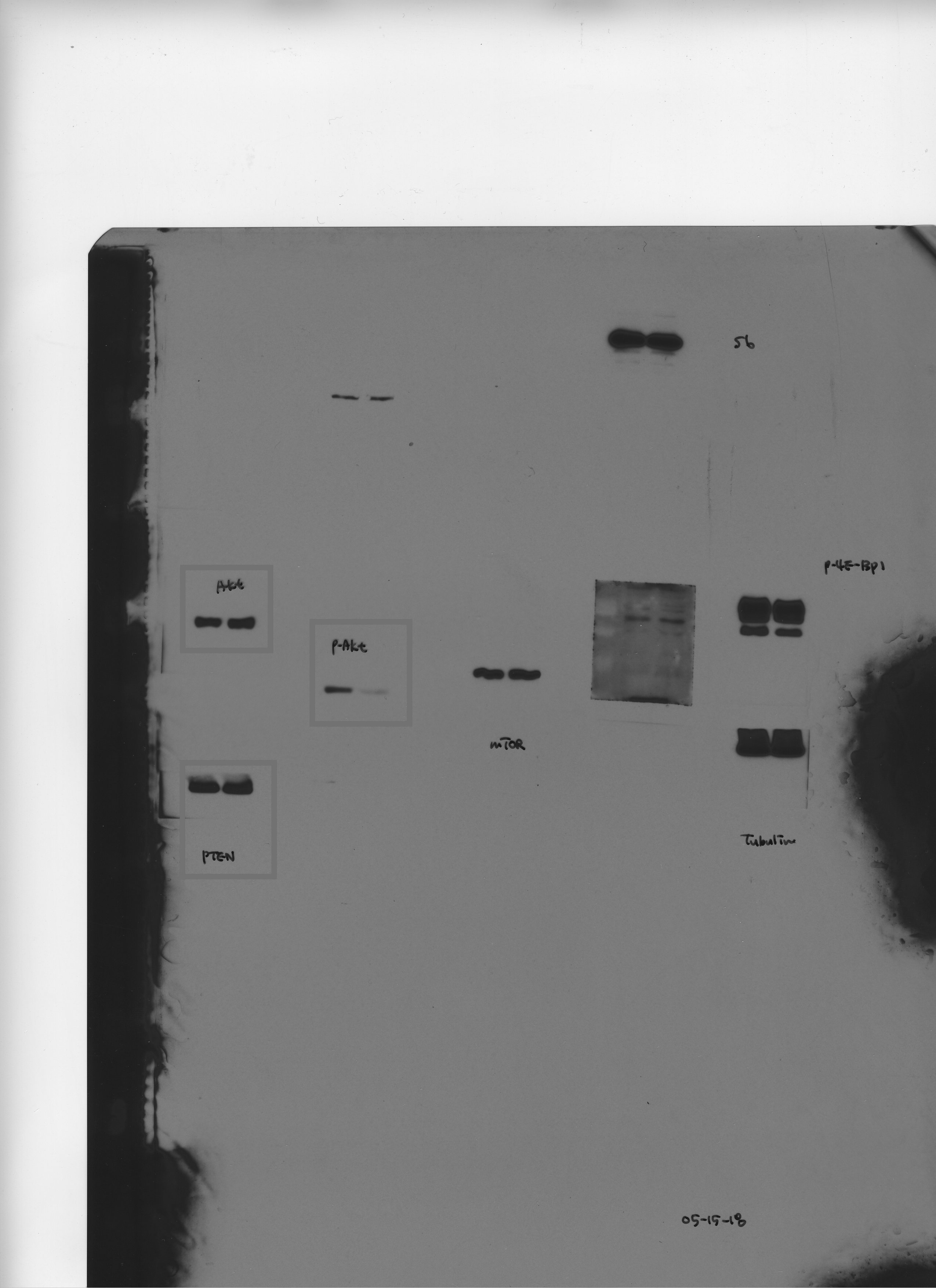

Supplement: Supplementary file 6 — Source data Fig. 2 [file 44318_2024_330_MOESM6_ESM.zip › Figure 2 source data/Figure 2E/Figure 2E pAkt Akt PTEN.jpg]

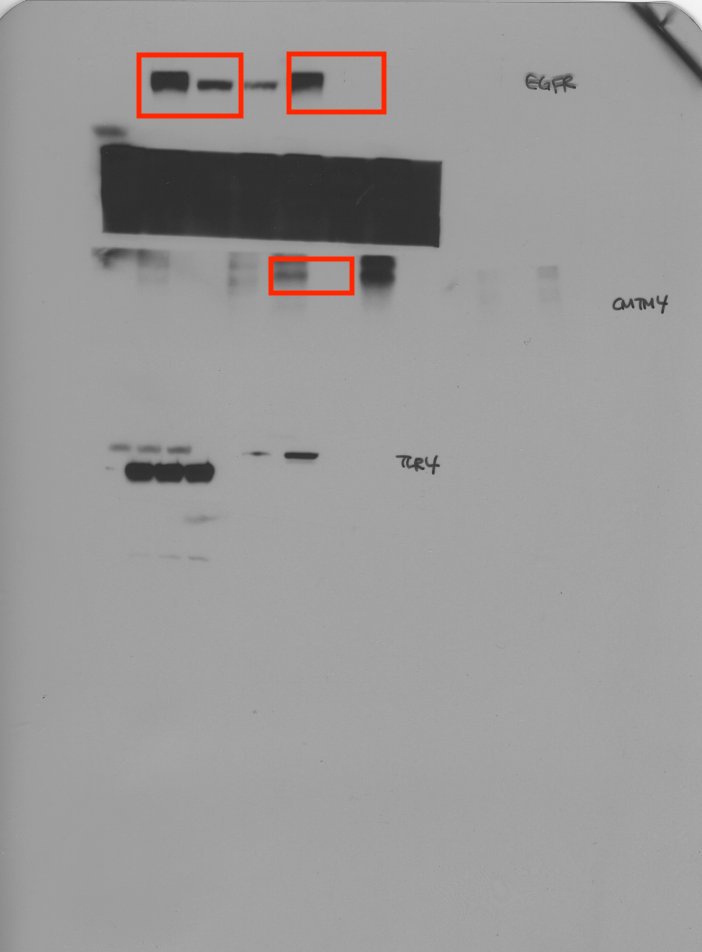

Supplement: Supplementary file 7 — Source data Fig. 3 [file 44318_2024_330_MOESM7_ESM.zip › Figure 3 source data/Figure 3E/Figure 3E left EGFR input&IP, CMTM4 IP.jpg]

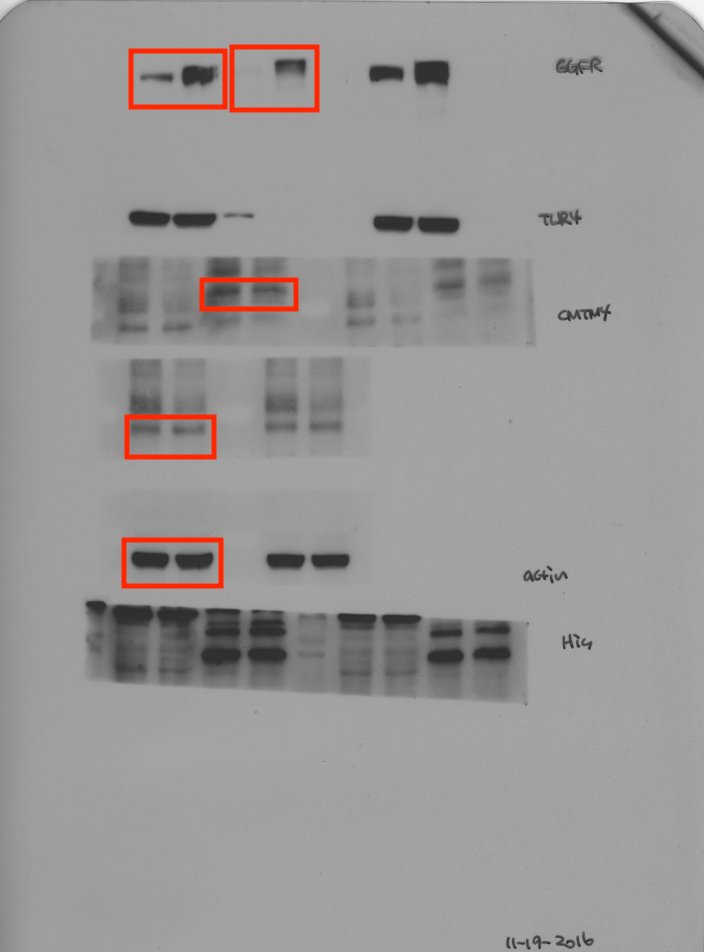

Supplement: Supplementary file 7 — Source data Fig. 3 [file 44318_2024_330_MOESM7_ESM.zip › Figure 3 source data/Figure 3E/Figure 3E right.jpg]

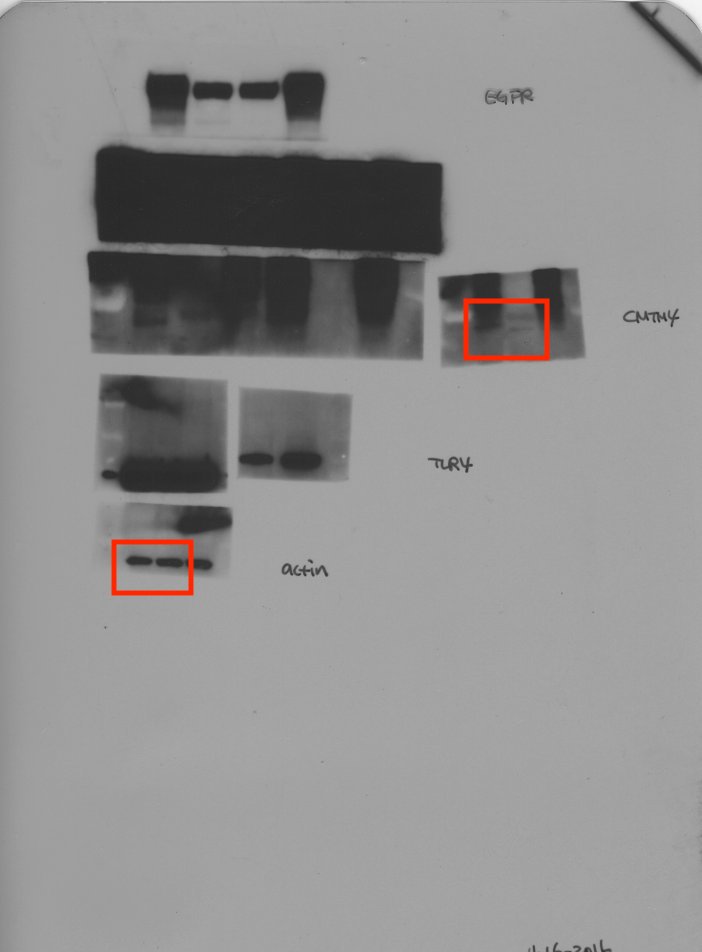

Supplement: Supplementary file 7 — Source data Fig. 3 [file 44318_2024_330_MOESM7_ESM.zip › Figure 3 source data/Figure 3E/Figure 3E left CMTM4 input actin.jpg]

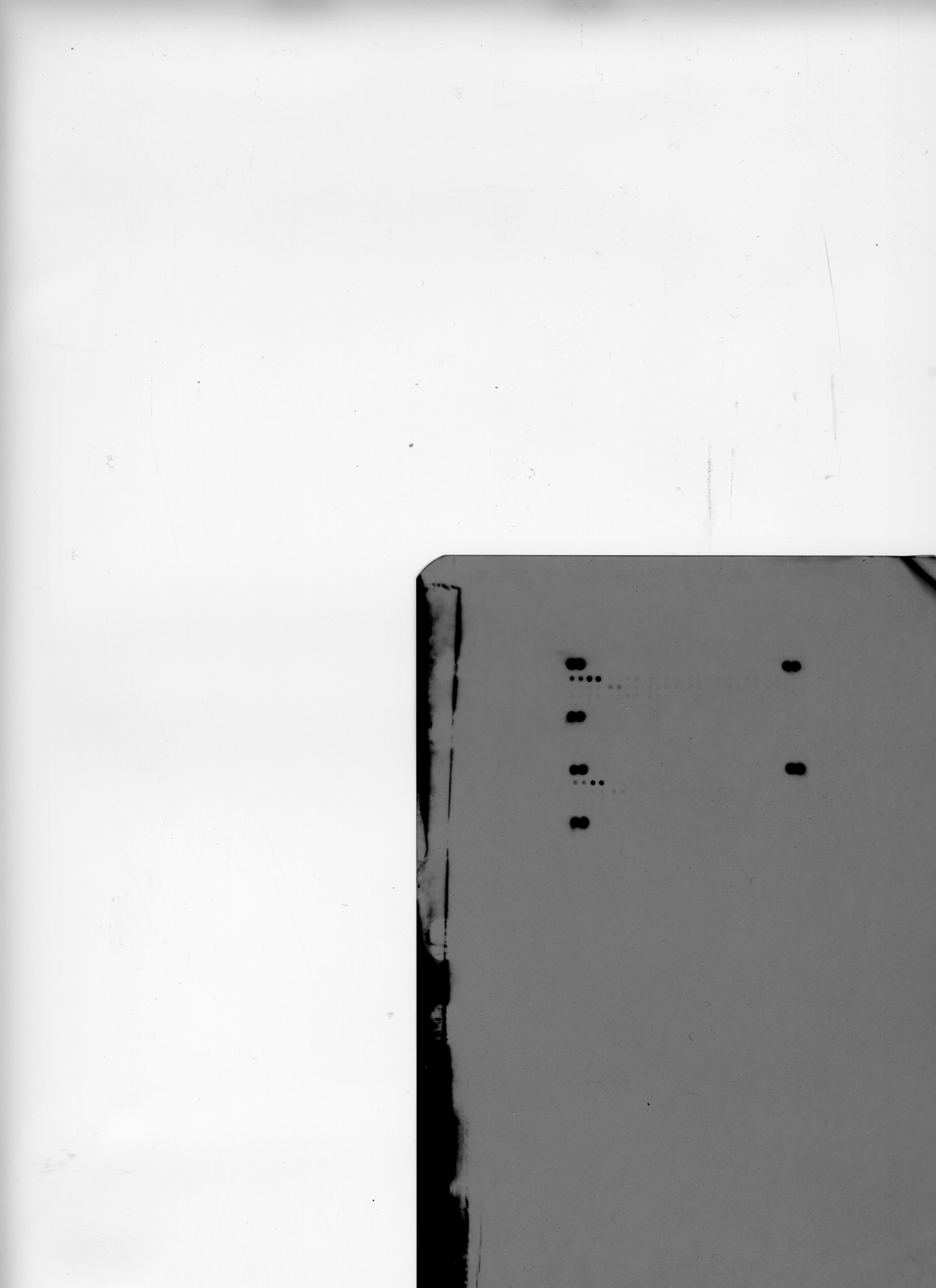

Supplement: Supplementary file 7 — Source data Fig. 3 [file 44318_2024_330_MOESM7_ESM.zip › Figure 3 source data/Figure 3B/Figure 3B.jpg]

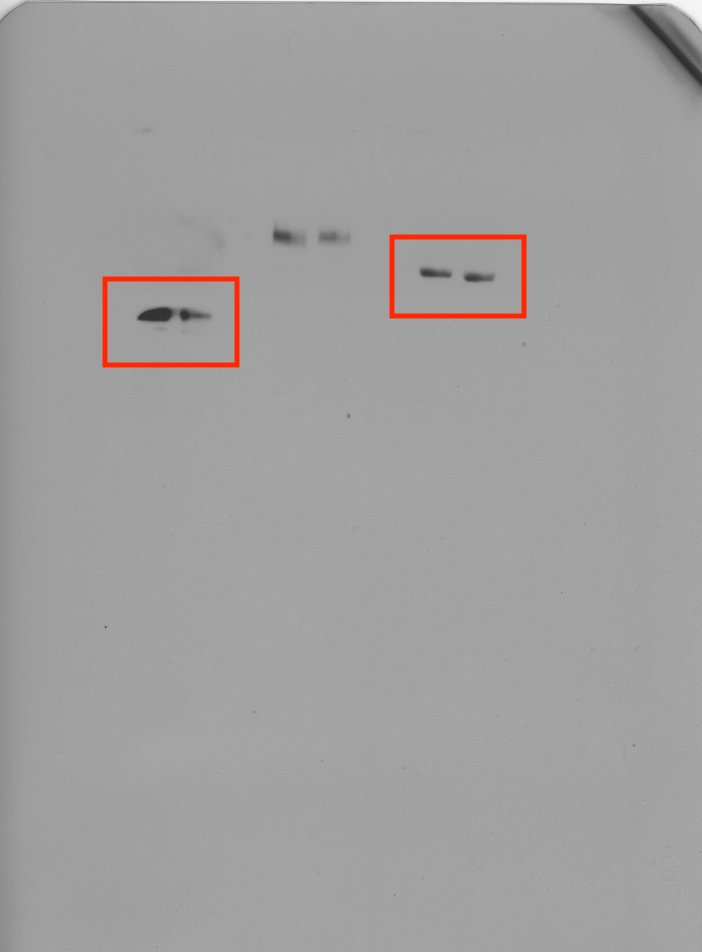

Supplement: Supplementary file 7 — Source data Fig. 3 [file 44318_2024_330_MOESM7_ESM.zip › Figure 3 source data/Figure 3C/Figure 3C CMTM4 actin.jpg]

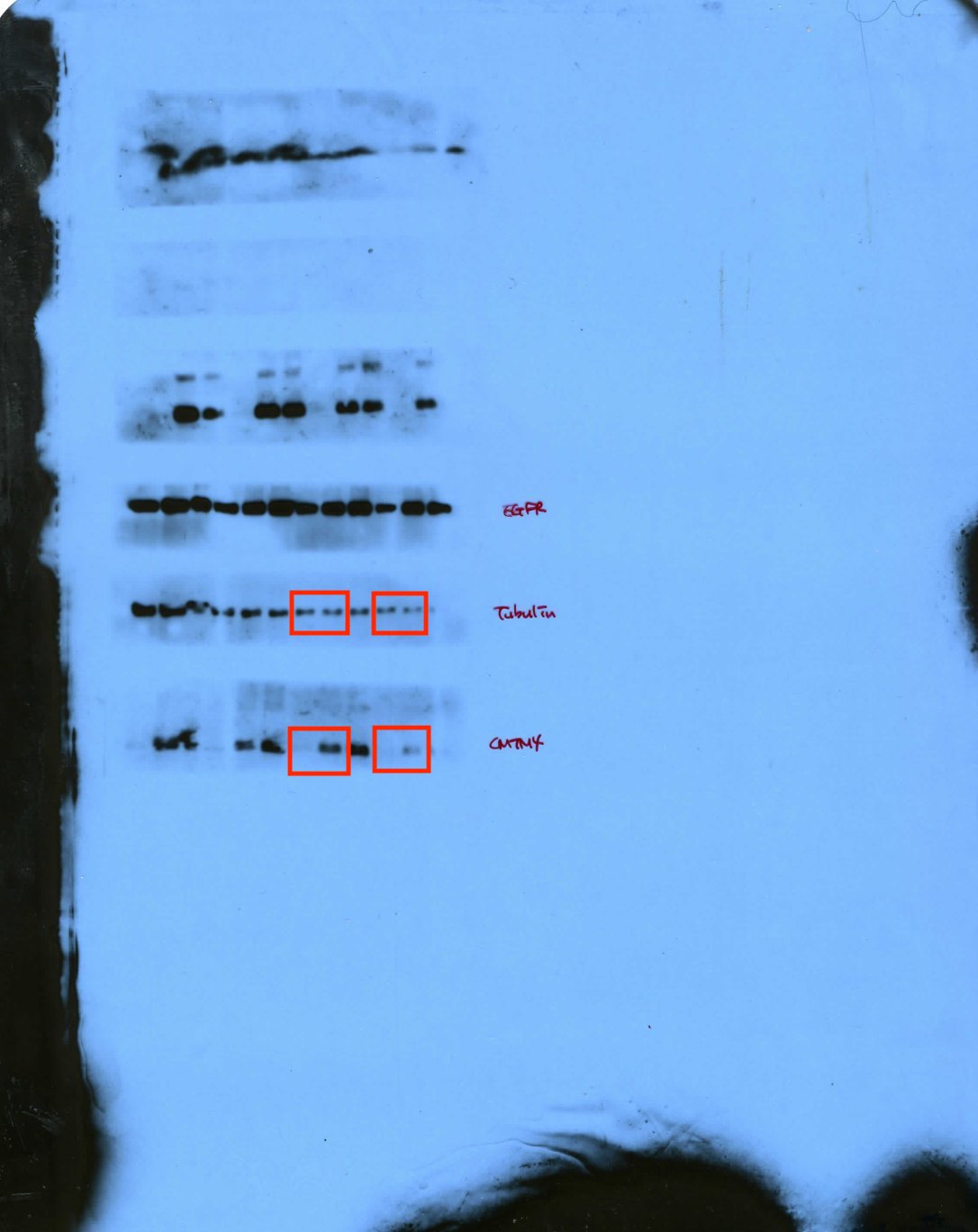

Supplement: Supplementary file 7 — Source data Fig. 3 [file 44318_2024_330_MOESM7_ESM.zip › Figure 3 source data/Figure 3D/Figure 3D CMTM4 Tubulin.jpg]

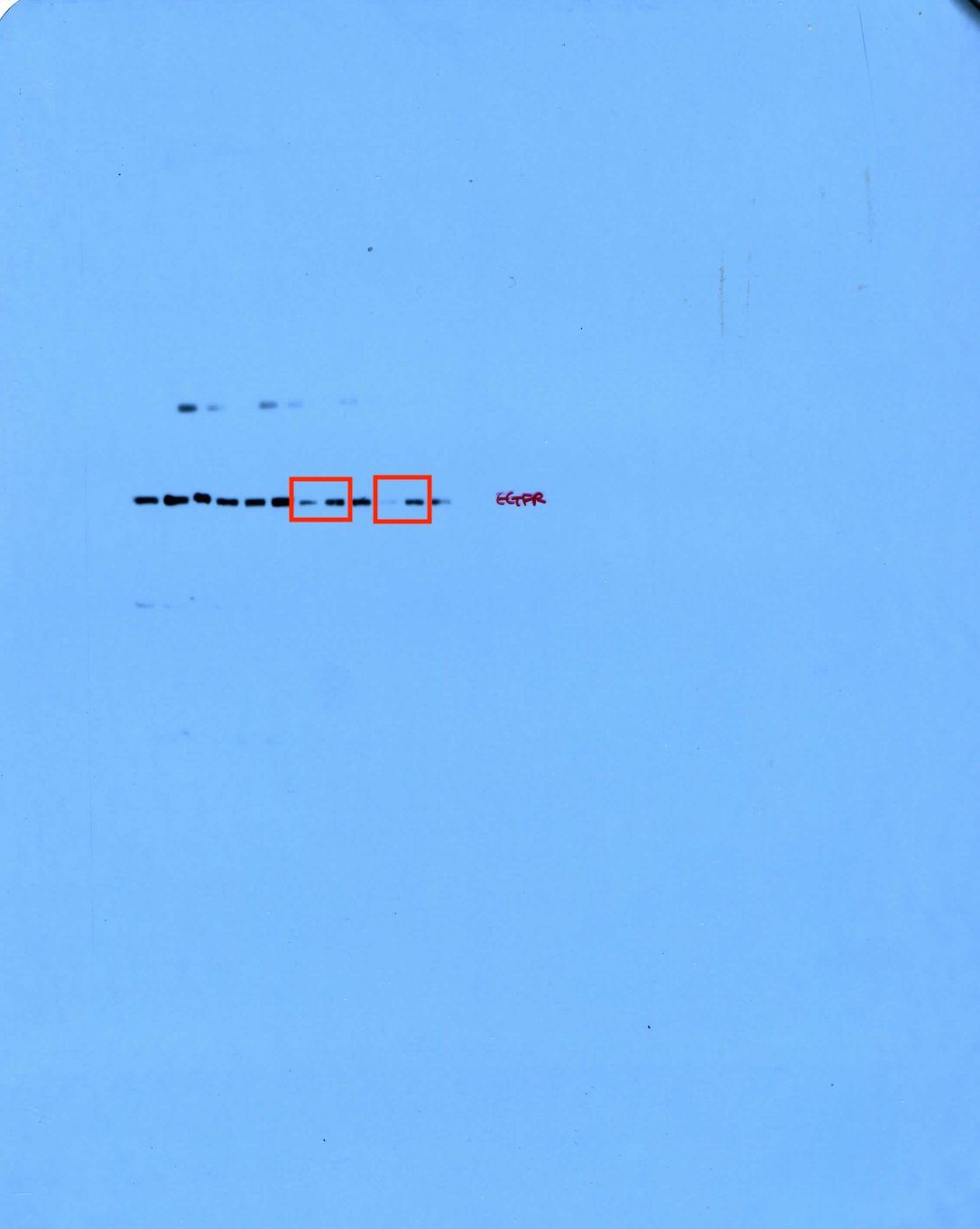

Supplement: Supplementary file 7 — Source data Fig. 3 [file 44318_2024_330_MOESM7_ESM.zip › Figure 3 source data/Figure 3D/Figure 3D EGFR.jpg]

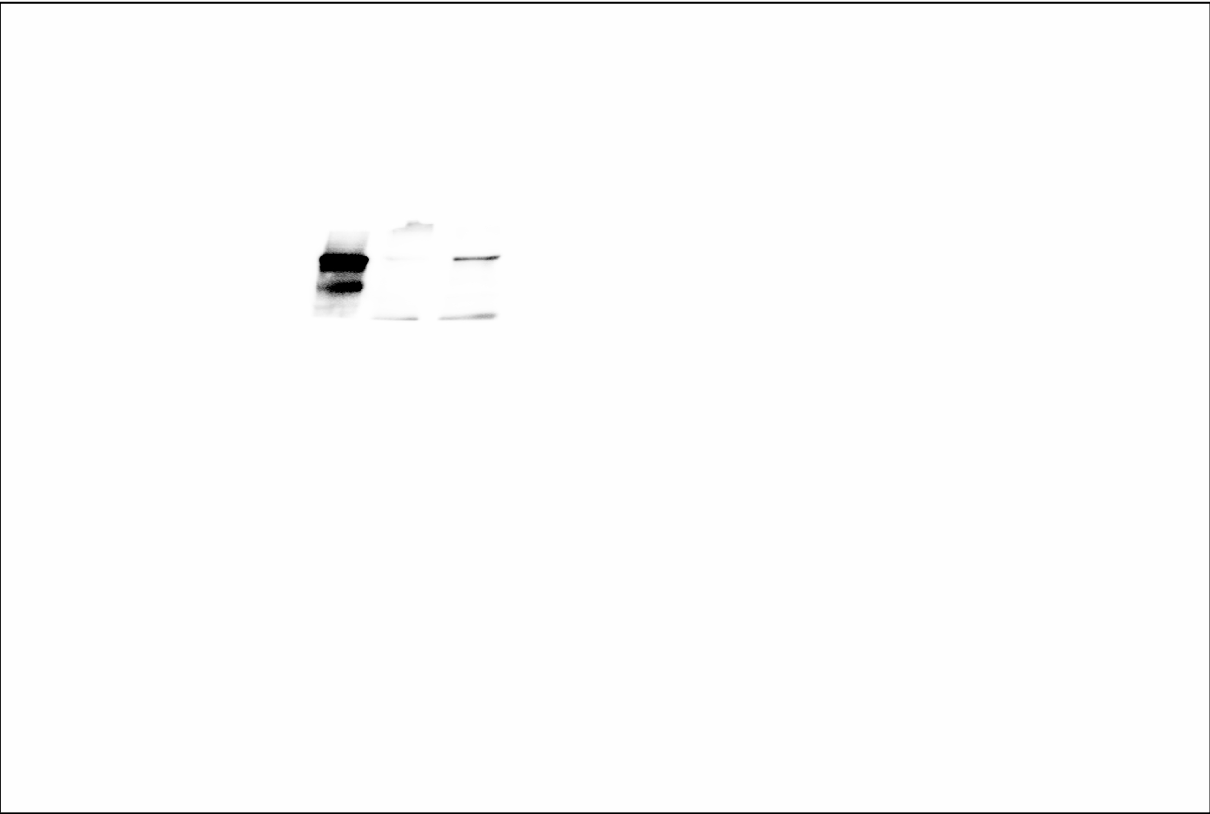

Supplement: Supplementary file 7 — Source data Fig. 3 [file 44318_2024_330_MOESM7_ESM.zip › Figure 3 source data/Figure 3F/Figure 3F 4T1 EGFR.png]

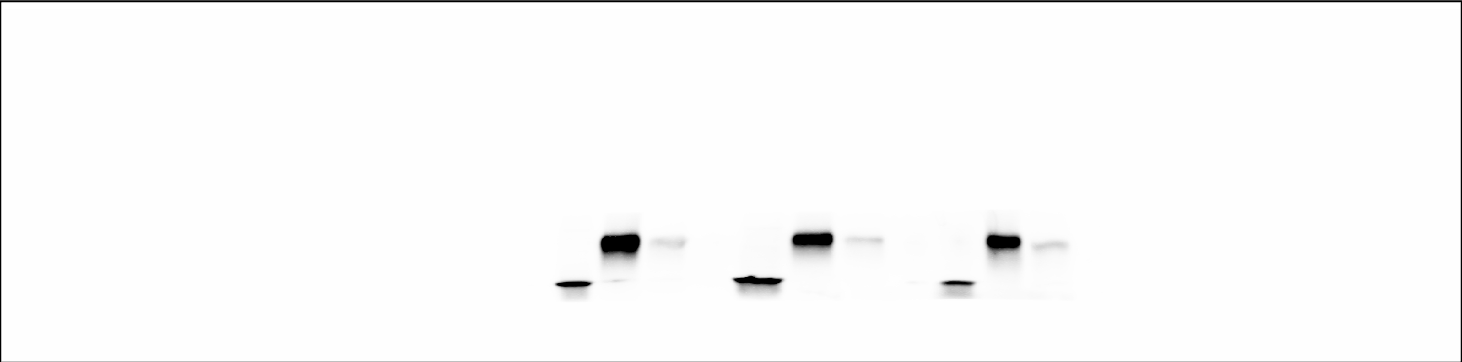

Supplement: Supplementary file 7 — Source data Fig. 3 [file 44318_2024_330_MOESM7_ESM.zip › Figure 3 source data/Figure 3F/Figure 3F HCC827 H292 4T1 GAPDH.png]

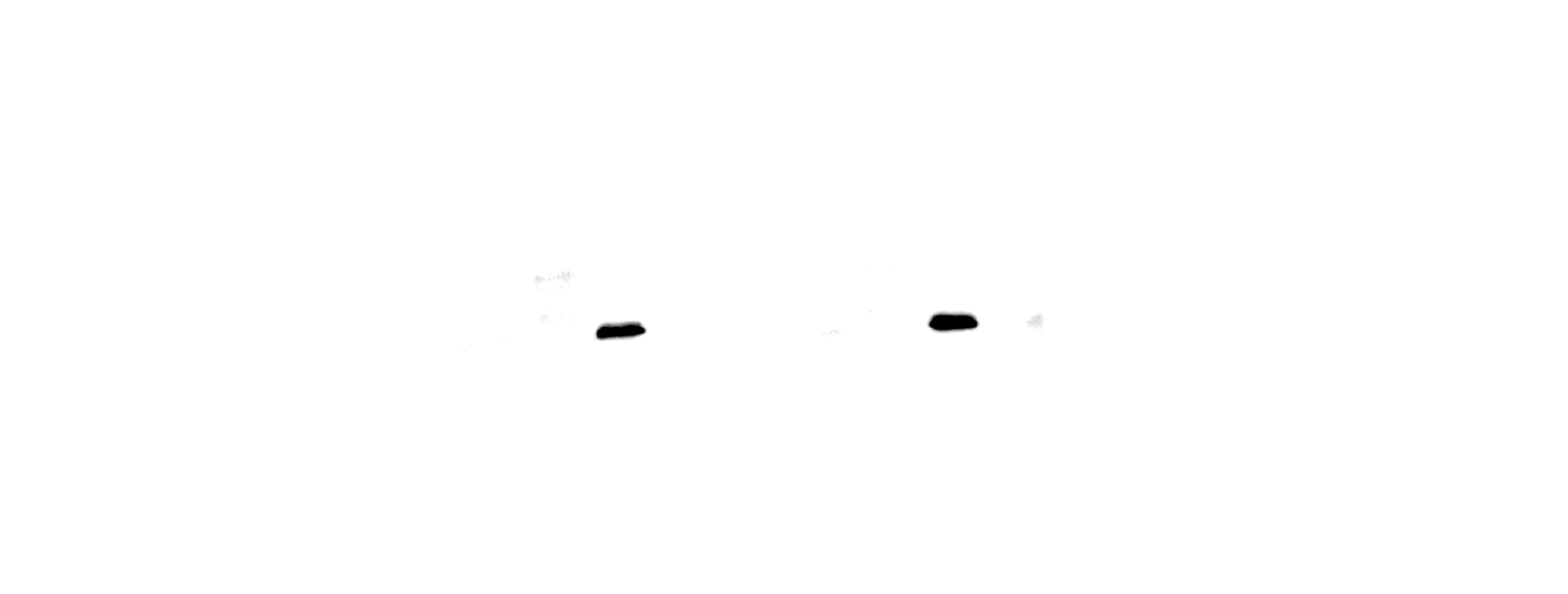

Supplement: Supplementary file 7 — Source data Fig. 3 [file 44318_2024_330_MOESM7_ESM.zip › Figure 3 source data/Figure 3F/Figure 3F LLC CMTM4.png]

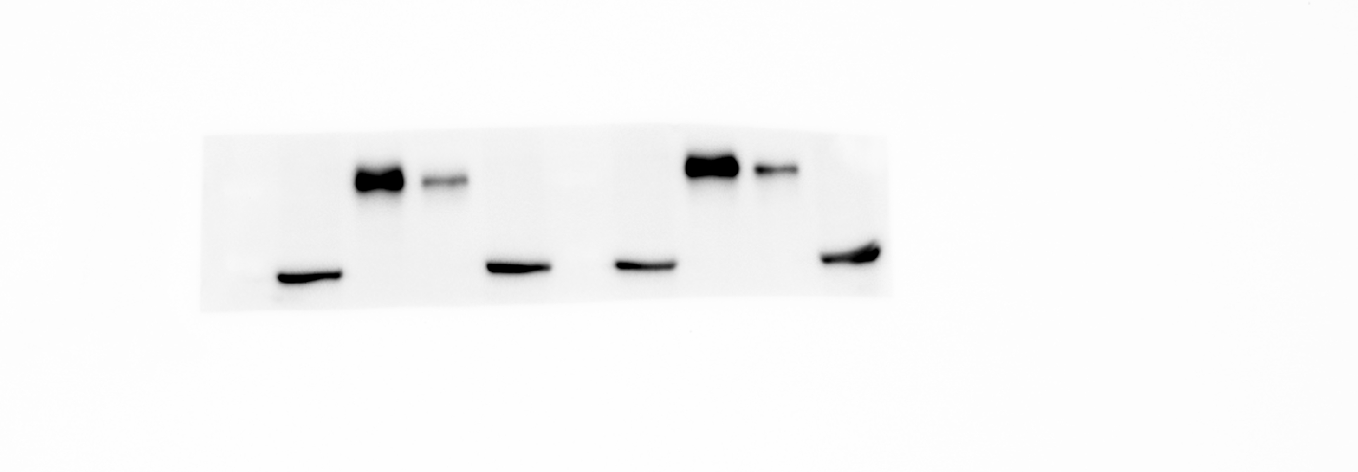

Supplement: Supplementary file 7 — Source data Fig. 3 [file 44318_2024_330_MOESM7_ESM.zip › Figure 3 source data/Figure 3F/Figure 3F LLC GAPDH.png]

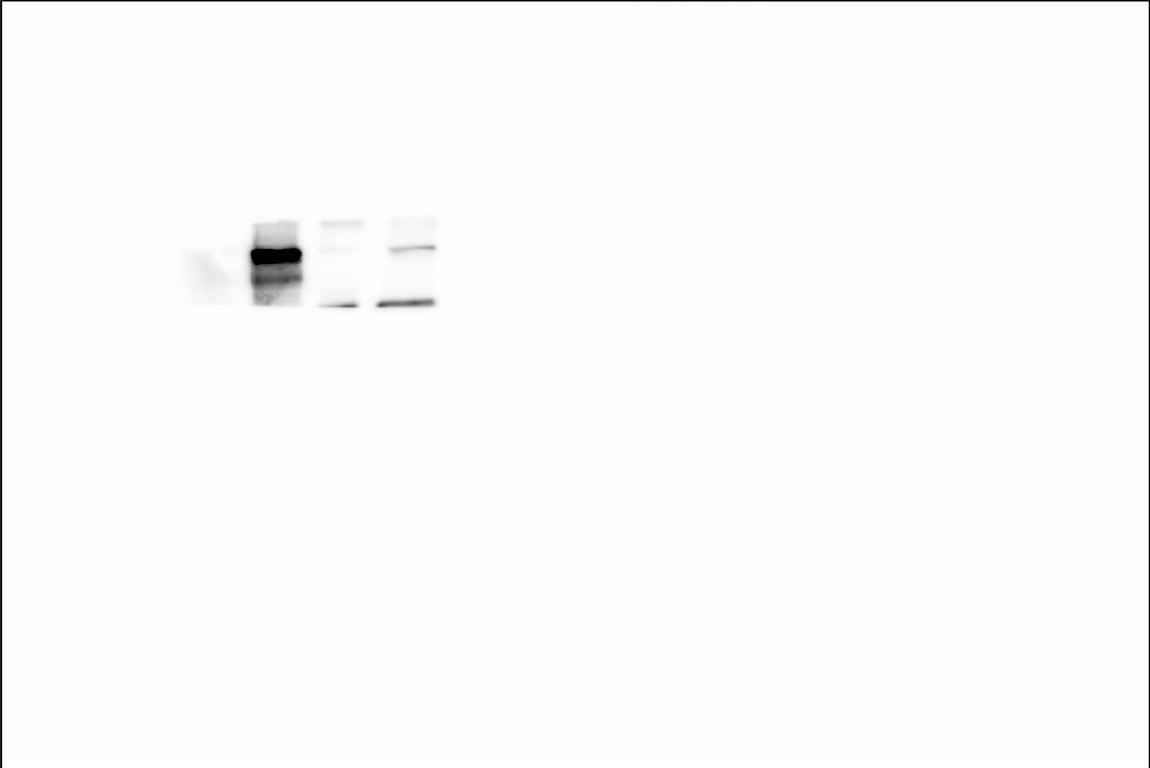

Supplement: Supplementary file 7 — Source data Fig. 3 [file 44318_2024_330_MOESM7_ESM.zip › Figure 3 source data/Figure 3F/Figure 3F HCC827 EGFR.png]

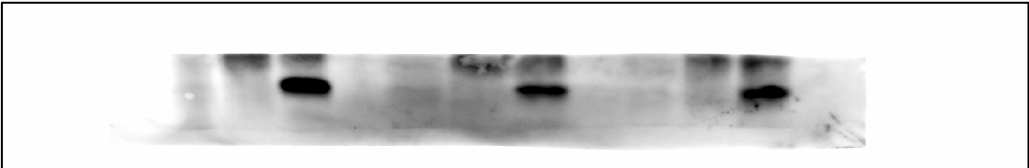

Supplement: Supplementary file 7 — Source data Fig. 3 [file 44318_2024_330_MOESM7_ESM.zip › Figure 3 source data/Figure 3F/Figure 3F HCC827 H292 4T1 CMTM4.png]

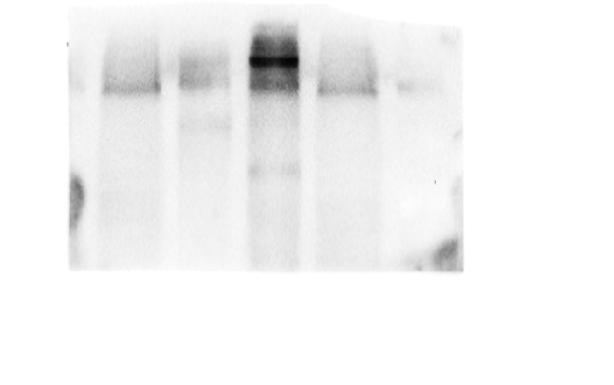

Supplement: Supplementary file 7 — Source data Fig. 3 [file 44318_2024_330_MOESM7_ESM.zip › Figure 3 source data/Figure 3F/Figure 3F LLC EGFR.png]

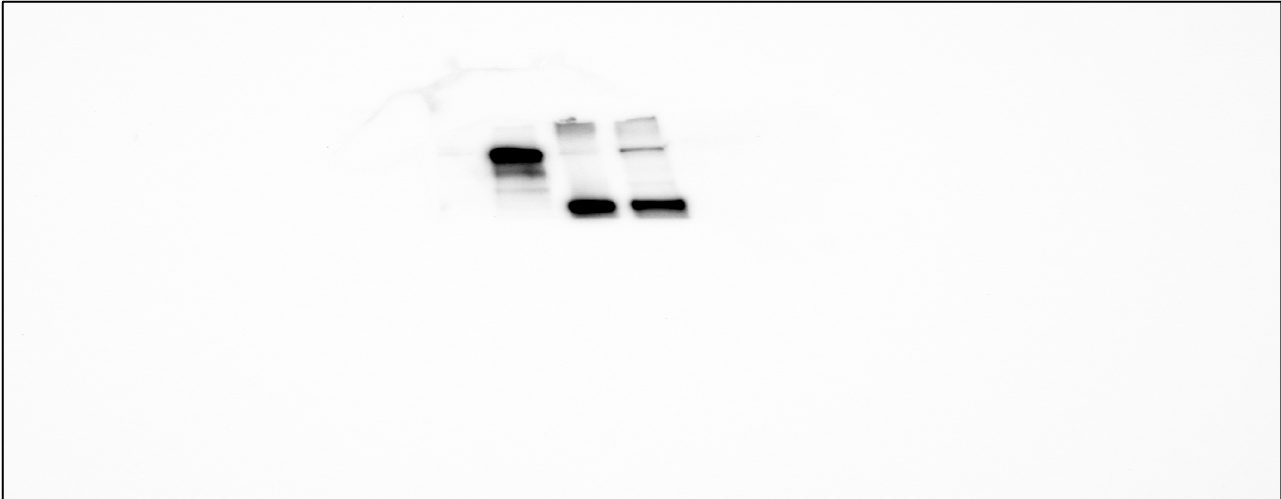

Supplement: Supplementary file 7 — Source data Fig. 3 [file 44318_2024_330_MOESM7_ESM.zip › Figure 3 source data/Figure 3F/Figure 3F H292 EGFR.png]

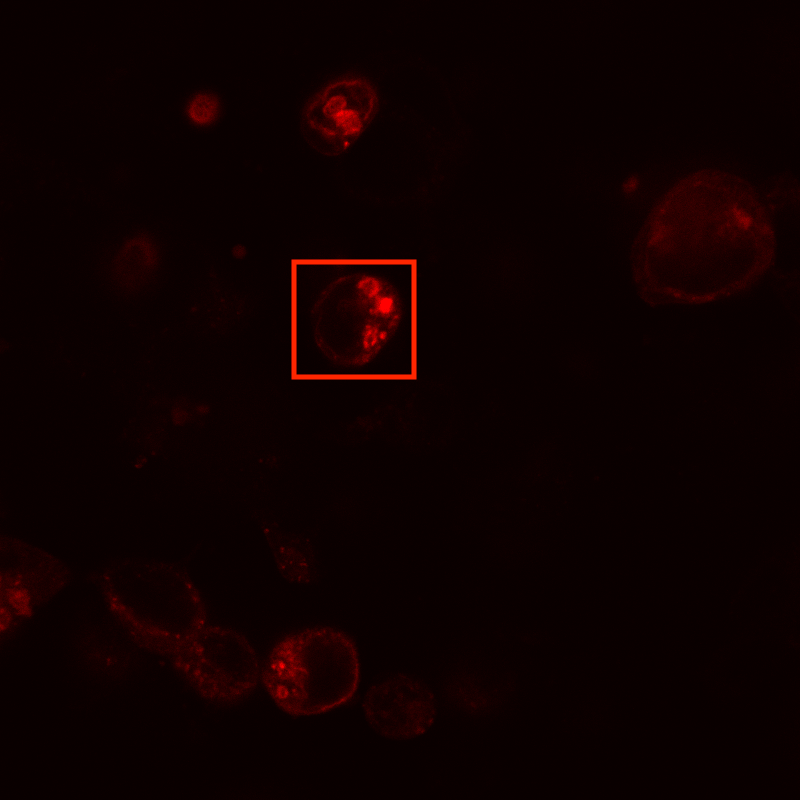

Supplement: Supplementary file 8 — Source data Fig. 4 [file 44318_2024_330_MOESM8_ESM.zip › Figure 4 source data/Figure 4E/Figure 4E +EGF Rab5.tif]

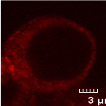

Supplement: Supplementary file 8 — Source data Fig. 4 [file 44318_2024_330_MOESM8_ESM.zip › Figure 4 source data/Figure 4E/Figure 4E -EGF Rab5.png]

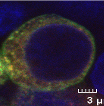

Supplement: Supplementary file 8 — Source data Fig. 4 [file 44318_2024_330_MOESM8_ESM.zip › Figure 4 source data/Figure 4E/Figure 4E -EGF Merged.png]

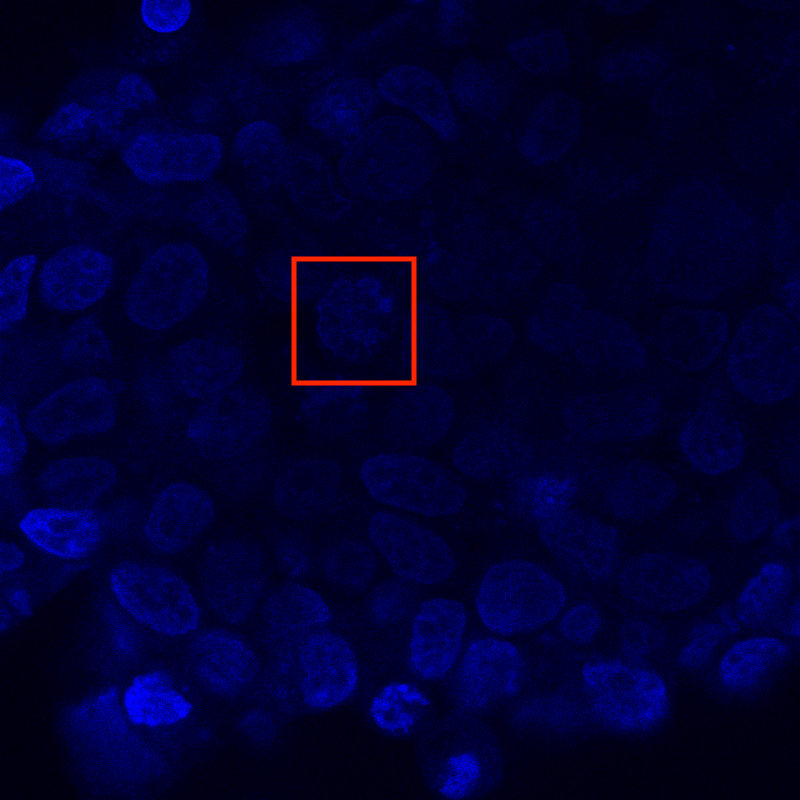

Supplement: Supplementary file 8 — Source data Fig. 4 [file 44318_2024_330_MOESM8_ESM.zip › Figure 4 source data/Figure 4E/Figure 4E +EGF DAPI.tif]

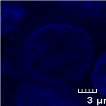

Supplement: Supplementary file 8 — Source data Fig. 4 [file 44318_2024_330_MOESM8_ESM.zip › Figure 4 source data/Figure 4E/Figure 4E -EGF DAPI.png]

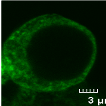

Supplement: Supplementary file 8 — Source data Fig. 4 [file 44318_2024_330_MOESM8_ESM.zip › Figure 4 source data/Figure 4E/Figure 4E -EGF CMTM4.png]

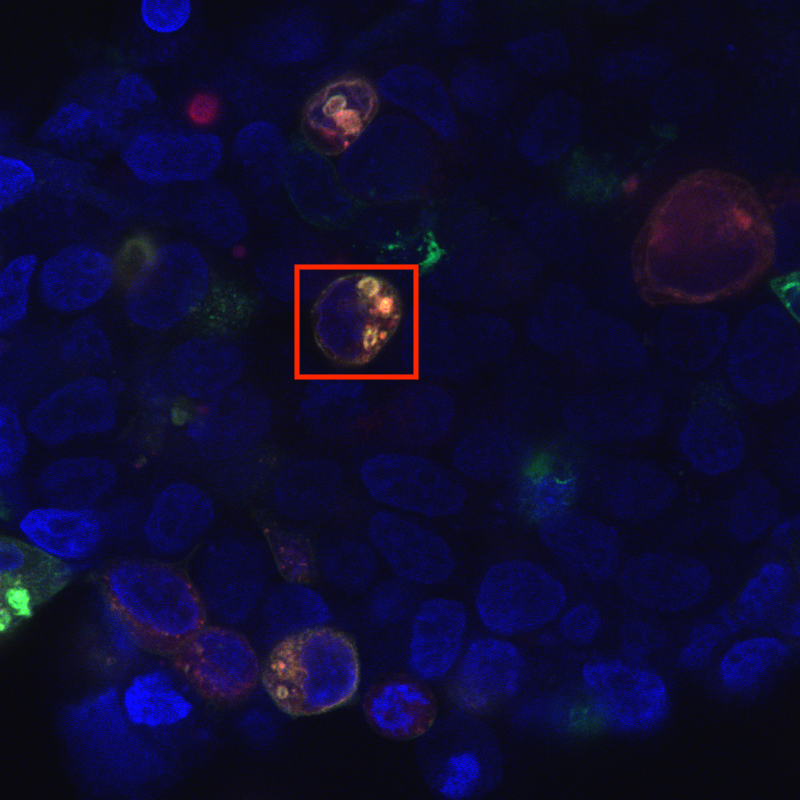

Supplement: Supplementary file 8 — Source data Fig. 4 [file 44318_2024_330_MOESM8_ESM.zip › Figure 4 source data/Figure 4E/Figure 4E +EGF Merged.tif]

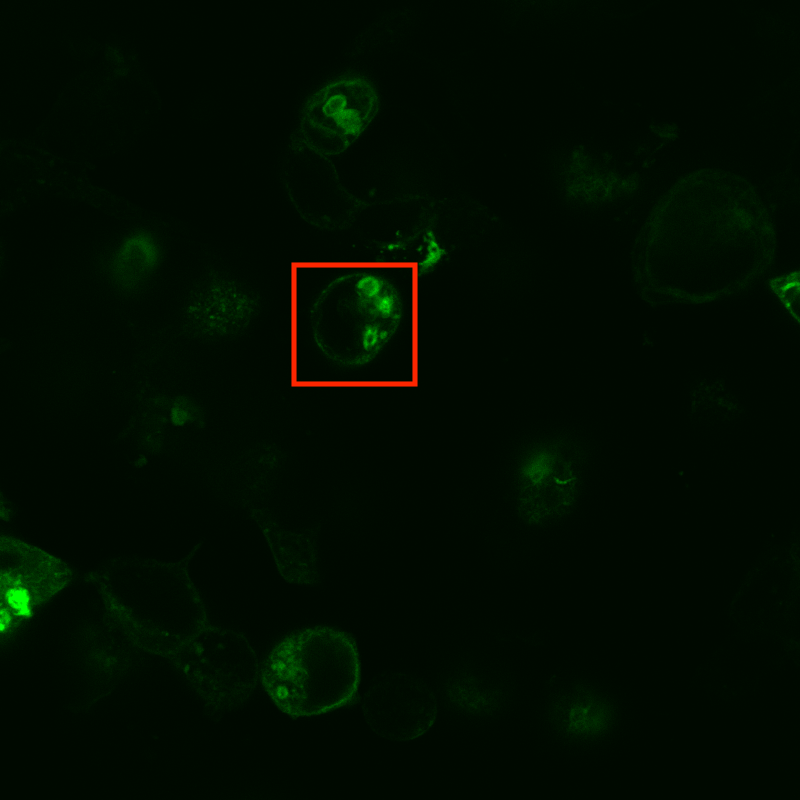

Supplement: Supplementary file 8 — Source data Fig. 4 [file 44318_2024_330_MOESM8_ESM.zip › Figure 4 source data/Figure 4E/Figure 4E +EGF CMTM4.tif]

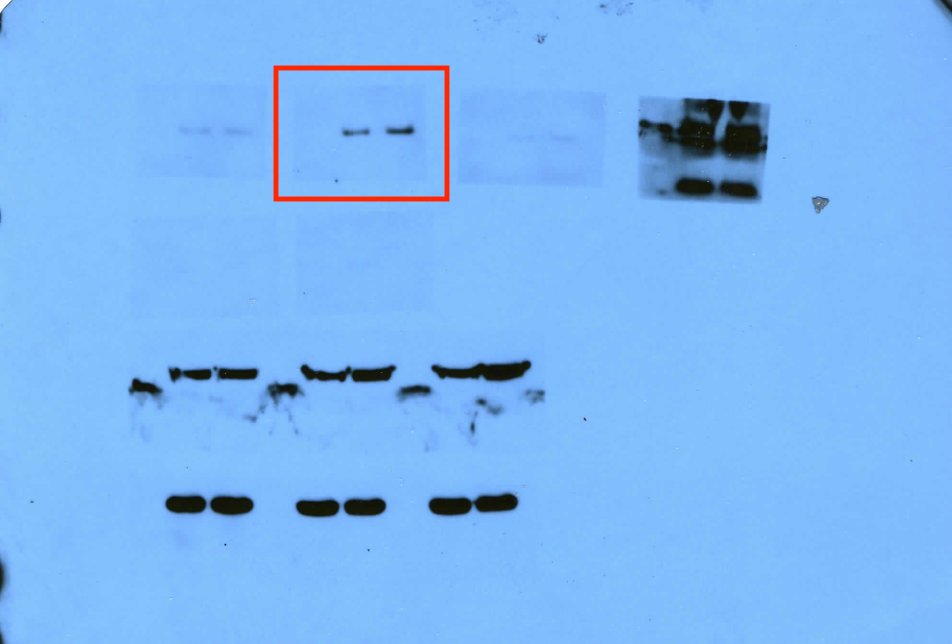

Supplement: Supplementary file 8 — Source data Fig. 4 [file 44318_2024_330_MOESM8_ESM.zip › Figure 4 source data/Figure 4B/Figure 4B Rab5.jpg]

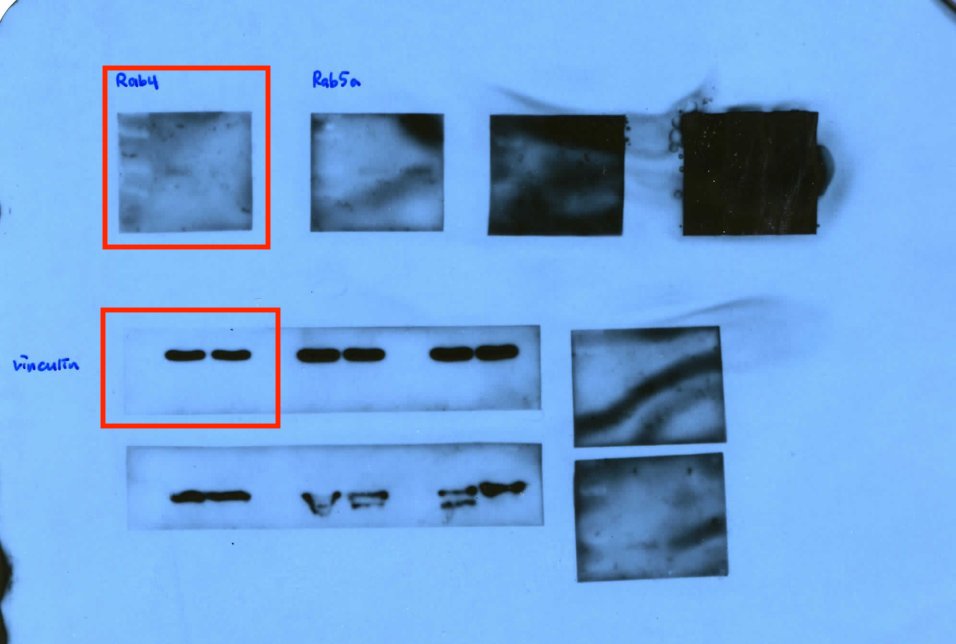

Supplement: Supplementary file 8 — Source data Fig. 4 [file 44318_2024_330_MOESM8_ESM.zip › Figure 4 source data/Figure 4B/Figure 4B Rab4&Vinculin.jpg]

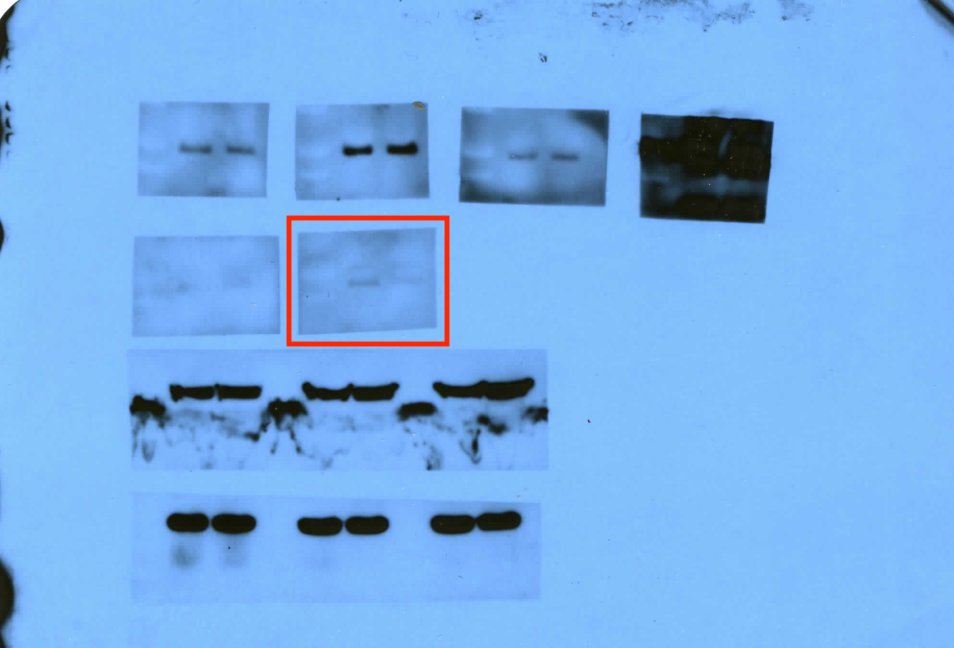

Supplement: Supplementary file 8 — Source data Fig. 4 [file 44318_2024_330_MOESM8_ESM.zip › Figure 4 source data/Figure 4B/Figure 4B CMTM4.jpg]

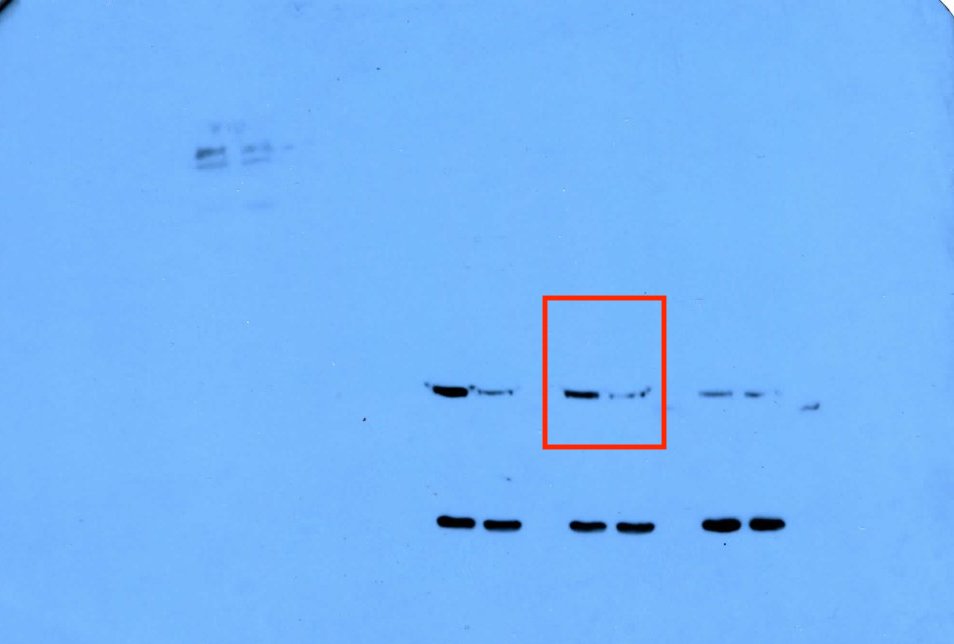

Supplement: Supplementary file 8 — Source data Fig. 4 [file 44318_2024_330_MOESM8_ESM.zip › Figure 4 source data/Figure 4B/Figure 4B Rab11.jpg]

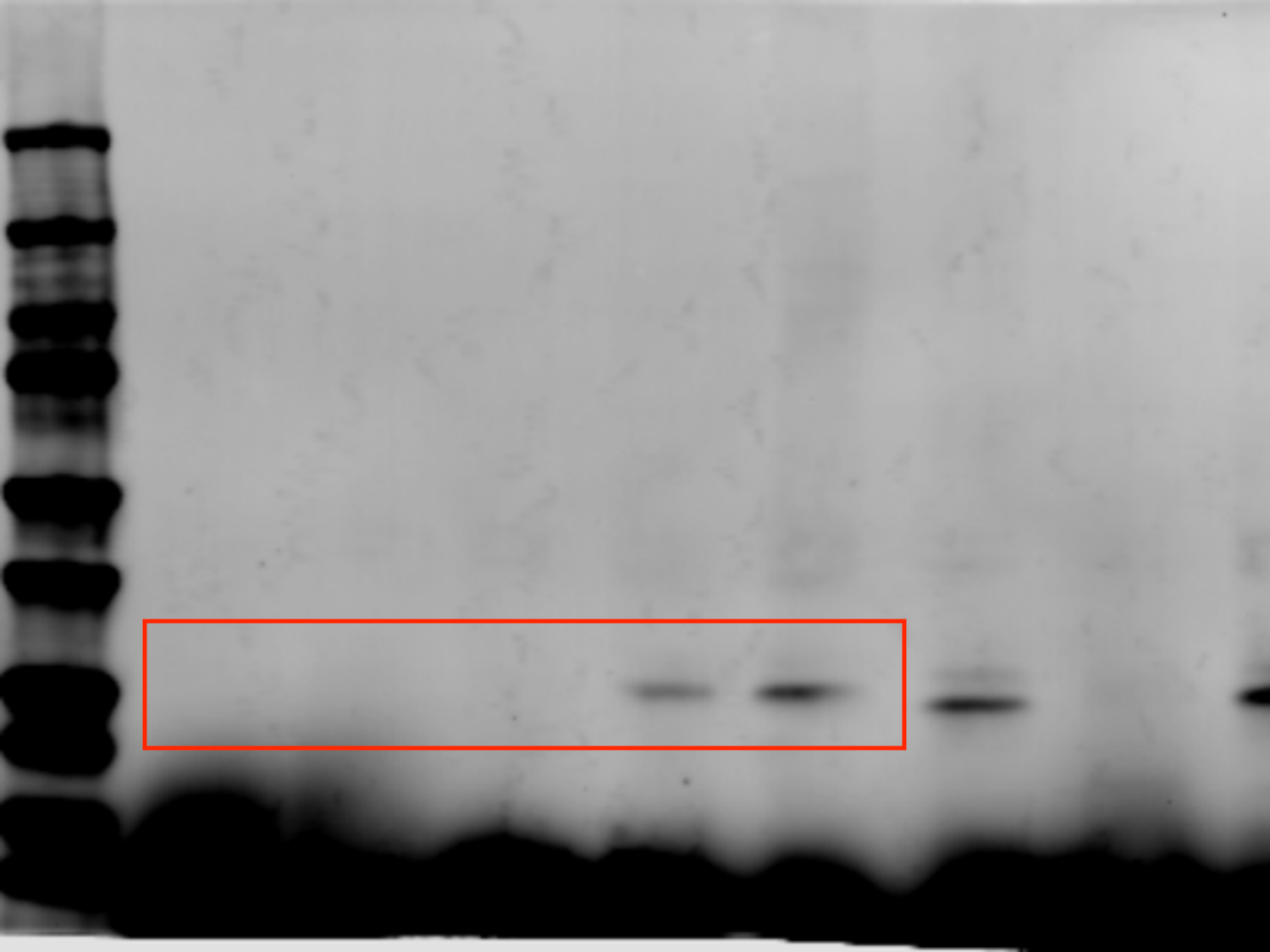

Supplement: Supplementary file 8 — Source data Fig. 4 [file 44318_2024_330_MOESM8_ESM.zip › Figure 4 source data/Figure 4C/Figure 4C.png]

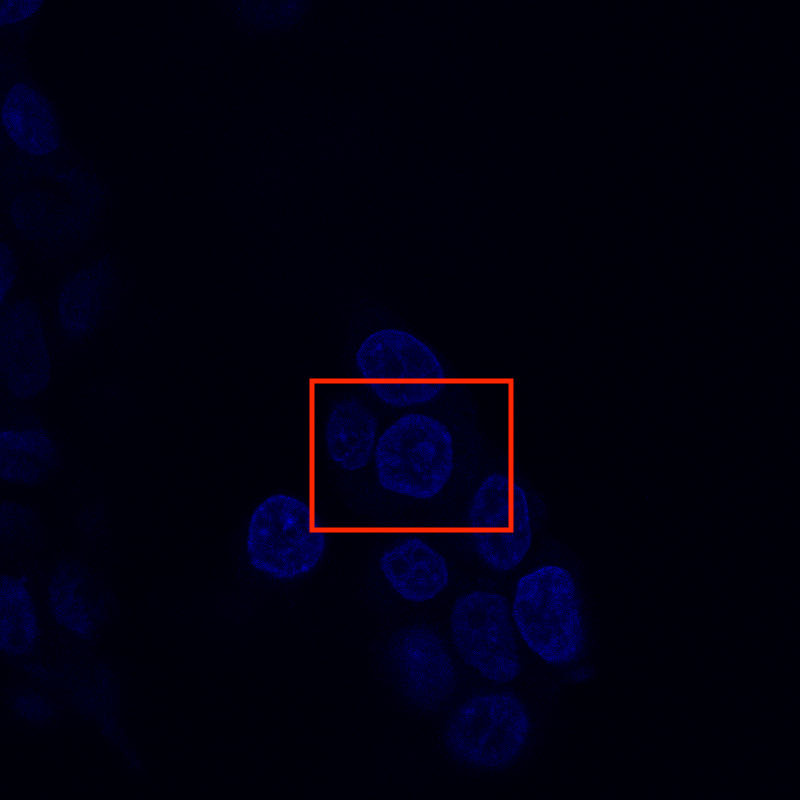

Supplement: Supplementary file 8 — Source data Fig. 4 [file 44318_2024_330_MOESM8_ESM.zip › Figure 4 source data/Figure 4D/Figure 4D -EGF DAPI.tif]

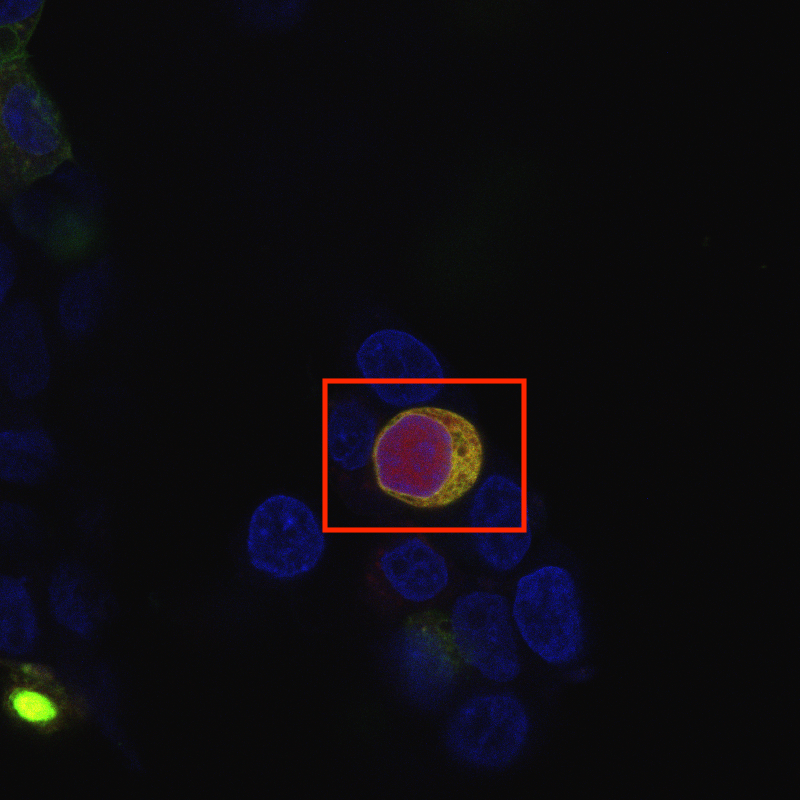

Supplement: Supplementary file 8 — Source data Fig. 4 [file 44318_2024_330_MOESM8_ESM.zip › Figure 4 source data/Figure 4D/Figure 4D -EGF Merged.tif]

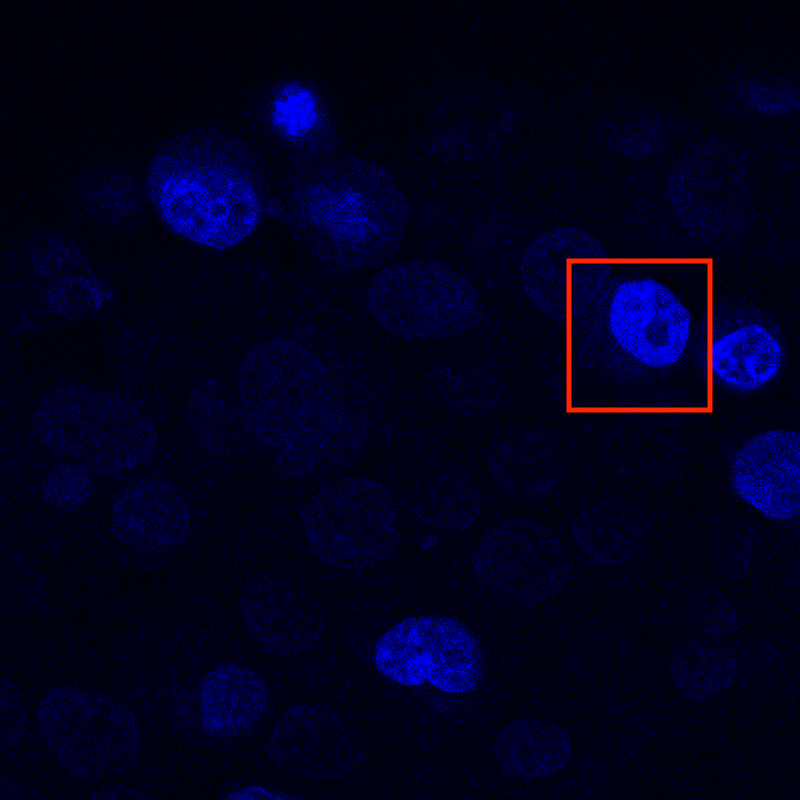

Supplement: Supplementary file 8 — Source data Fig. 4 [file 44318_2024_330_MOESM8_ESM.zip › Figure 4 source data/Figure 4D/Figure 4D +EGF DAPI.tif]

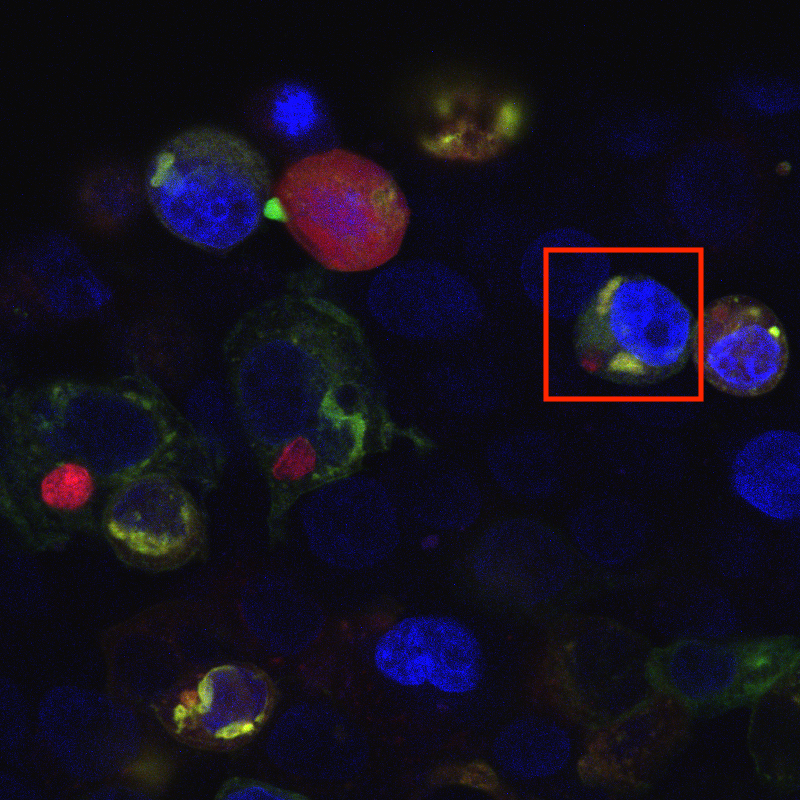

Supplement: Supplementary file 8 — Source data Fig. 4 [file 44318_2024_330_MOESM8_ESM.zip › Figure 4 source data/Figure 4D/Figure 4D +EGF Merged.tif]

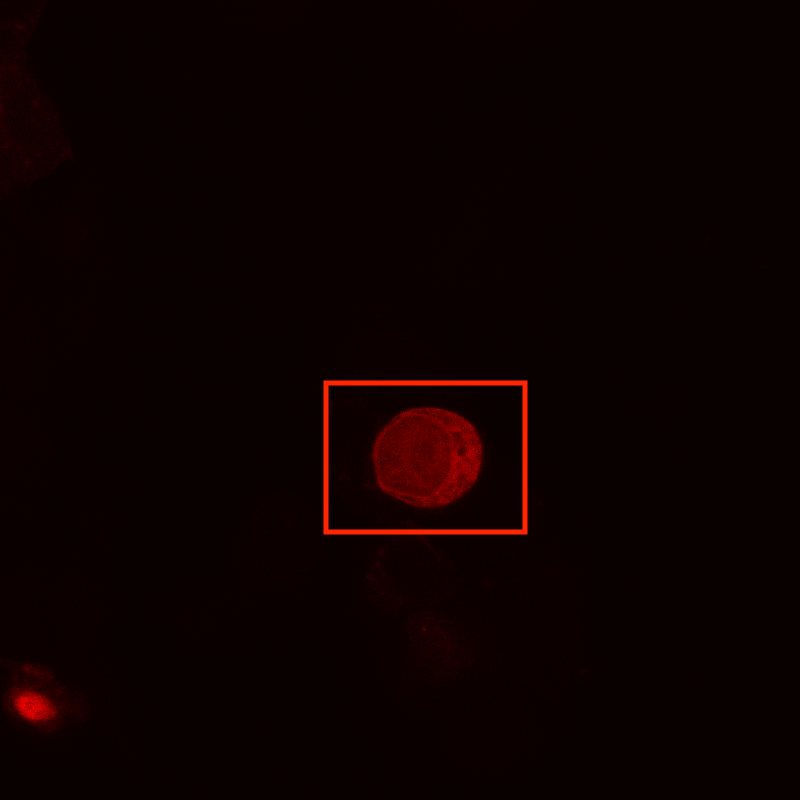

Supplement: Supplementary file 8 — Source data Fig. 4 [file 44318_2024_330_MOESM8_ESM.zip › Figure 4 source data/Figure 4D/Figure 4D -EGF Rab4.tif]

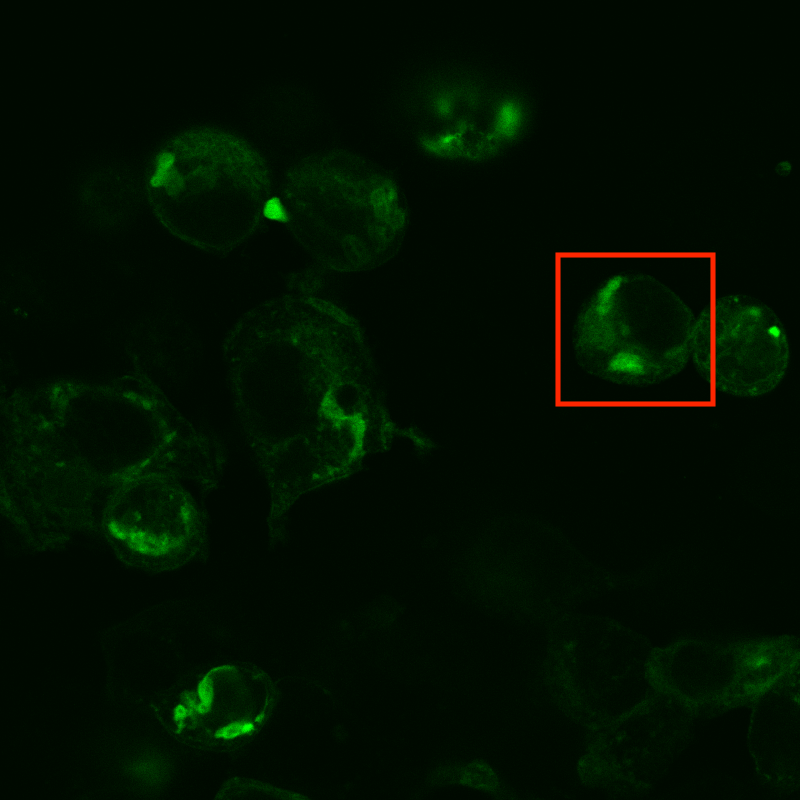

Supplement: Supplementary file 8 — Source data Fig. 4 [file 44318_2024_330_MOESM8_ESM.zip › Figure 4 source data/Figure 4D/Figure 4D +EGF CMTM4.tif]

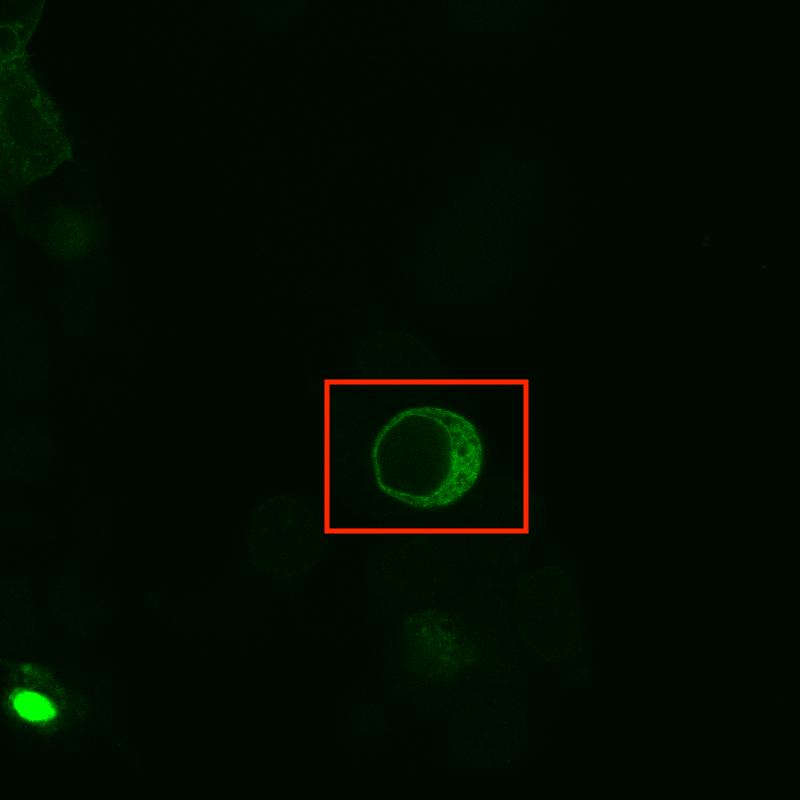

Supplement: Supplementary file 8 — Source data Fig. 4 [file 44318_2024_330_MOESM8_ESM.zip › Figure 4 source data/Figure 4D/Figure 4D -EGF CMTM4.tif]

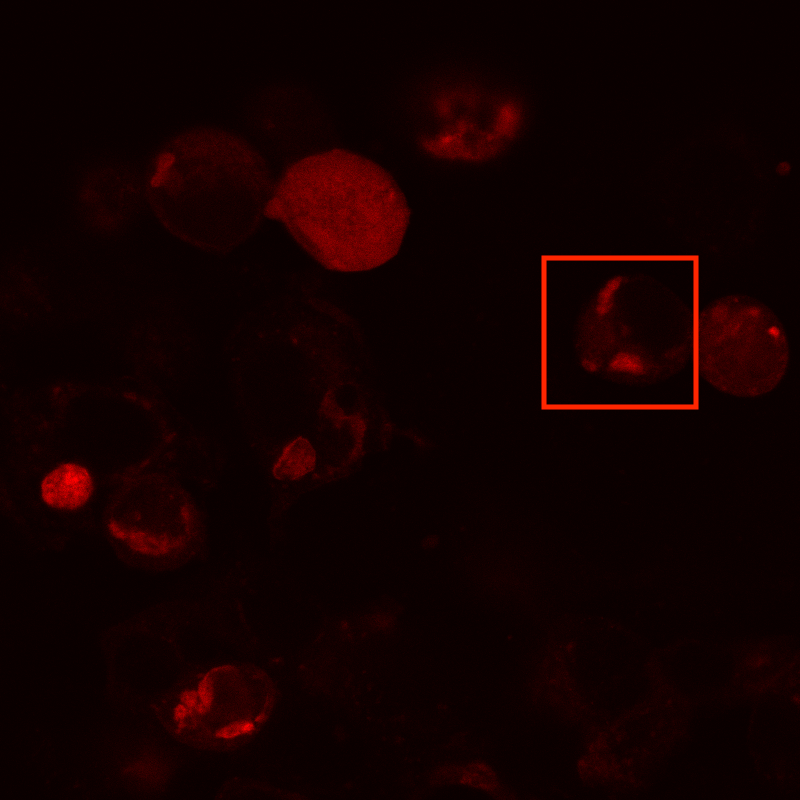

Supplement: Supplementary file 8 — Source data Fig. 4 [file 44318_2024_330_MOESM8_ESM.zip › Figure 4 source data/Figure 4D/Figure 4D +EGF Rab4.tif]

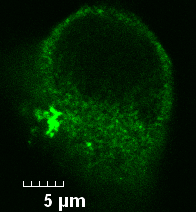

Supplement: Supplementary file 8 — Source data Fig. 4 [file 44318_2024_330_MOESM8_ESM.zip › Figure 4 source data/Figure 4A/Figure 4A +EGF EGFR.tif]

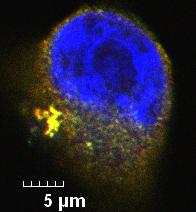

Supplement: Supplementary file 8 — Source data Fig. 4 [file 44318_2024_330_MOESM8_ESM.zip › Figure 4 source data/Figure 4A/Figure 4A +EGF Merged.tif]

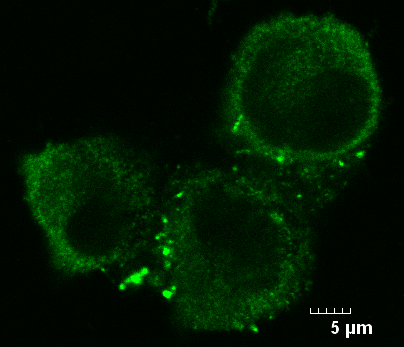

Supplement: Supplementary file 8 — Source data Fig. 4 [file 44318_2024_330_MOESM8_ESM.zip › Figure 4 source data/Figure 4A/Figure 4A -EGF EGFR.tif]

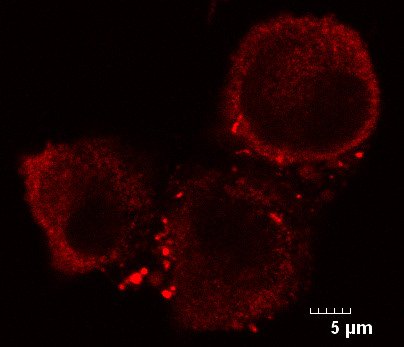

Supplement: Supplementary file 8 — Source data Fig. 4 [file 44318_2024_330_MOESM8_ESM.zip › Figure 4 source data/Figure 4A/Figure 4A -EGF CMTM4.tif]

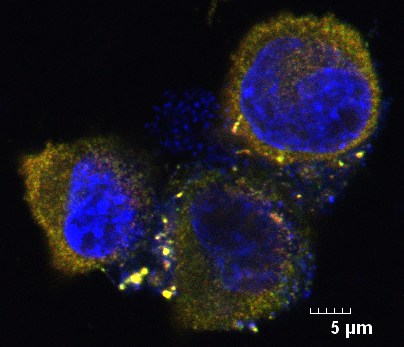

Supplement: Supplementary file 8 — Source data Fig. 4 [file 44318_2024_330_MOESM8_ESM.zip › Figure 4 source data/Figure 4A/Figure 4A -EGF Merged.tif]

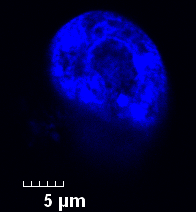

Supplement: Supplementary file 8 — Source data Fig. 4 [file 44318_2024_330_MOESM8_ESM.zip › Figure 4 source data/Figure 4A/Figure 4A +EGF DAPI.tif]

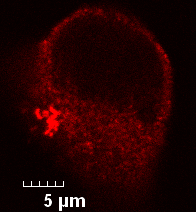

Supplement: Supplementary file 8 — Source data Fig. 4 [file 44318_2024_330_MOESM8_ESM.zip › Figure 4 source data/Figure 4A/Figure 4A +EGF CMTM4.tif]

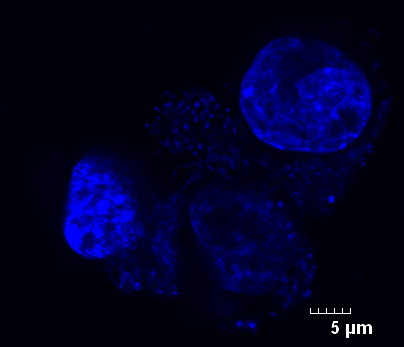

Supplement: Supplementary file 8 — Source data Fig. 4 [file 44318_2024_330_MOESM8_ESM.zip › Figure 4 source data/Figure 4A/Figure 4A -EGF DAPI.tif]

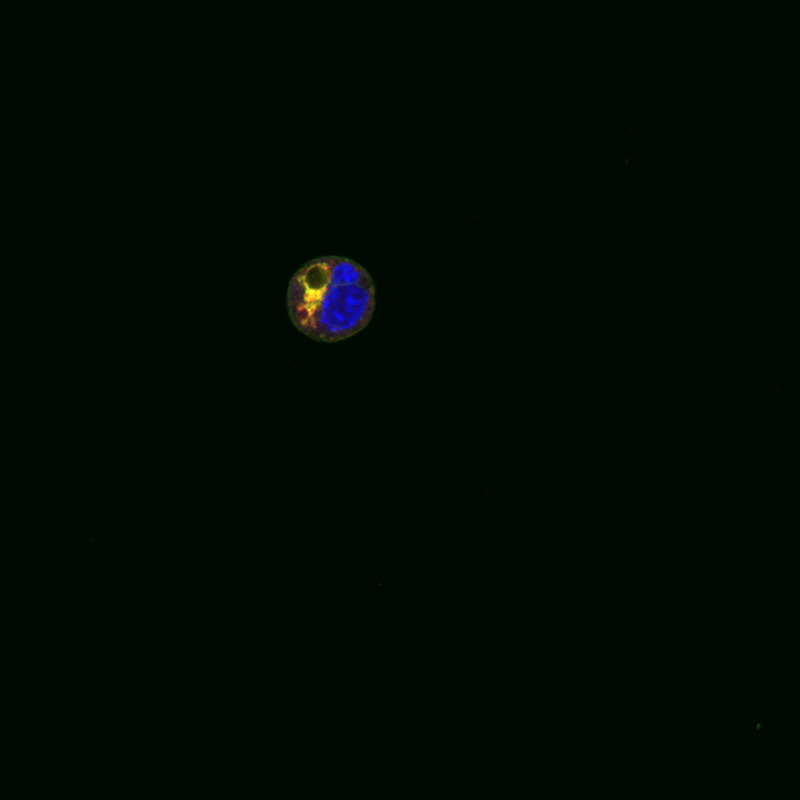

Supplement: Supplementary file 8 — Source data Fig. 4 [file 44318_2024_330_MOESM8_ESM.zip › Figure 4 source data/Figure 4F/Figure 4F +EGF Merged.tif]

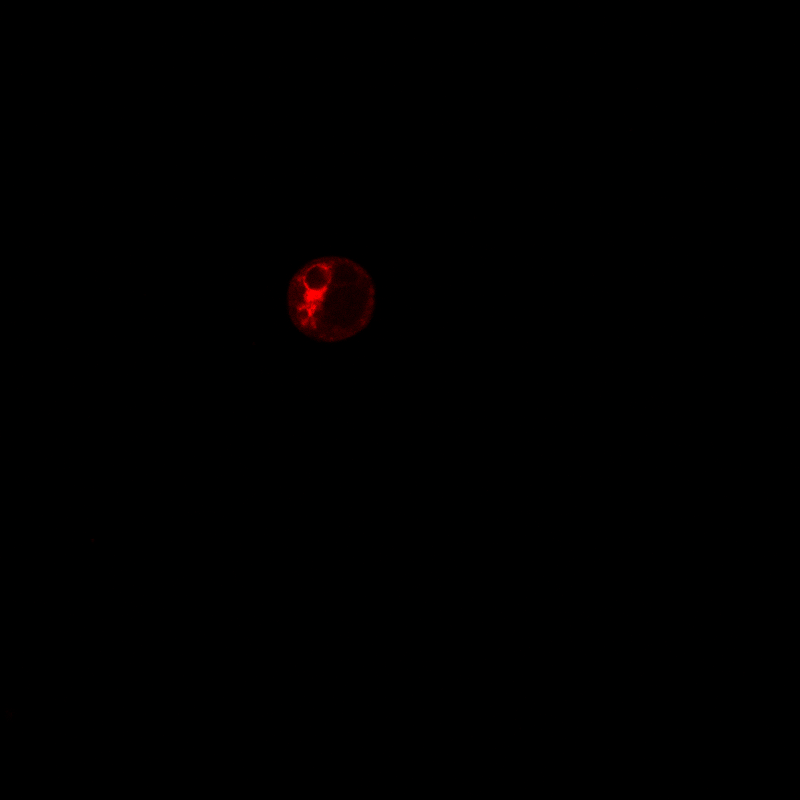

Supplement: Supplementary file 8 — Source data Fig. 4 [file 44318_2024_330_MOESM8_ESM.zip › Figure 4 source data/Figure 4F/Figure 4F +EGF Rab11.tif]

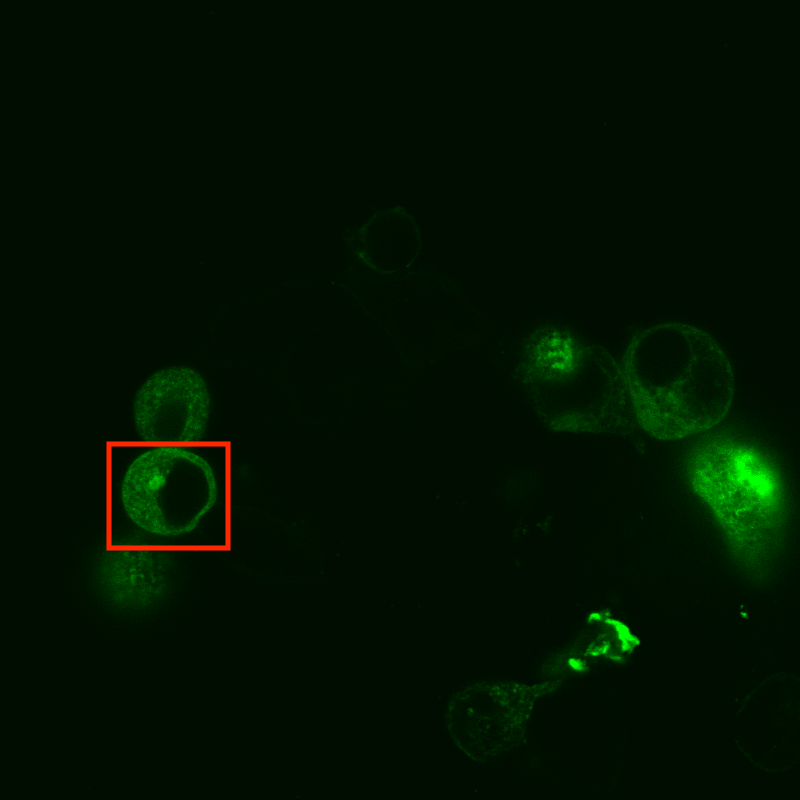

Supplement: Supplementary file 8 — Source data Fig. 4 [file 44318_2024_330_MOESM8_ESM.zip › Figure 4 source data/Figure 4F/Figure 4F -EGF CMTM4.tif]

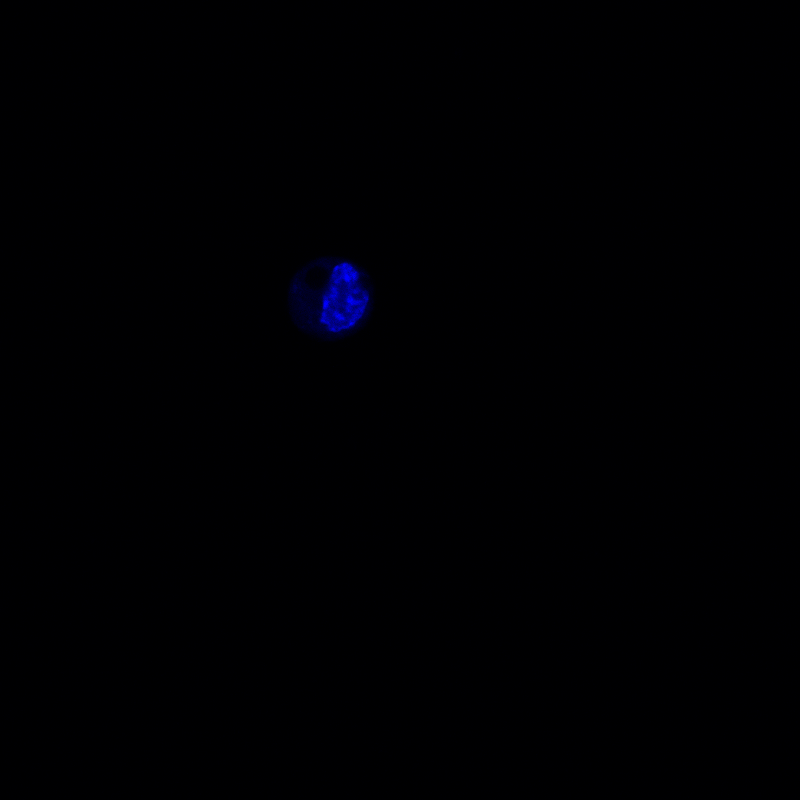

Supplement: Supplementary file 8 — Source data Fig. 4 [file 44318_2024_330_MOESM8_ESM.zip › Figure 4 source data/Figure 4F/Figure 4F +EGF DAPI.tif]

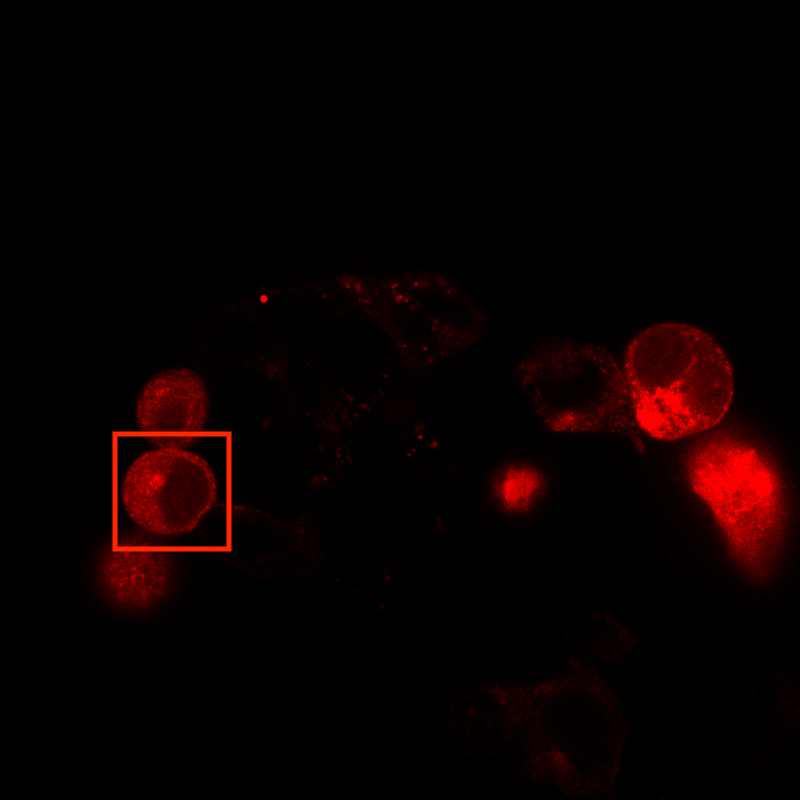

Supplement: Supplementary file 8 — Source data Fig. 4 [file 44318_2024_330_MOESM8_ESM.zip › Figure 4 source data/Figure 4F/Figure 4F -EGF Rab11.tif]

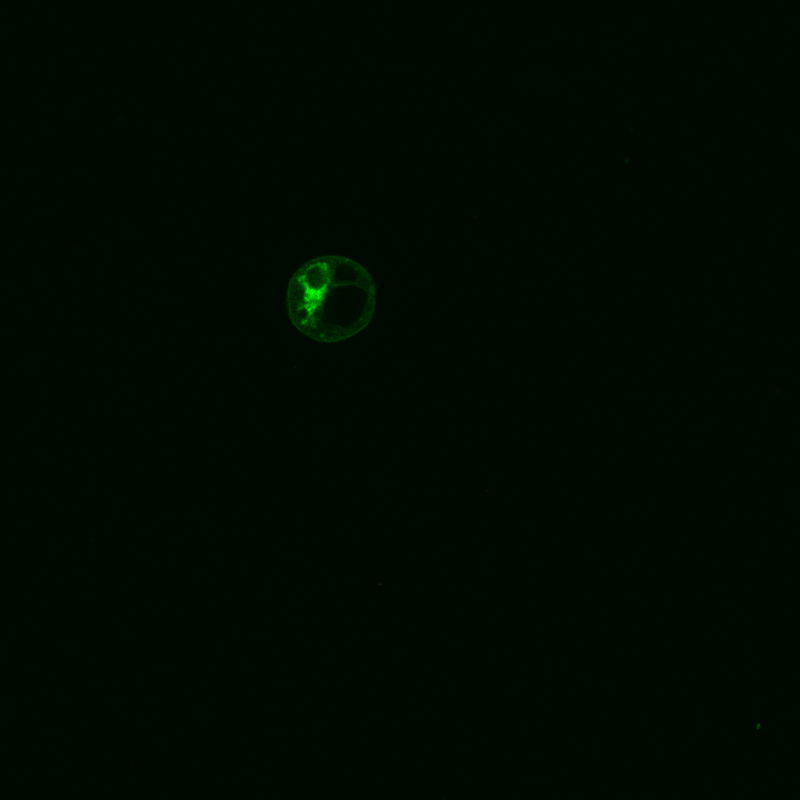

Supplement: Supplementary file 8 — Source data Fig. 4 [file 44318_2024_330_MOESM8_ESM.zip › Figure 4 source data/Figure 4F/Figure 4F +EGF CMTM4.tif]

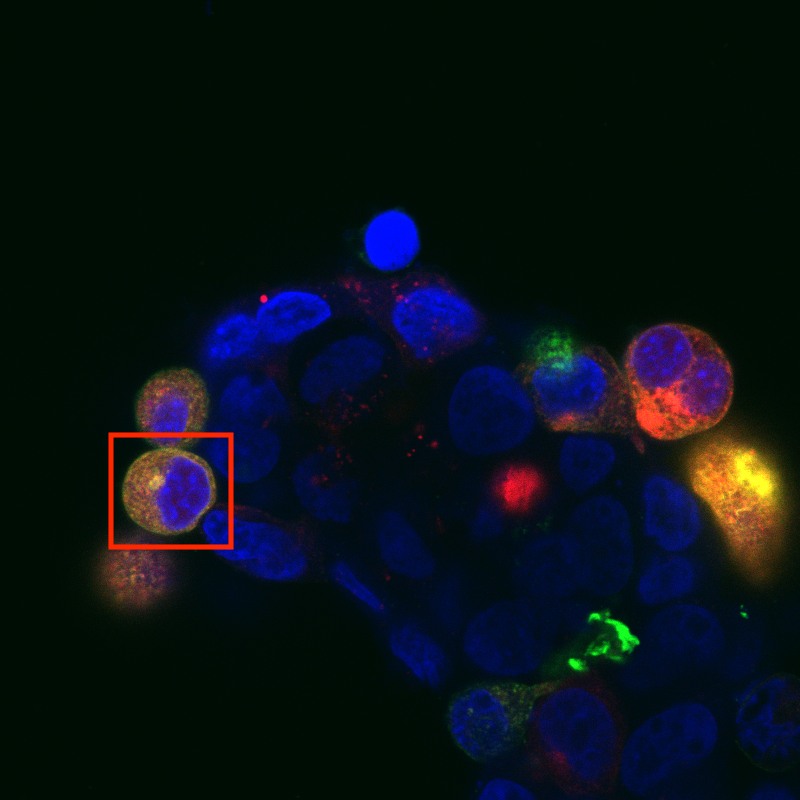

Supplement: Supplementary file 8 — Source data Fig. 4 [file 44318_2024_330_MOESM8_ESM.zip › Figure 4 source data/Figure 4F/Figure 4F -EGF Merged.tif]

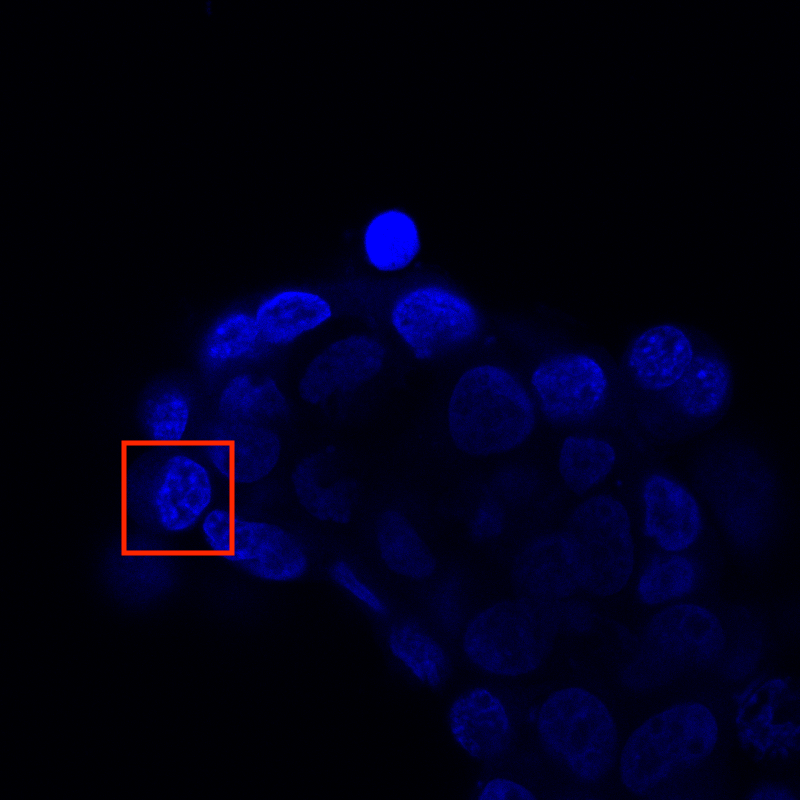

Supplement: Supplementary file 8 — Source data Fig. 4 [file 44318_2024_330_MOESM8_ESM.zip › Figure 4 source data/Figure 4F/Figure 4F -EGF DAPI.tif]

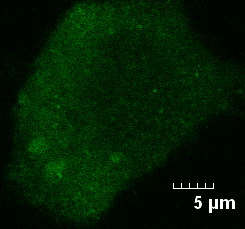

Supplement: Supplementary file 8 — Source data Fig. 4 [file 44318_2024_330_MOESM8_ESM.zip › Figure 4 source data/Figure 4H/Figure 4H KD+CQ EGFR.tif]

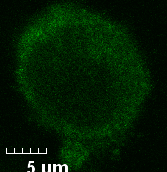

Supplement: Supplementary file 8 — Source data Fig. 4 [file 44318_2024_330_MOESM8_ESM.zip › Figure 4 source data/Figure 4H/Figure 4H CT EGFR.tif]

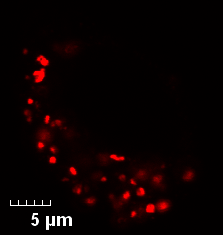

Supplement: Supplementary file 8 — Source data Fig. 4 [file 44318_2024_330_MOESM8_ESM.zip › Figure 4 source data/Figure 4H/Figure 4H KD LAMP1.tif]

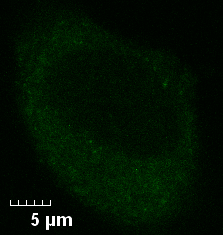

Supplement: Supplementary file 8 — Source data Fig. 4 [file 44318_2024_330_MOESM8_ESM.zip › Figure 4 source data/Figure 4H/Figure 4H KD EGFR.tif]

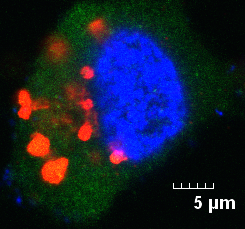

Supplement: Supplementary file 8 — Source data Fig. 4 [file 44318_2024_330_MOESM8_ESM.zip › Figure 4 source data/Figure 4H/Figure 4H KD+CQ Merged.tif]

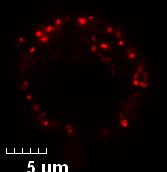

Supplement: Supplementary file 8 — Source data Fig. 4 [file 44318_2024_330_MOESM8_ESM.zip › Figure 4 source data/Figure 4H/Figure 4H CT LAMP1.tif]

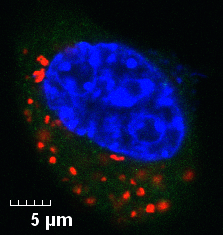

Supplement: Supplementary file 8 — Source data Fig. 4 [file 44318_2024_330_MOESM8_ESM.zip › Figure 4 source data/Figure 4H/Figure 4H KD Merged.tif]

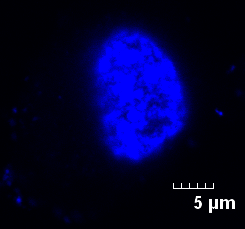

Supplement: Supplementary file 8 — Source data Fig. 4 [file 44318_2024_330_MOESM8_ESM.zip › Figure 4 source data/Figure 4H/Figure 4H KD+CQ DAPI.tif]

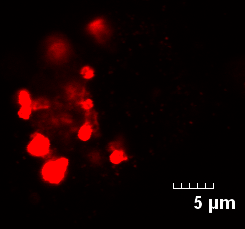

Supplement: Supplementary file 8 — Source data Fig. 4 [file 44318_2024_330_MOESM8_ESM.zip › Figure 4 source data/Figure 4H/Figure 4H KD+CQ LAMP1.tif]

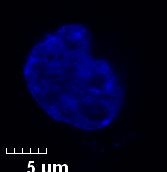

Supplement: Supplementary file 8 — Source data Fig. 4 [file 44318_2024_330_MOESM8_ESM.zip › Figure 4 source data/Figure 4H/Figure 4H CT DAPI.tif]

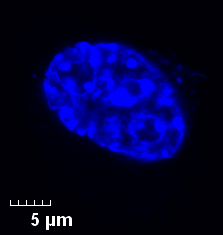

Supplement: Supplementary file 8 — Source data Fig. 4 [file 44318_2024_330_MOESM8_ESM.zip › Figure 4 source data/Figure 4H/Figure 4H KD DAPI.tif]

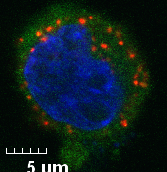

Supplement: Supplementary file 8 — Source data Fig. 4 [file 44318_2024_330_MOESM8_ESM.zip › Figure 4 source data/Figure 4H/Figure 4H CT Merged.tif]

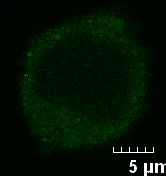

Supplement: Supplementary file 8 — Source data Fig. 4 [file 44318_2024_330_MOESM8_ESM.zip › Figure 4 source data/Figure 4G/Figure 4G KD EGFR.tif]

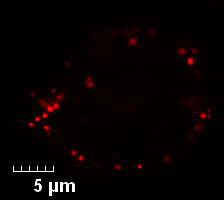

Supplement: Supplementary file 8 — Source data Fig. 4 [file 44318_2024_330_MOESM8_ESM.zip › Figure 4 source data/Figure 4G/Figure 4G CT LAMP1.tif]

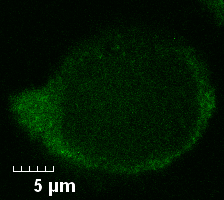

Supplement: Supplementary file 8 — Source data Fig. 4 [file 44318_2024_330_MOESM8_ESM.zip › Figure 4 source data/Figure 4G/Figure 4G CT EGFR.tif]

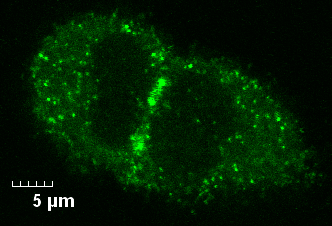

Supplement: Supplementary file 8 — Source data Fig. 4 [file 44318_2024_330_MOESM8_ESM.zip › Figure 4 source data/Figure 4G/Figure 4G KD+CQ EGFR.tif]

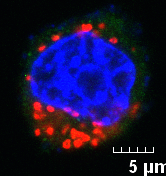

Supplement: Supplementary file 8 — Source data Fig. 4 [file 44318_2024_330_MOESM8_ESM.zip › Figure 4 source data/Figure 4G/Figure 4G KD Merged.tif]

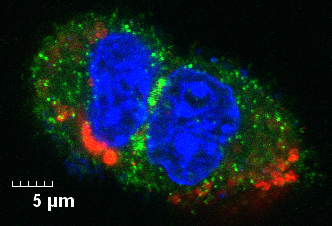

Supplement: Supplementary file 8 — Source data Fig. 4 [file 44318_2024_330_MOESM8_ESM.zip › Figure 4 source data/Figure 4G/Figure 4G KD+CQ Merged.tif]

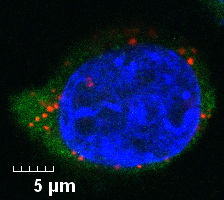

Supplement: Supplementary file 8 — Source data Fig. 4 [file 44318_2024_330_MOESM8_ESM.zip › Figure 4 source data/Figure 4G/Figure 4G CT Merged.tif]

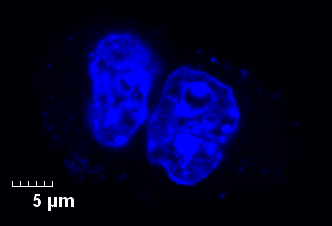

Supplement: Supplementary file 8 — Source data Fig. 4 [file 44318_2024_330_MOESM8_ESM.zip › Figure 4 source data/Figure 4G/Figure 4G KD+CQ DAPI.tif]

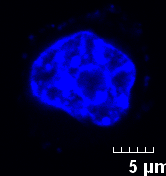

Supplement: Supplementary file 8 — Source data Fig. 4 [file 44318_2024_330_MOESM8_ESM.zip › Figure 4 source data/Figure 4G/Figure 4G KD DAPI.tif]

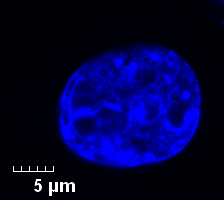

Supplement: Supplementary file 8 — Source data Fig. 4 [file 44318_2024_330_MOESM8_ESM.zip › Figure 4 source data/Figure 4G/Figure 4G CT DAPI.tif]

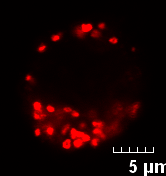

Supplement: Supplementary file 8 — Source data Fig. 4 [file 44318_2024_330_MOESM8_ESM.zip › Figure 4 source data/Figure 4G/Figure 4G KD LAMP1.tif]

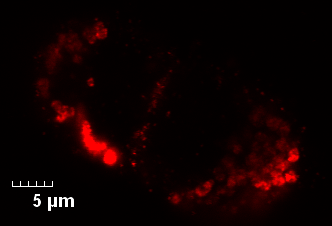

Supplement: Supplementary file 8 — Source data Fig. 4 [file 44318_2024_330_MOESM8_ESM.zip › Figure 4 source data/Figure 4G/Figure 4G KD+CQ LAMP1.tif]

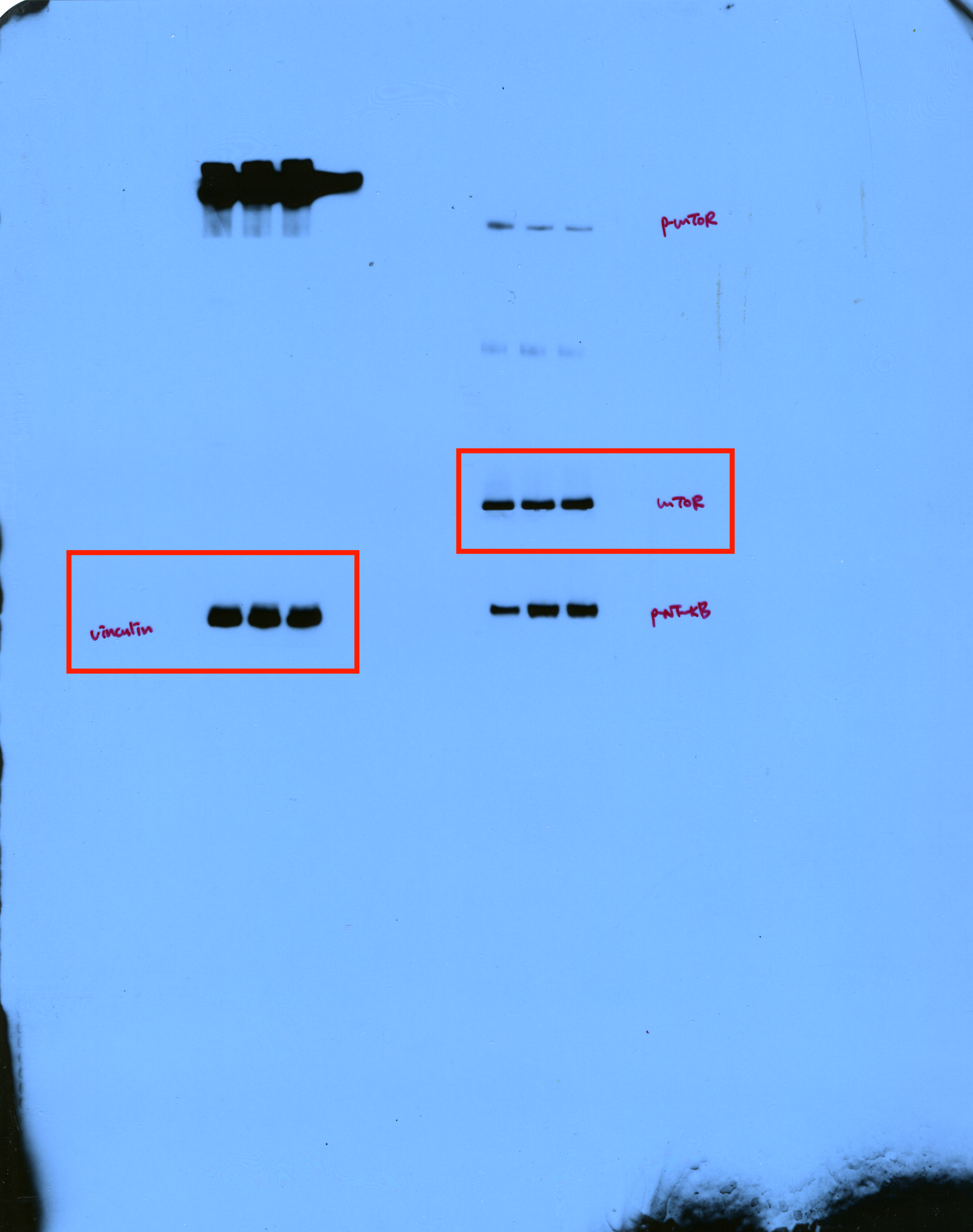

Supplement: Supplementary file 9 — Source data Fig. 5 [file 44318_2024_330_MOESM9_ESM.zip › Figure 5 source data/Figure 5A/Figure 5A mTOR, vinculin.tif]

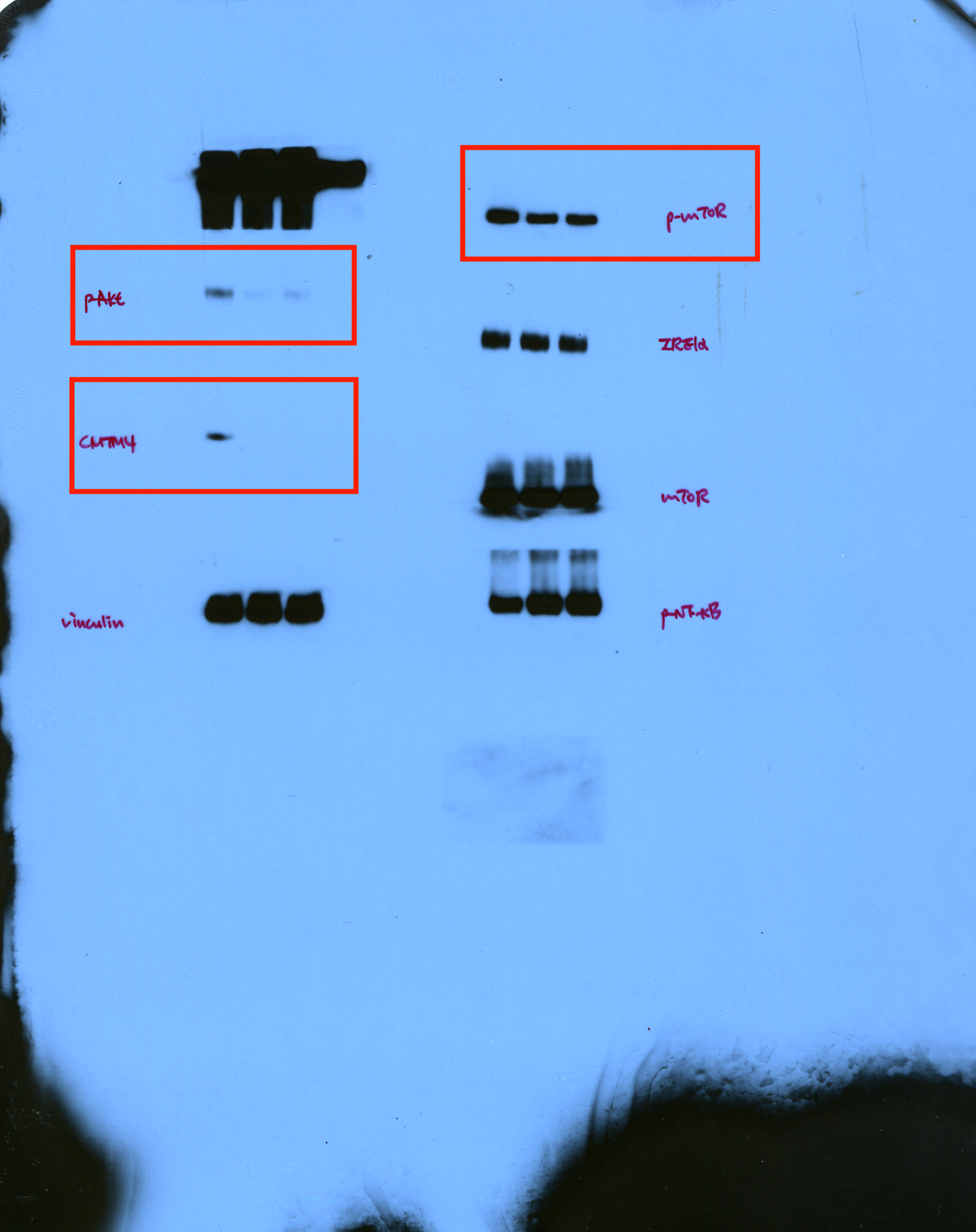

Supplement: Supplementary file 9 — Source data Fig. 5 [file 44318_2024_330_MOESM9_ESM.zip › Figure 5 source data/Figure 5A/Figure 5A pAkt, pmTOR, CMTM4.tif]

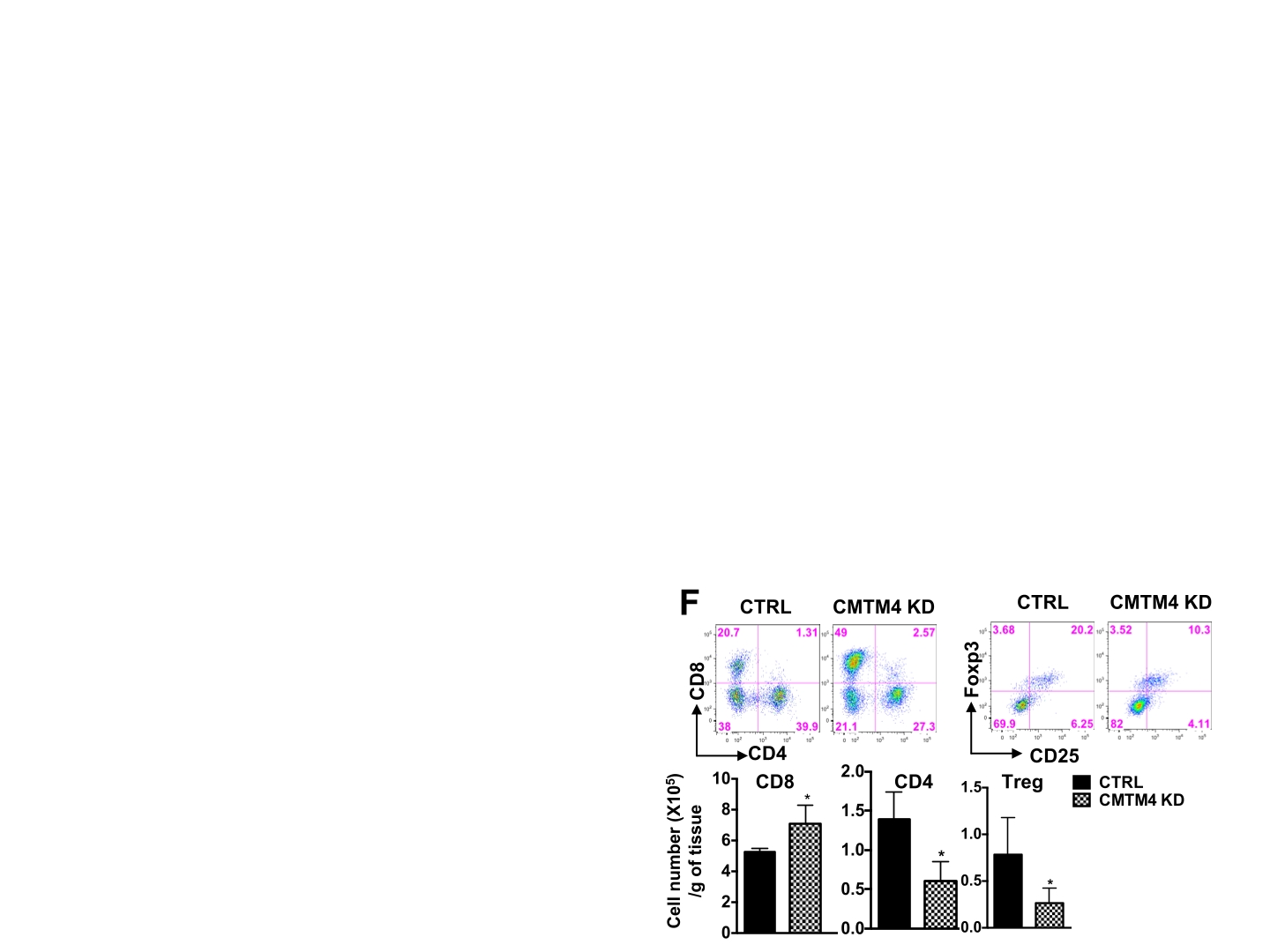

Supplement: Supplementary file 10 — Source data Fig. 6 [file 44318_2024_330_MOESM10_ESM.zip › Figure 6 source data/Figure 6F/Figure 6F.jpg]

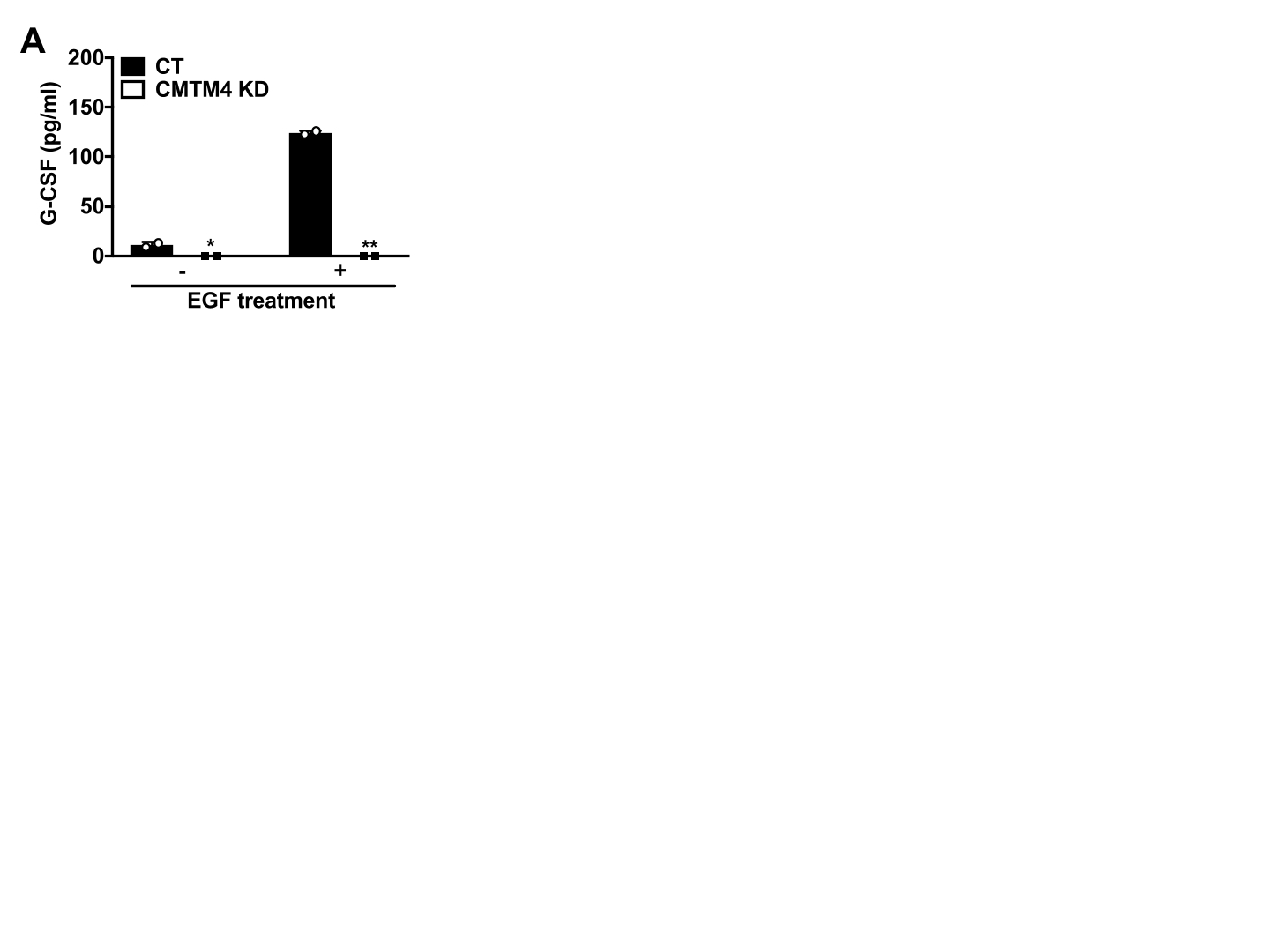

Supplement: Supplementary file 10 — Source data Fig. 6 [file 44318_2024_330_MOESM10_ESM.zip › Figure 6 source data/Figure 6A/Figure 6A.jpg]

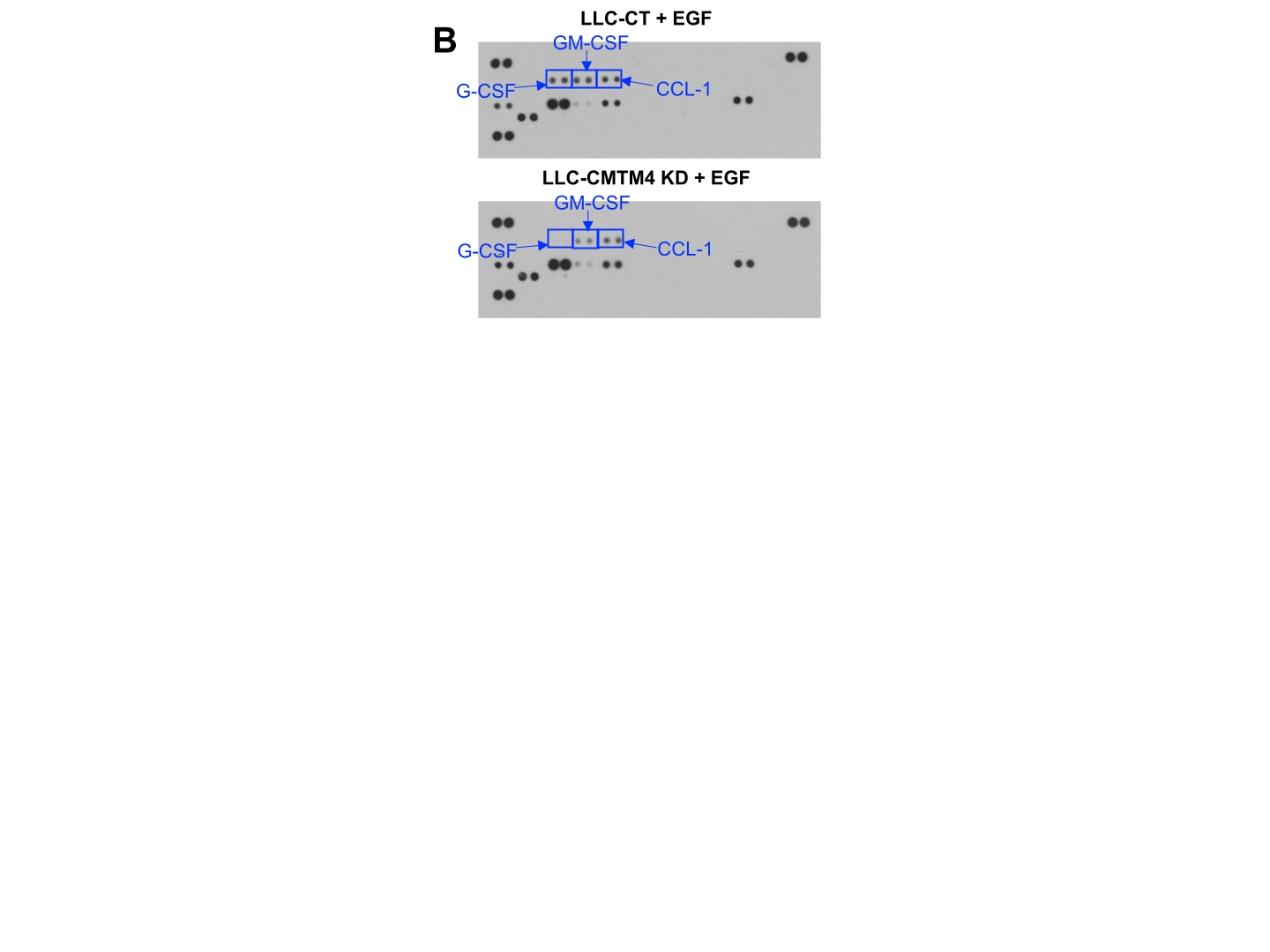

Supplement: Supplementary file 10 — Source data Fig. 6 [file 44318_2024_330_MOESM10_ESM.zip › Figure 6 source data/Figure 6B/Figure 6B.jpg]

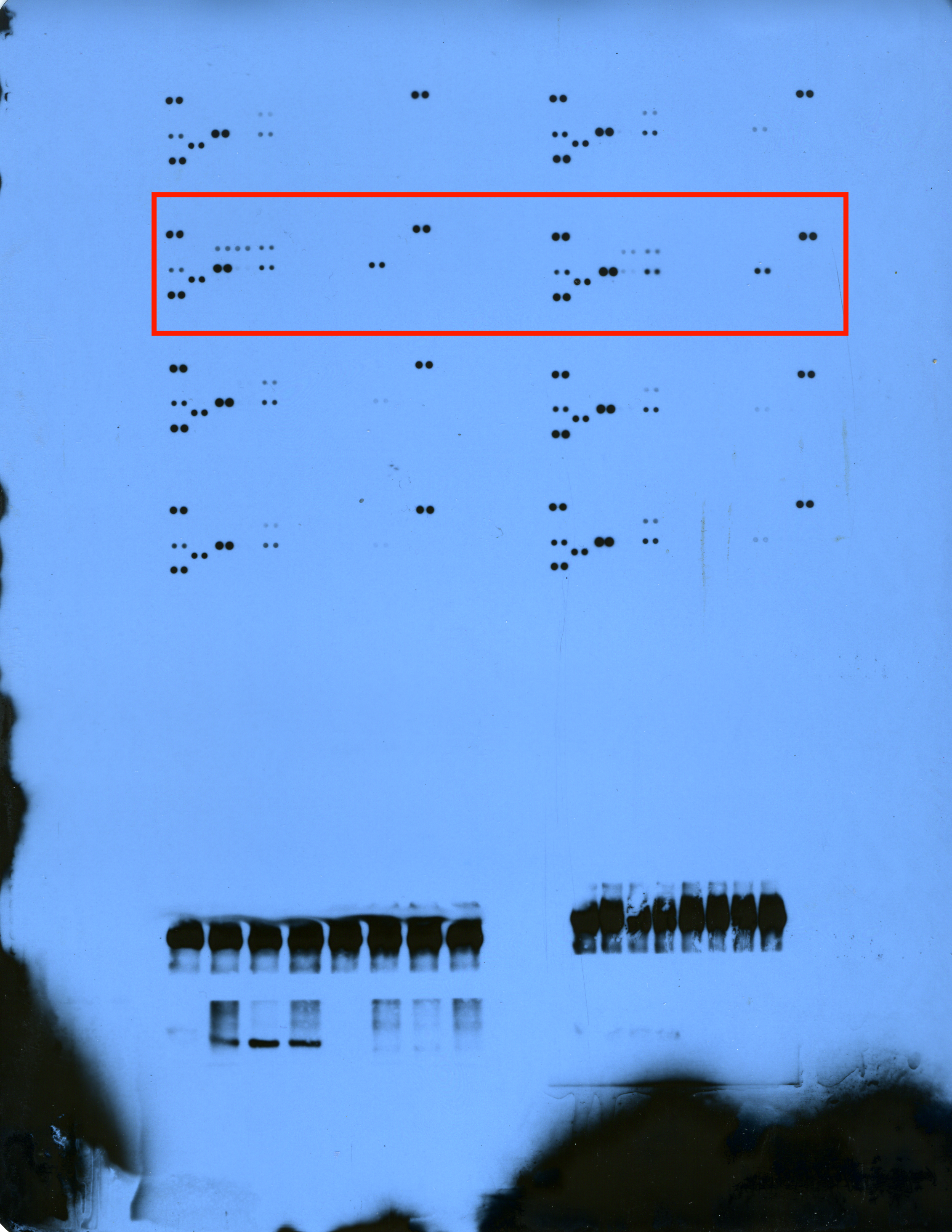

Supplement: Supplementary file 10 — Source data Fig. 6 [file 44318_2024_330_MOESM10_ESM.zip › Figure 6 source data/Figure 6B/Figure 6B.tif]

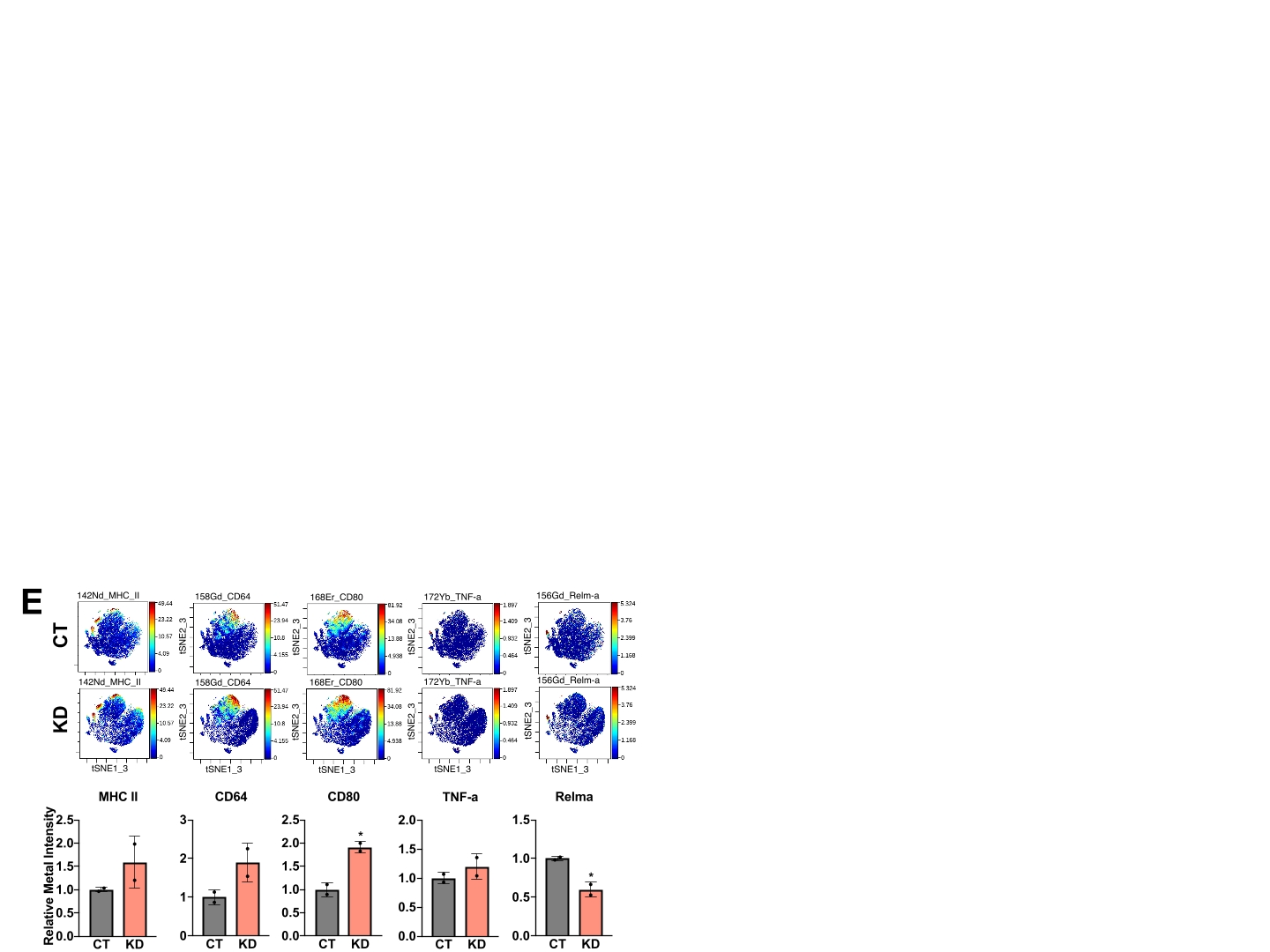

Supplement: Supplementary file 10 — Source data Fig. 6 [file 44318_2024_330_MOESM10_ESM.zip › Figure 6 source data/Figure 6E/Figure 6E.jpg]

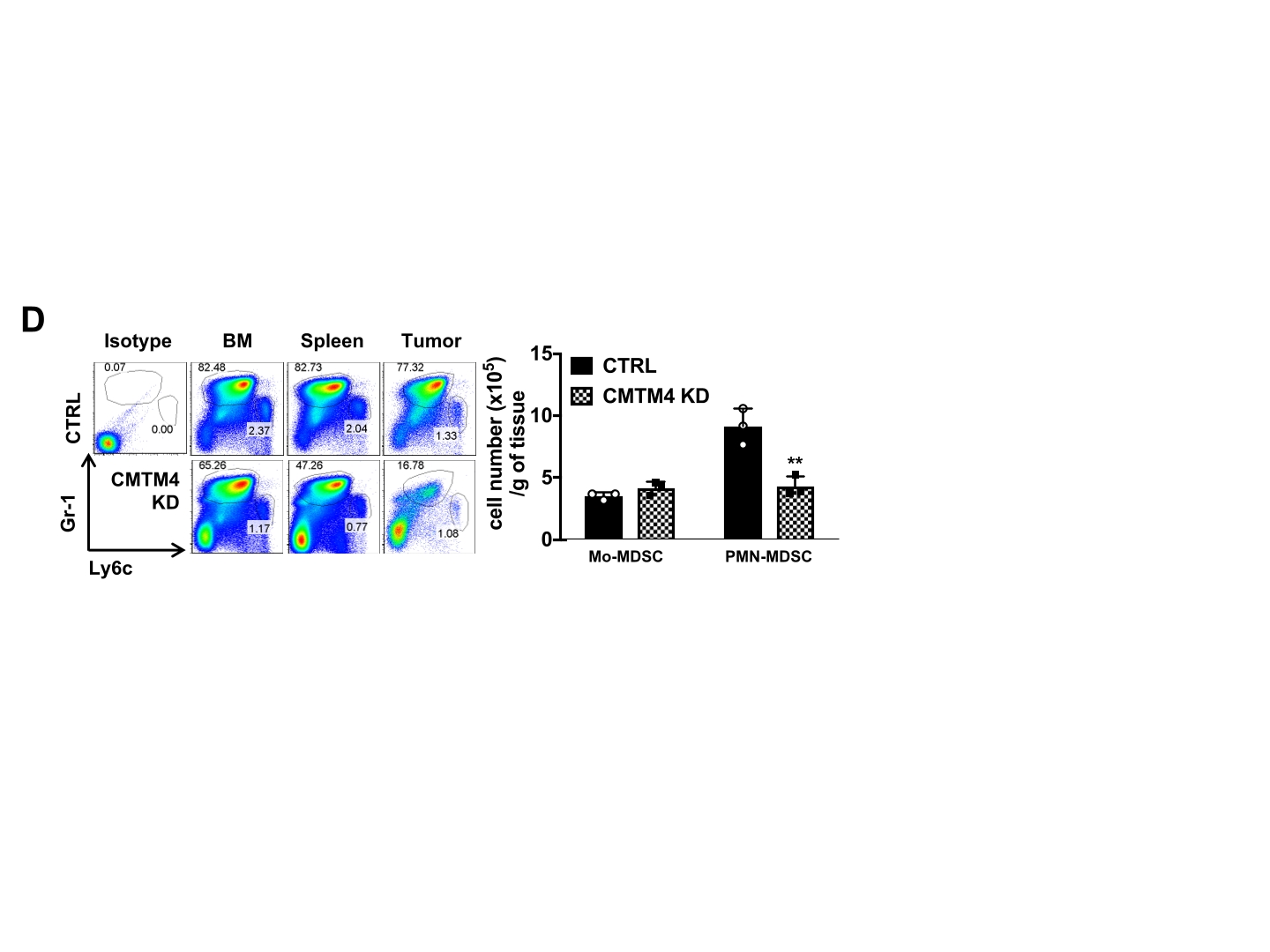

Supplement: Supplementary file 10 — Source data Fig. 6 [file 44318_2024_330_MOESM10_ESM.zip › Figure 6 source data/Figure 6D/Figure 6D.jpg]
