# Supplementary figures and images for: Modulation of tumor inflammatory signaling and drug sensitivity by CMTM4 (part 2 of 2)
Source: EMBO J. 2025 Feb 13;44(6):1866–83. doi: 10.1038/s44318-024-00330-y (PMC11914105; doi:10.1038/s44318-024-00330-y)

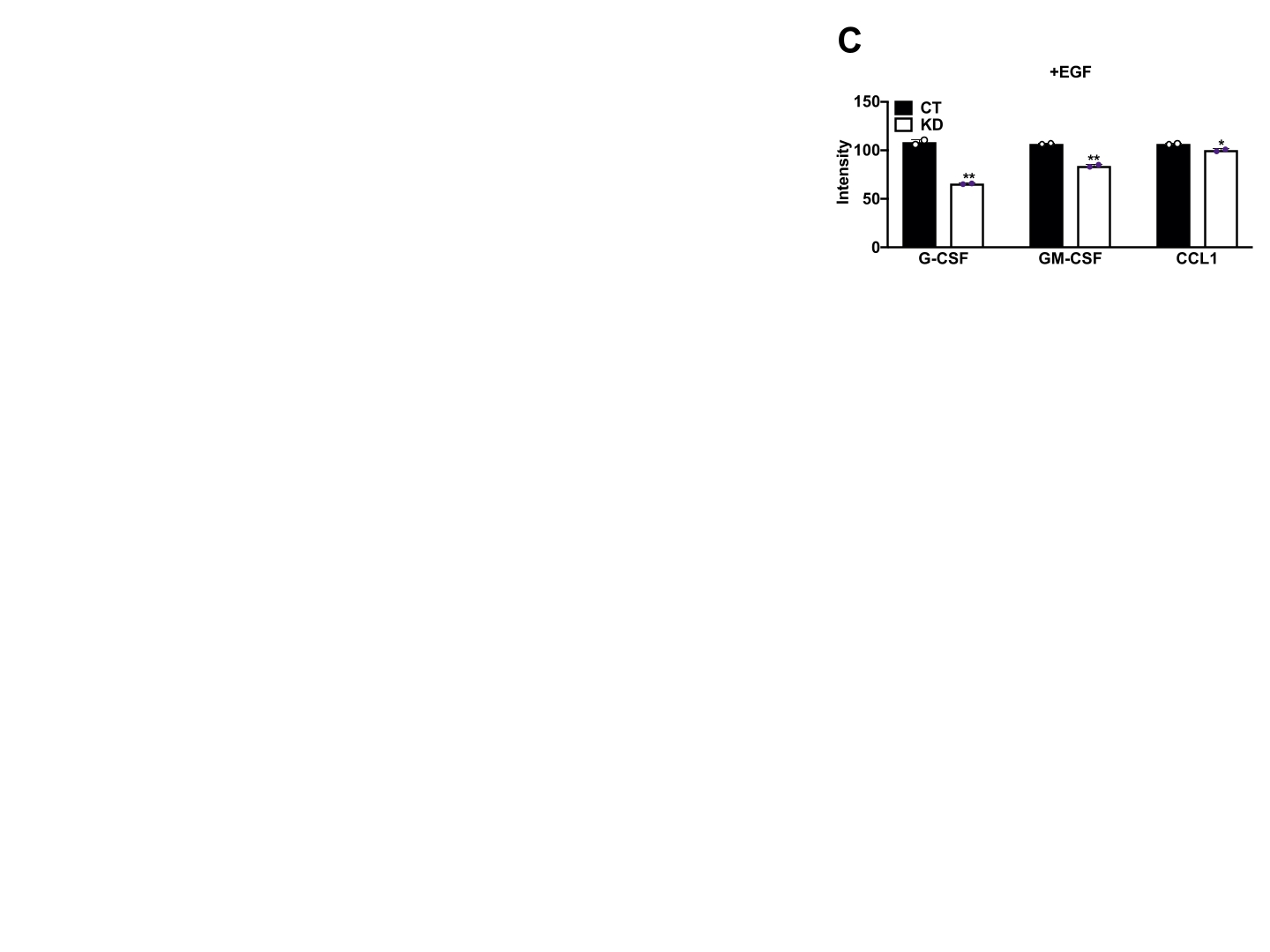

Supplement: Supplementary file 10 — Source data Fig. 6 [file 44318_2024_330_MOESM10_ESM.zip › Figure 6 source data/Figure 6C/Figure 6C.jpg]
